# Occupational Health Effects of Chlorine Spraying in Healthcare Workers: A Systematic Review and Meta-Analysis of Alternative Disinfectants and Application Methods

**DOI:** 10.3390/ijerph22060942

**Published:** 2025-06-16

**Authors:** Luca Fontana, Luca Stabile, Elisa Caracci, Antoine Chaillon, Kavita U. Kothari, Giorgio Buonanno

**Affiliations:** 1 Department of Civil and Mechanical Engineering, Università degli Studi di Cassino e del Lazio Meridionale (UNICAS), 03043 Cassino, Italy; l.stabile@unicas.it (L.S.); elisa.caracci@unicas.it (E.C.); buonanno@unicas.it (G.B.); 2 Center for AIDS Research (CFAR), University of California, San Diego, CA 92093, USA; achaillon@health.ucsd.edu; 3 Independent Researcher, Kobe 650-0017, Japan; kavitaukothari@hotmail.com

**Keywords:** disinfectants, occupational health, respiratory disorders, health personnel

## Abstract

Chlorine spraying was widely used during filovirus outbreaks, but concerns about occupational health risks led to a shift toward wiping. This systematic review aimed to evaluate the health risks associated with exposure to disinfectants among healthcare workers (HCWs), with a specific focus on chlorine-based products and spraying compared to alternative disinfectants and general disinfection tasks (GDTs). PubMed, Embase, and Scopus were searched from inception to March 2025. Eligible studies included observational or experimental research on HCWs exposed to chemical disinfectants. Two reviewers independently screened studies, assessed the risk of bias using a validated occupational health tool, and evaluated evidence certainty with the GRADE approach. Meta-analyses used fixed- and random-effects models; heterogeneity was assessed with I^2^ statistics. Out of 7154 records, 29 studies were included. Most studies were cross-sectional with a high bias risk. Odds ratios (ORs) were calculated using non-exposed groups as reference. Significant associations with respiratory conditions were found for chlorine-based products (OR 1.71), glutaraldehyde (OR 1.44), spraying (OR 2.25), and GDTs (OR 2.20). Exposure to chlorine-based products, glutaraldehyde, spraying, and GDTs likely increases respiratory risk in HCWs, as supported by moderate-certainty evidence. These findings support prioritizing safer disinfectants and strengthening protective measures over banning specific application methods.

## 1. Introduction

The *Filoviridae* family comprises two genera, Orthoebolavirus and Orthomarburgvirus, both of which have caused numerous outbreaks with high fatality rates over the past few decades [1]. Human-to-human transmission occurs through contact with an infected person’s body fluid. Infection prevention and control guidance from organizations like the U.S. Centers for Disease Control and Prevention and the World Health Organization (WHO) [2,3] used to recommend spraying 0.5% chlorine on surfaces and healthcare workers (HCWs) wearing personal protective equipment (PPE) who were in direct or indirect contact with the virus. Biocidal products used in healthcare settings undergo regulatory assessment prior to market authorization. Under EU Regulation No. 528/2012, this process includes the evaluation of toxicity and exposure to ensure that products do not present local or systemic health risks when used according to their approved conditions, including specified PPE and ventilation requirements.

However, over the last few years, the use of chlorine spraying has raised concerns about occupational health risks [4,5], prompting public health institutions to revise recommendations. WHO now bans direct spraying of HCWs and recommends chlorine wiping as the preferred disinfection method instead of spraying [6].

However, the systematic review supporting this decision found no evidence differentiating spraying from wiping in terms of efficacy or adverse health events. Consequently, the recommendation is primarily based on expert judgment and, as stated in the published guideline, on very low-certainty evidence [6]. Because wiping is labor-intensive and time-consuming, spraying remains a logistically attractive option [7], especially for HCWs in impermeable PPE working in hot, humid environments, where prolonged PPE use increases risks of heat stress and related injuries [8]. Additionally, the discomfort and decreased efficiency associated with PPE in high-temperature environments can pose health risks for staff [9].

Given the contrasting viewpoints and the limited evidence supporting changes to disinfection guidance, a systematic review was conducted to clarify the health risks associated with different disinfectants and application methods. Specifically, this review addresses two key research questions: (1) Are chlorine-based disinfectants more hazardous to HCWs than other types of disinfectants? (2) Does spraying disinfectants pose a greater risk to HCWs compared to other general disinfection tasks (GDTs) defined as all disinfection-related activities other than spraying, including wiping, mopping, disinfecting patient rooms, furniture surfaces, and equipment, as well as preparing and diluting products.

## 2. Materials and Methods

This systematic review followed the preferred reporting items for systematic reviews and meta-analysis (PRISMA-P) protocol [10]. The research protocol was registered a priori with the PROSPERO database (ID: CRD42023479363).

We searched PubMed, Scopus, and Embase on 15 November 2023 and updated the search on 2 March 2025 for full-text English articles without restricting the publication period. Additional studies were searched manually by examining the references of the included studies using ResearchRabbit^®^ [11]. The search strategy combined free-text and indexed terminology reflecting the eligibility criteria and was adapted for each database (Supplementary Materials, Table S1).

Eligibility criteria were based on the population (P), exposure (E), comparison (C), outcome (O), and study design (S) approach [12] as follows: P: HCWs exposed to chemical disinfectant products in occupational settings; E: occupational exposure to chlorine-based disinfectants or non-chlorine-based disinfectants; C: HCWs exposed to different disinfectants (chlorine-based versus non-chlorine-based), no disinfectants, or different application methods; O: occupational diseases or symptoms, such as respiratory conditions, respiratory symptoms, lung dysfunction, eye symptoms, skin symptoms, reproductive outcomes, and exposure markers; and S: case–control studies, cohort studies, cross-sectional studies, experimental studies, observational studies, case reports, and case series.

Qualitative studies, abstracts, conference papers, reviews, letters, and editorials were excluded. Full inclusion and exclusion criteria are detailed in Table S2 (Supplementary Materials).

### 2.1. Study Selection and Data Extraction

Two authors (LF and EC) independently screened titles, abstracts, and full texts against eligibility criteria. Data extraction was also conducted independently, with disagreements resolved through consultation with a third reviewer (GB or LS). Mendeley was used for reference management, and reasons for exclusion were recorded during the full-text review. A predesigned sheet (Supplementary Materials, Table S3) was used to extract and synthesize data on study characteristics, sample recruitment, exposure assessment, outcomes, and findings.

### 2.2. Risk of Bias and Quality of Evidence

Two authors (LF and EC) independently assessed the risk of bias (high, low, unclear) across eight domains using a validated occupational health tool [13,14,15]. Disagreements were resolved by discussion with a third reviewer (LS). The hybrid tool, incorporating elements from the Scottish Intercollegiate Guidelines Network [16] and Critical Appraisal Skills Program [17], is provided in Table S4 (Supplementary Materials).

### 2.3. Summary

This review primarily evaluates associations between occupational disinfectant exposure, including different application methods, and the incidence of occupational diseases.

### 2.4. Meta-Analysis

Studies were grouped by intervention: four groups for disinfectants (chlorine-based products; glutaraldehyde; peracetic acid [PAA], acetic acid [AA] and hydrogen peroxide [HP]; and quaternary ammonium compounds [QACs]), two groups for application methods (use of spray and GDTs), and one group for mitigation measures such as indoor ventilation and PPE which were included for completeness.

Health outcomes were clustered based on their relevance as respiratory, ocular-nasal, neurological, gastrointestinal, reproductive, immunological, and skin conditions (see “Outcome clustering” in Supplementary Materials). Meta-analysis was conducted when at least two primary studies with similar exposures and outcomes were available. When a study reported multiple outcomes, they were combined to create a single pairwise comparison [18] to avoid unit-of-analysis errors (see “Single pairwise comparison” in the Supplementary Materials).

The fixed-effects and random-effects models were used to generate pooled effect sizes. Higgins I^2^ statistic quantified the proportion of variability due to heterogeneity, while tau-squared (τ^2^) measured the between-study variance. Model selection was based on the I^2^ statistic, with significant heterogeneity defined as I^2^ ≥ 50% [19]. τ^2^ complemented this by contextualizing the variability in true intervention effects and informing the interpretation of random-effects models. Parameters were estimated using the Restricted Maximum Likelihood method with the metafor R package 4.3.2. [20]. Egger’s test and funnel plots were used to assess publication biases.

Meta-regression was conducted to examine the impact of study design and sample size on the observed heterogeneity. Residual heterogeneity was assessed, and the significance of the moderators was tested using the omnibus test for moderators (QM statistic) [21]. The R^2^ statistic quantified the proportion of heterogeneity explained by the model. Subgroup and sensitivity analyses were performed to explore potential sources of heterogeneity based on outcome type (e.g., asthma-related vs. nonspecific symptoms) and methodological differences and to evaluate their impact on overall results and heterogeneity. Subgroup analyses by disinfectant type and application method were pre-specified based on anticipated differences in exposure and toxicological profiles. Additional subgroup analyses, such as those based on outcome type, were exploratory and informed by observed heterogeneity. To address potential concerns of non-independence, evaluate the robustness, and determine the importance of individual studies on the overall meta-analysis results, a leave-one-out sensitivity analysis was conducted. To compare the risks associated with chlorine-based products to other disinfectants—and spraying to GDTs—the relative odds ratio (ROR) was calculated. Statistical analyses were performed using R version 4.3.2.

The certainty of evidence was assessed using the GRADE approach [22] across five domains: risk of bias, inconsistency, indirectness, imprecision, and publication bias. Conclusions were framed according to GRADE recommendations: terms such as “is”, “does”, “has”, or “will” for high-certainty evidence, “probably” or “likely” for moderate certainty, “may” or “the evidence suggests” for low certainty, and “very uncertain” for findings with very low certainty (see “GRADE” in the Supplementary Materials for more details).

## 3. Results

**Figure 1:**
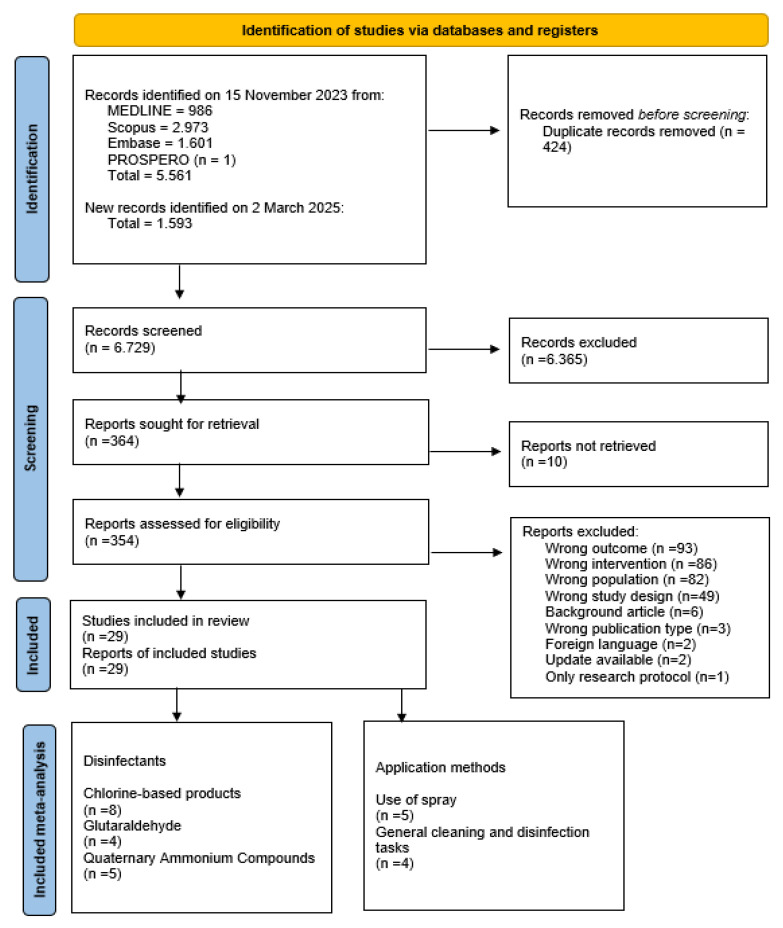
Flow diagram of literature search and selection criteria adapted from Preferred Reporting Items for Systematic Reviews and Meta-Analyses (adapted from Moher et al. [23]).

A total of 7154 articles were retrieved from the database search. After removing duplicates, 6729 articles remained for screening. Of these, 364 articles were eligible for full-text review. Ten full texts could not be retrieved and were excluded. After applying eligibility criteria, 29 studies were included (Figure 1). The data are synthesized in Table 1. Study categorization, quantitative data, and exclusion reasons are available in Supplementary Material Figure S1, Tables S5 and S6. Of the included studies, there were sixteen cross-sectional, six cohort, two case-control, two case series, two case reports, and one mixed-method experimental/observational study. Additionally, 6 studies had a low risk of bias, while 23 had a high risk (Supplementary Materials, Table S7). Most cross-sectional studies were deemed high risk due to their retrospective design, relying on self-reported surveys, which introduce potential biases related to outcome source and validation.

### 3.1. Chlorine-Based Products

Twelve studies examined the occupational health effects of chlorine-based product exposure (Table 1). Most were cross-sectional, with two cohort and one case–control study. The majority had a high risk of bias due to the retrospective design and reliance on self-reported exposure and outcome data, while three had a low risk. Dumas et al. [32] and Patel et al. [50] and Dumas et al. [29] reported a significant association between bleach exposure and poor asthma control [32], new asthma onset [50], and chronic obstructive pulmonary disease (COPD) [29]. Similarly, Su et al. categorized HCWs based on asthma symptoms and exposure to cleaning and disinfection activities, identifying a strong association between chlorine product use and undiagnosed/untreated asthma and asthma attacks/exacerbations [51]. However, earlier and later studies found no significant association between bleach exposure and new-onset asthma [28,30,37] or other respiratory conditions such as chest tightness and shortness of breath [35,47]. Mwanga et al. reported a significant association between bleach exposure exceeding 100 min per week and ocular–nasal symptoms, while no association was found with work-related asthma [44]. During the 2014–2016 Ebola outbreak, direct chlorine spraying on HCWs was common. Mehtar et al. found that multiple chlorine exposures were significantly associated with increased respiratory, eye, and skin conditions [5]. Similarly, Kobos et al. identified an association between bleach use and skin disorders or allergic reactions [39].

**Figure 2:**
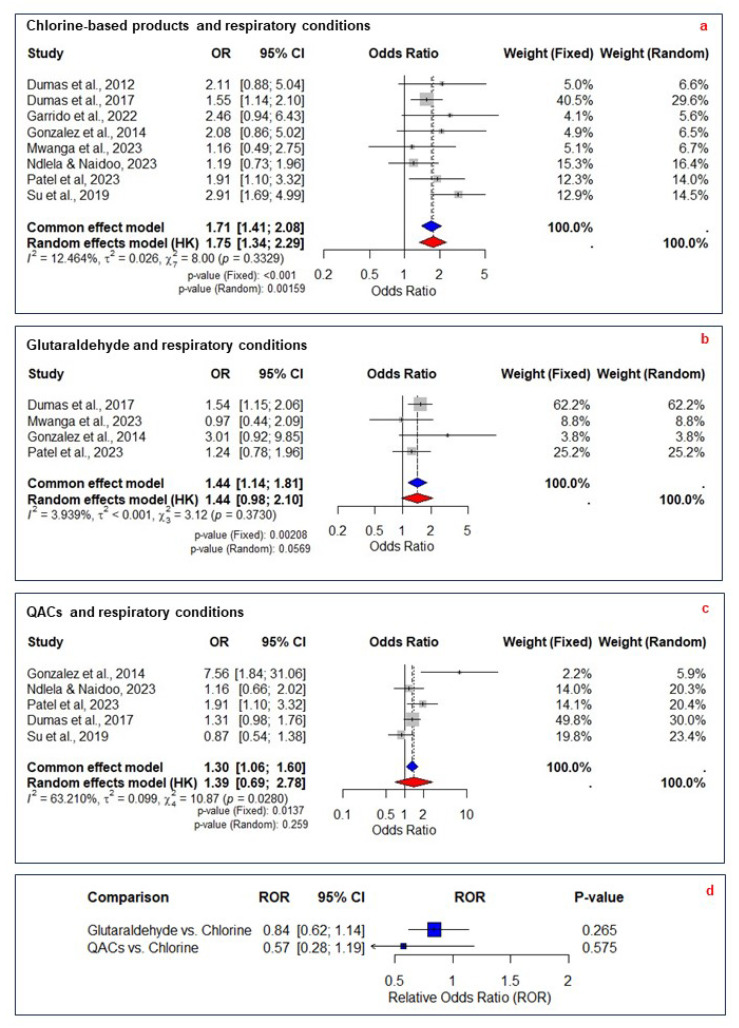
Meta-analysis of the association between disinfectants and respiratory conditions. (**a**) Forest plot of studies examining the association between exposure to chlorine-based products and respiratory conditions compared to a non-exposed group [28,32,35,37,44,46,50,51]. (**b**) Forest plot of studies assessing the association between glutaraldehyde exposure and respiratory conditions compared to a non-exposed group [32,37,44,50]. (**c**) Forest plot of studies examining the association between exposure to quaternary ammonium compounds (QACs) and respiratory conditions compared to a non-exposed group [32,37,46,50,51]. (**d**) Relative odds ratios (RORs) comparing the respiratory effects of glutaraldehyde and QACs to chlorine-based products. Blue diamond indicates the pooled OR from the common (fixed) effect model; red diamond indicates the pooled OR from the random effects model. Chlorine-based products and glutaraldehyde showed statistically significant associations with increased respiratory risk. The association for QACs was not statistically significant and showed high heterogeneity. OR, odds ratio; CI, confidence interval; ROR, relative odds ratio; QACs, quaternary ammonium compounds.

The meta-analysis was limited to respiratory conditions due to the availability of primary studies with similar exposures and outcomes. The analysis on chlorine-based products included eight studies with 7123 participants, comparing exposed and non-exposed groups across 11 respiratory effects. Outcomes from Ndlela et al. and Su et al. were combined into a single pairwise comparison (Supplementary Materials, Table S8) [46,51]. Four studies were excluded [29,30,39,44], with the reasons detailed in Supplementary Materials, Table S9. The fixed-effect model estimated an OR of 1.71 (95% CI 1.41–2.08, *p* < 0.001), while the random-effects model yielded an OR of 1.75 (95% CI 1.34–2.29, *p* = 0.002), with low heterogeneity (I^2^ = 12.5%, τ^2^ = 0.03, *p* = 0.33) (Figure 2a). The symmetrical funnel plot and Egger’s test (*p* = 0.45) indicated no substantial publication bias (Supplementary Materials, Table S10). The meta-regression analysis showed a negative coefficient for cross-sectional study design, suggesting that studies with this design type reported a slightly lower effect estimate (Supplementary Materials, Table S11). Leave-one-out analysis confirmed the stability of the effect estimate, with ORs ranging from 1.59 to 1.84 in the fixed-effect model (Supplementary Materials, Table S12). The evidence was rated as moderate certainty (Table 2).

### 3.2. Glutaraldehyde

Ten studies examined the occupational health effects of glutaraldehyde exposure (Table 1). Most were cross-sectional, with three cohort studies, one case series, and one mixed-methods study. Six had a high risk of bias due to the retrospective design and reliance on self-reported exposure and outcome data, while four had a low risk. Three studies applied air sampling techniques and found that glutaraldehyde can cause adverse health effects even below occupational limits, particularly occupational asthma [32,34], COPD [29], skin and airway symptoms, and headaches [48]. Additionally, poor work practices appear to increase exposure risk [45]. However, other studies found no significant association between glutaraldehyde exposure and new-onset asthma [30,31,37,45,51]. While one study reported a significant association between glutaraldehyde exposure exceeding 100 min per week and work-related ocular–nasal symptoms, no association was found with work-related asthma [44].

Meta-analysis was limited to respiratory conditions due to the availability of primary studies with similar exposures and outcomes. The analysis on glutaraldehyde exposure included four studies with 6256 participants, comparing exposed and non-exposed groups across six respiratory effects. Mwanga et al. [44] reported outcomes for three exposure levels, which were combined into a single pairwise comparison (Supplementary Materials, Table S8). Six studies were excluded, with the reasons detailed in Supplementary Materials, Table S9. The fixed-effect model estimated an OR of 1.44 (95% CI 1.14–1.81, *p* < 0.01), while the random-effects model yielded an OR of 1.44 (95% CI 0.98–2.10, *p* = 0.57), with low heterogeneity (I^2^ = 3.93%, τ^2^ < 0.001, *p* = 0.37) (Figure 2b).

The symmetrical funnel plot and Egger’s test (*p* = 0.96) indicated no substantial publication bias (Supplementary Materials, Table S10). Meta-regression did not identify significant moderators (Supplementary Materials, Table S11). Leave-one-out analysis confirmed the stability of the effect estimate, with ORs ranging from 1.28 to 1.51 in the fixed-effect model (Supplementary Materials, Table S12). The evidence was rated as moderate certainty (Table 2).

### 3.3. Peracetic Acid, Acetic Acid, and Hydrogen Peroxide

Nine studies examined the occupational health effects of products containing PAA, AA, and HP (Table 1). Most were cross-sectional, with three cohort studies and one case report. Five had a high risk of bias, while four had a low risk. Two studies applied air sampling. Casey et al. reported a higher prevalence of watery eyes and over three times the rate of current asthma among workers in the highest exposure department despite HP and AA levels being below OSHA’s permissible exposure limits (PELs) [26].

Hawley et al. and Blackley et al. identified significant associations between disinfectant exposure and eye and airway symptoms, even at levels below occupational limits [24,38]. Kobos et al. found that HCWs using HP-based products were 2- to 6-fold more likely to report allergic reactions than those who did not [39]. Similarly, Dumas et al. [29] reported an increased risk of developing COPD for nurses exposed to HP [29]. However, other studies found no significant association between HP and asthma incidence [30,31,32] or respiratory issues related to PAA [49]. Meta-analysis was not conducted due to the lack of comparable primary studies.

### 3.4. Quaternary Ammonium Compounds

Nine studies examined the occupational health effects of QAC exposure (Table 1). Most were cross-sectional, with two cohort studies. Six had a high risk of bias due to the retrospective design and reliance on self-reported data, while three had a low risk. Gonzalez et al. reported a significant association between QAC exposure and asthma and nasal symptoms among HCWs [37]. Patel et al. [50] and Dumas et al. [29] also found a significant association between QAC exposure and new-onset asthma [50] and COPD [29]. Ndlela and Naidoo reported an increased risk of respiratory issues, particularly shortness of breath [46]. Conversely, other studies found no significant association between QAC exposure and respiratory symptoms [30,32,45,52]. Kobos et al. identified a significant increase in skin disorders and allergic reactions among QAC users [39].

The meta-analysis was limited to respiratory conditions due to the availability of primary studies with similar exposures and outcomes. The analysis included five studies with 9270 participants, comparing exposed and non-exposed groups across nine respiratory effects. Ndlela et al. and Su et al. reported multiple outcomes, which were combined into a single pairwise comparison (Supplementary Materials, Table S8) [46,51]. Four studies were excluded, with the reasons detailed in Supplementary Materials, Table S9. The fixed-effect model estimated an OR of 1.30 (95% CI 1.06–1.60, *p* = 0.01), while the random-effects model yielded an OR of 1.39 (95% CI 0.69–2.78, *p* = 0.259), with significant heterogeneity (I^2^ = 63.2%, τ^2^ = 0.099, *p* = 0.03) (Figure 2c). The symmetrical funnel plot and Egger’s test (*p* = 0.29) indicated no substantial publication bias (Supplementary Materials, Table S10). Meta-regression did not identify significant moderators (Supplementary Materials, Table S11). Leave-one-out analysis confirmed that the effect estimate remained stable (fixed-effect model OR 1.22–1.44), except when Patel et al. [50] (*p* = 0.08) and Dumas et al. [32] (*p* = 0.09) were omitted (Supplementary Materials, Table S12). The evidence was rated as very low certainty due to the risk of bias, significant heterogeneity, and wide confidence intervals crossing the null value (Table 2).

### 3.5. Other Disinfectants

Six studies evaluated the occupational health risks from exposure to other disinfectants. Mwanga et al. reported a 4-fold increase in ocular–nasal symptoms with frequent use of alcohol-based products (OR 4.56), with similar risks for orthophthalaldehyde (OR 3.40), enzymatic cleaners (OR 2.57), and chlorhexidine (OR 1.84) [44]. Su et al. identified asthma risks associated with high-level disinfectants, alcohols, enzymes, formaldehyde, detergents, glass cleaners, and phenolic products [51]. Laborde-Castérot et al. linked aerosolized EDTA to respiratory conditions, with 10 of 28 patients showing positive nasal provocation tests [41]. Mac Hovcová et al. found disinfectants to be the most common chemical agents causing allergic skin diseases, though specific products were not identified [43]. Similarly, Nettis et al. identified disinfectant components as major triggers of occupational allergic contact dermatitis [47].

### 3.6. Relative Odds Ratios for Disinfectants

We assessed the RORs of respiratory conditions associated with different disinfectants, using chlorine-based products as the reference. The RORs were derived from ORs previously calculated against the non-exposed group. The ROR for glutaraldehyde relative to chlorine-based products was 0.84 (95% CI 0.62–1.14, *p* = 0.26), while for QACs, it was 0.81 (95% CI 0.39–1.68, *p* = 0.57) (Figure 2d). Both RORs were rated as low certainty due to the risk of bias and imprecision in pooled ORs (Table 2).

### 3.7. Application Methods

Eight studies assessed the occupational health risks associated with spray use and general disinfection tasks (Table 1). All were cross-sectional. Six had a high risk of bias, while two had a low risk. Lee et al. found that medium spray exposure (0.5–2 h/day with PPE) was significantly associated with respiratory conditions, while high exposure (>2 h/day without PPE) was not. High-exposure spraying was linked to chemical-related symptoms. Other application methods, such as mopping and wiping, were not significantly associated with respiratory or chemical-related symptoms at medium and high exposure levels. A variety of cleaners, degreasers, finishers, sealers, and polishes were used in the study setting [42]. Caridi et al. reported that cleaning and disinfecting fixed surfaces was significantly associated with current asthma, moderate exacerbation, and bronchial hyper-responsiveness [25]. Kurth et al. reported a significant association between cleaning/disinfection tasks and asthma or asthma-like symptoms [40]. Mwanga et al. reported that spray use, compared to wiping, was associated with nearly 5-fold higher odds of having a higher asthma symptom score. Manual sterilization and disinfection of medical instruments were associated with work-related ocular–nasal symptoms, though no details on specific disinfectants were provided [44]. Dumas et al. [28] found significant associations between moderate-to-high exposure (at least once a week) to GDTs and spray use with current asthma [28]. Mehtar et al. found that multiple versus single exposure to chlorine spray was associated with an increase in respiratory, eyes, and skin conditions [5]. Gonzalez et al. found that new-onset asthma among HCWs was significantly associated with GDTs and disinfectant dilution but not spray use [37]. Conversely, Patel et al. found that the use of spray for surface disinfection was significantly associated with new asthma onset [50].

**Figure 3:**
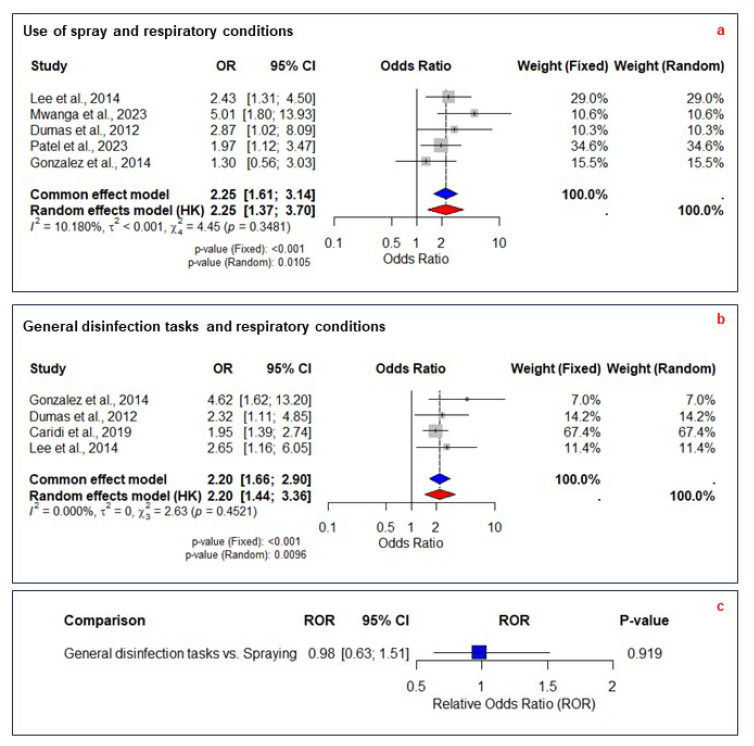
Meta-analysis of the association between disinfection methods and respiratory conditions. (**a**) Forest plot of studies examining the association between the use of spraying disinfectants and respiratory conditions compared to a non-exposed group [28,37,42,44,50]. (**b**) Forest plot of studies assessing the association between general disinfection tasks (e.g., wiping, mopping) and respiratory conditions compared to a non-exposed group [25,28,42,50]. (**c**) Relative odds ratios (RORs) comparing the respiratory effects of general disinfection tasks to spraying disinfectants. Blue diamond indicates the pooled OR from the common (fixed) effect model; red diamond indicates the pooled OR from the random effects model. Both spraying and general disinfection tasks were significantly associated with increased odds of respiratory conditions. The comparison between the two methods showed no significant difference. OR, odds ratio; CI, confidence interval; ROR, relative odds ratio.

Meta-analysis was limited to respiratory conditions due to the availability of primary studies with similar exposures and outcomes. The meta-analysis on spray use and respiratory conditions included five studies with 4568 individuals, comparing exposed and non-exposed groups across six adverse respiratory effects. Lee [42] reported separate health outcomes for medium and high exposure, which were combined into a single pairwise comparison (Supplementary Materials, Table S8). Mehtar’s [5] study was excluded, with the reasons detailed in Supplementary Materials, Table S9. The fixed-effects model estimated an OR of 2.25 (95% CI 1.61–3.14, *p* < 0.01), while the random-effects model yielded an OR of 2.25 (95 %CI 1.37–3.70, *p* = 0.010), with low heterogeneity (I^2^ = 10.18%, τ^2^ < 0.001, *p* = 0.35) (Figure 3a). Although the funnel plot showed some asymmetry, Egger’s test (*p* = 0.48) indicated no statistically significant publication bias (Supplementary Materials, Table S10). Meta-regression identified no significant moderators (Supplementary Materials, Table S11). Leave-one-out analysis confirmed stability, with ORs ranging from 2.05 to 2.49 in the fixed-effect model (Supplementary Materials, Table S12). The evidence was rated as moderate certainty, with minimal heterogeneity and stable estimates confirmed through sensitivity analyses (Table 2).

The meta-analysis on general disinfection tasks and respiratory conditions included four studies with 3480 individuals, comparing exposed and non-exposed groups across eight adverse respiratory effects.

Gonzalez, Caridi, and Lee [25,37,42], reported multiple outcomes, which were combined into a single pairwise comparison (Supplementary Materials, Table S8). Kurth [42] study was excluded, with the reasons detailed in Supplementary Materials, Table S9. The fixed-effect model estimated an OR of 2.20 (95% CI 1.66–2.90, *p* < 0.01), while the random-effects model yielded an OR of 2.20 (95% CI 1.44–3.36, *p* = 0.009), with no heterogeneity (I^2^ = 0%, τ^2^ = 0, *p* = 0.45) (Figure 3b).

Although the funnel plot showed some asymmetry, Egger’s test (*p* = 0.09) indicated no statistically significant publication bias. Meta-regression identified no significant moderators (Supplementary Materials, Table S11). Leave-one-out analysis confirmed stability, with ORs ranging from 2.08 to 2.82 in the fixed-effect model (Supplementary Materials, Table S12). The evidence was rated as moderate certainty, with no heterogeneity and consistent results confirmed across analyses (Table 2).

### 3.8. Relative Odds Ratios for Application Methods

We assessed the ROR of respiratory conditions associated with general disinfection tasks, using spray exposure as the reference. The ROR was 0.98 (95% CI 0.63–1.51, *p* = 0.9) (Figure 3c). The certainty of evidence was rated as low due to bias and imprecision in the pooled ORs.

### 3.9. Mitigation Measures

Five studies evaluated the effects of indoor ventilation on disinfectant exposure. Chang et al. found that adjusting air changes per hour (ACH) from 4 to 12–19 kept aerosolized chlorine dioxide levels below occupational limits, indicating minimal risk in ventilated rooms [27]. Norbäck reported that properly maintained ventilation maintained glutaraldehyde levels below Swedish limits, while poorly ventilated areas exceeded them, though specific ventilation rates were not provided [48]. Lee et al. found that continuous or frequent ventilation reduced the likelihood of respiratory or neurological symptoms in HCWs (OR 0.77, *p* < 0.05) [42]. Nayebzadeh et al. found no correlation between ACH and glutaraldehyde levels, suggesting that general ventilation alone was insufficient during solution changeover [45]. Estrin et al. assessed the concentration of ethylene oxide in the breathing zone of HCWs and concluded that it can cause neurological dysfunctions at low concentrations [33]. Multiple studies considered the use of PPE [39,42,44], but only one quantified the impact. Gaskins et al. assessed the impact of HLDs on fecundity in 1739 female nurses. HLD-exposed nurses using no PPE, one type of PPE, or two or more types of PPE experienced conception delays of 18%, 16%, and 0%, respectively. PPE use ranged from 9% for respiratory protection to 69% for gloves, suggesting that PPE can mitigate the reproductive risks of HLD exposure, though the composition of HLDs was not specified [36].

Certainty of evidence was assessed across five domains: risk of bias, inconsistency, indirectness, imprecision, and publication bias. Evidence was downgraded for high risk of bias, significant heterogeneity (I^2^ ≥ 50%), lack of alignment with the population, exposures, or outcomes of interest, wide confidence intervals crossing the null value, and evidence of publication bias from funnel plot asymmetry or Egger’s test. Certainty was classified as high, moderate, low, or very low based on the strength of evidence and identified limitations. For relative odds ratios, certainty was influenced by the quality and precision of pooled estimates, with downgrades applied when CIs included both harm and benefit.

## 4. Discussion

This systematic review and meta-analysis assessed the occupational health risks associated with exposure to various disinfectants and application methods among HCWs.

### 4.1. Disinfectants

Consistent with previous findings [52], the meta-analysis indicates that exposure to chlorine-based disinfectants likely increases the odds of developing respiratory conditions by 71% compared to non-exposed groups. No publication bias was detected, and leave-one-out analysis supported the stability of the results. Moreover, the evidence was assessed as moderate certainty. This underscores the importance of heightened precautions during outbreak responses, when the use of chlorine-based products is likely to increase. Additionally, the systematic review identifies non-respiratory health effects, including skin disorders, eye conditions, and allergic reactions, emphasizing the importance of comprehensive protective measures in occupational settings.

The meta-analysis shows that exposure to glutaraldehyde is likely to result in a 44% increase in the odds of developing respiratory conditions compared to non-exposed groups. This moderate-certainty evidence reflects reasonably strong confidence in the association despite some variability in study methodologies. Several studies support this finding: Gannon et al. reported occupational asthma at glutaraldehyde levels well below current exposure limits [34], and Dumas et al. [32] linked glutaraldehyde exposure to suboptimal asthma control [32]; this study contributed the largest weight in the meta-analysis due to its substantially larger sample size. Conversely, Gonzalez et al. [37] and later studies by Dumas et al. (2020, 2021) found no association with asthma incidence [30,31]. These results partially confirm previous findings [53], underscoring the need for further research. Beyond respiratory effects, studies also report associations with skin symptoms, headaches, and ocular–nasal irritation, highlighting broader health risks.

Due to the lack of primary studies with comparable exposures and outcomes, a meta-analysis was not conducted for peracetic acid, acetic acid, and hydrogen peroxide. Individual studies reported mixed findings. Casey et al. observed higher rates of watery eyes and current asthma among workers with the highest exposure levels to these chemicals [26]. Other studies, such as those by Otterspoor and Farrell, found no significant increases in respiratory issues or IgE levels [49]. This variability highlights the need for further research.

Exposure to QACs appears to increase the odds of developing respiratory conditions by 39% compared to non-exposed groups, but the evidence is very uncertain. High heterogeneity and wide confidence intervals further reduce the reliability of this pooled estimate. High heterogeneity likely reflects variability in effect sizes, study contexts, and outcome definitions. Subgroup analysis by outcome type (asthma-related vs. nonspecific symptoms) increased I^2^, indicating that outcome definitions alone do not fully explain the observed heterogeneity.

Sensitivity analysis revealed that excluding [37]—an outlier with a very high OR and wide confidence interval (7.56; 95% CI 1.84–31.05)—reduced heterogeneity to I^2^ = 36.3%. Even after omitting [37] the pooled OR for developing respiratory conditions remained significant at 1.25 (95% CI 1.01–1.55, *p* = 0.03), confirming the robustness of the meta-analysis results. The extreme effect size reported by [37] may reflect its unique study design, including a detailed occupational exposure assessment, small sample size, and a focus on high-risk tasks such as manual mixing of concentrated disinfectants. These factors likely amplify the observed association compared to studies relying solely on self-reported exposures or assessing broader healthcare populations. This combination of methodological and contextual differences likely explains the study’s disproportionate influence on heterogeneity. Individual studies presented mixed results. Gonzalez et al. found a significant risk of asthma associated with QACs [37], whereas Dumas et al. (2017, 2020) found no significant association with suboptimal asthma control or asthma incidence [30,32]. Kobos et al. reported an increased risk of skin disorders and allergic reactions [39], and Ndlela and Naidoo linked QAC exposure to respiratory issues [46]. These results highlight variability in study outcomes and underscore the need for further research.

The ROR for glutaraldehyde and QACs compared to chlorine-based products suggests 16% and 19% lower odds of developing respiratory conditions, respectively. However, the low certainty of evidence and the confidence intervals crossing the null value limit confidence in these findings, preventing firm conclusions.

### 4.2. Application Methods

The meta-analysis showed that spraying likely increases the odds of developing respiratory conditions by 125% (OR 2.25) compared to non-exposed groups, while general disinfection tasks likely increase the risk by 120% (OR 2.20). The ROR comparing GDTs to spraying suggests no significant difference in respiratory risk between application methods, but this finding is based on low-certainty evidence. This finding challenges the rationale for preferring wiping over spraying, suggesting that the focus should shift from prohibiting specific methods to improving overall safety measures. Enhancing ventilation, ensuring consistent PPE use, and selecting less hazardous disinfectants are likely to be more effective in reducing respiratory risks than banning spraying alone. The low certainty of evidence highlights some limitations, underscoring the need for more robust studies to clarify the relative risks of different disinfection methods.

Although respiratory symptoms were the most frequently reported adverse effects, few studies conducted air sampling to quantify exposure levels, complicating the interpretation of associations between disinfectant use and respiratory health outcomes. While a meta-analysis was not feasible, all studies consistently concluded that ventilation reduces airborne chemical concentrations, mitigating health risks. A similar protective effect is suggested for PPE use.

Limitations include the exclusion of non-English studies and potential misclassification of exposures and outcomes. Not all studies controlled for the same confounders, although low heterogeneity justified the use of a fixed-effects model. Underreporting of skin and ocular conditions limited broader assessment of disinfectant-related risks. Variations in exposure assessment and missing disinfectant concentration data complicated comparisons. Inconsistent reporting on disinfectants, ventilation, and PPE limited the attribution of health effects to specific chemicals, protective measures, or application methods. Moreover, very few studies provided both stratified exposure levels and clearly defined health outcomes, nor did they consistently describe whether biocidal products were used in accordance with manufacturers’ recommendations or regulatory guidelines. Detailed reporting on the use of PPE and risk mitigation measures was also lacking, which limits the ability to determine whether observed health effects reflect misuse or whether current guidance is insufficient to prevent harm. Respiratory outcomes were pooled across both nonspecific symptoms (e.g., cough) and clinically diagnosed conditions (e.g., asthma), which may limit the specificity of the effect estimates.

Recall bias remains a concern, given the retrospective design of most studies and the distinctive odors of disinfectants. The cross-sectional design of most studies limits causal inference between disinfectant exposure and respiratory outcomes. Nonetheless, the statistical significance of findings and consistency with prior research reinforce that disinfectant exposure, regardless of application method, poses an occupational health risk.

The increased risks associated with chlorine-based disinfectants compared to glutaraldehyde and QACs underscore the need for safer alternatives. Emerging evidence suggests that methylene blue may be an effective, less hazardous option, warranting further research [54,55]. The comparable risks between spraying and general disinfection tasks highlight the necessity of mitigation measures regardless of application method. These measures should include appropriate PPE, improved ventilation, and worker training on safe disinfection practices to minimize exposure.

Further prospective cohort studies with precise quantitative exposure and outcome assessments, including air sampling, are needed to clarify causal agents, environmental mechanisms, and how factors such as exposure duration, disinfectant concentration, PPE use, and ventilation contribute to risk variation.

## 5. Conclusions

This systematic review indicates that occupational exposure to chlorine-based products, glutaraldehyde, and QACs is associated with an increased risk of respiratory conditions compared to non-exposed groups. Among these, chlorine-based products likely pose the highest risk, supported by moderate-certainty evidence. Glutaraldehyde also likely increases the risk, with moderate-certainty evidence, despite some variability in study methodologies. In contrast, the evidence for QACs remains very uncertain due to high heterogeneity and limited data. Relative comparisons between disinfectants did not reveal statistically significant differences in respiratory risk; however, these findings are based on low-certainty evidence and should be interpreted with caution.

Both spraying and general disinfection tasks likely increase respiratory risk, supported by moderate-certainty evidence. Their relative comparison suggests nearly equal odds, though it is based on low-certainty evidence. These findings suggest that a blanket ban on spraying may not be justified. Instead, this review underscores the need for safer disinfectant alternatives and robust mitigation measures, including adequate ventilation, appropriate PPE, and strict adherence to safety protocols.

Efforts should prioritize replacing high-risk disinfectants with less hazardous alternatives whenever possible, especially in settings with poor ventilation. Respiratory protection must be ensured and correctly used in high-exposure procedures such as spraying. Regular assessment and improvement of ventilation systems are also critical. In parallel, comprehensive training and strict enforcement of safety protocols are essential to ensure safe handling and effective risk reduction.

Further research, particularly prospective cohort studies with quantitative exposure assessments, is needed to clarify the causal relationships between disinfectant exposure, application methods, and respiratory health outcomes.

## Figures and Tables

**Table 1:** Data synthesis for included studies.

Authors, Year, Study Design	Risk of Bias	Study Objective	Type of Recruitment, Population	Sample Size, Sex, Age	Exposure (Category), Assessment	Outcome (Cluster) Assessment	Adjustment Confounding	Main Findings
Blackley et al., 2023 [24] Cross-sectional	Low	To assess associations among exposures to PAA, AA, and HP and work-related eye and airway symptoms	Hospital staff performing cleaning duties and other staff in areas where cleaning occurred	67, 76% female, median age 47 years	Personal or mobile samples for HP, PAA, and AA; additional area samples (PAA, AA, and HP, MM)	Eye, skin, upper and lower airway symptoms assessed via post-shift survey (RC, ON)	Age, gender, smoking status, use of other products	PAA, AA, and HP associated with ON and RC
Caridi et al., 2019 [25] Cross-sectional	High	To investigate the association of asthma and related outcomes with occupations and tasks	Members of the Service Employees International Union	2030, 76% female, average age 48.6 years	Questionnaire on demographic characteristics, tasks performed, products used in healthcare occupations, and occurrence of asthma and related health outcomes (unspecified products)	Post-hire asthma, current asthma, exacerbation of asthma, BHR-related symptoms, asthma score, and wheeze (RC)	Gender, age, race, smoking status, allergies	Surface cleaning associated with RC
Casey et al., 2017 [26] Cross-sectional	High	To assess health effects of PAA, AA, and HP	Current staff of the hospital (volunteers)	163, 50 males, 113 females, 49 air samples	Air samples PAA, AA, and HP (PAA, AA, and HP)	Work-related symptoms, questionnaire (RC, ON)	Demographic, smoking status	PAA, AA, and HP associated with ON
Chang et al., 2018 [27] Case report	High	To assess the exposure of HCWs to airborne chlorine dioxide	HCWs who performed nasoendoscope disinfection	14 long-term personal air samples, 4 short-term personal air samples, 16 long-term area samples	ClO_2_ levels measured using ion-chromatograph after collection in midget impingers (chlorine, MM)	ClO_2_ concentrations were all below the OEL (RC)	N/A	Ventilation mitigates the risk
Dumas et al., 2012 [28] Cross-sectional	High	To determine the associations between asthma and occupational exposure to cleaning agents	HCWs and a reference population from the French cohort study (EGEA)	543, N/A, 18–79 years	Self-report, expert assessment, and asthma-specific job-exposure matrix (chlorine, spray, GDTs)	Asthma (RC)	Age, smoking status, BMI	Use of spray associated with asthma
Dumas et al., 2019 [29] Cohort	High	To investigate the association between exposure to disinfectants and COPD incidence in a large cohort of US female nurses	Participants from the Nurses’ Health Study II (NHSII)	73,262 females, age at baseline was 54.7 years	Occupational exposure to disinfectants, evaluated by questionnaire and a job–task–exposure matrix (JTEM)	Incident physician-diagnosed COPD evaluated by questionnaire	age, smoking (pack-years), race, ethnicity, and body mass index	Regular use of chemical disinfectants among nurses may be a risk factor for developing COPD
Dumas et al., 2020 [30] Cohort	High	To investigate the association between occupational exposure to disinfectants and asthma incidence in cohort of U.S. female nurses	Participants from the Nurses’ Health Study II (NHSII)	61,539 females, mean age 55 years at baseline	Occupational exposure to disinfectants evaluated by questionnaire and JTEM (chlorine, PAA, AA, HP, GU, QACs)	Incident physician-diagnosed asthma reported during follow-up (RC)	Age, race, ethnicity, smoking status, BMI	Disinfectants not associated with asthma
Dumas et al., 2021 [31] Cohort	High	To investigate the association between use of HLDs and asthma incidence	Participants from the Nurses’ Health Study 3 (NHS3)	17,280 female, mean age 34 years	Self-reported use of HLDs via questionnaire; duration of use; type of HLDs used in the past month; frequency of PPE use (chlorine, PAA, AA, HP, GU, QACs)	Incident clinician-diagnosed asthma reported during follow-up (RC)	Age, race, ethnicity, smoking status, BMI	HLDs associated with asthma
Dumas et al., 2017 [32] Cross-sectional	High	To examine the association between occupational exposure to disinfectants and asthma control in U.S. nurses	Participants from the Nurses’ Health Study II (NHSII)	4102 females, mean age 58 years	Occupational exposure to disinfectants evaluated by JTEM and self-reported disinfection tasks (chlorine, PAA, AA, HP, GU, QACs)	Asthma control measured using the Asthma Control Test (RC)	Age, smoking status, BMI, race, ethnicity	Disinfectants associated with poor asthma control
Estrin et al., 1987 [33] Case–control	High	To detect neurologic effects of chronic low-dose exposure to ethylene oxide	Hospital workers exposed to ethylene oxide and non-exposed controls	8, female, N/A	Hygienic measurements in the breathing zone, personal sampling (MM)	Psychometric test, nerve conduction studies, EEG spectral analysis, standardized neurologic examination (NC)	N/A	Ethylene oxide associated with neurologic dysfunction
Gannon et al., 1995 [34] Case series	High	To investigate cases of occupational asthma due to GU	Workers referred to a specialist occupational lung disease clinic	8, 7 females, 1 male, 29–53 years	Personal and static short and longer-term air samples, specific bronchial provocation tests (GU)	Occupational asthma by PEF measurements and specific bronchial provocation tests (RC)	N/A	GU associated with asthma
Garrido et al., 2022 [35] Case–control	High	To identify work tasks and cleaning/disinfecting agents associated with respiratory symptoms and hand dermatitis among HCWs in a tertiary hospital	Staff of three hospitals	230 exposed, 80% female, 77 control, 84% female, median age 44 years	Questionnaire on cleaning agent usage, respiratory symptoms, and skin symptoms; frequency of specific tasks and cleaning agents used (chlorine)	Self-reported respiratory symptoms and hand dermatitis (RC)	Age, sex	Disinfectants associated with RC and skin symptoms
Gaskins et al., 2017 [36] Cohort	High	To examine the relationship between occupational use of HLDs and fecundity among female nurses	Participants from the Nurses’ Health Study 3 (NHS3)	1739 females, mean age 33.8	Self-reported use of HLDs, frequency and duration of use, and use of PPE (MM) (multiple HLDs defined)	Duration of pregnancy attempt reported every six months	Age, BMI, smoking status, marital status, race	HLDs associated with reduced fecundity
Gonzalez et al., 2014 [37] Cross-sectional	High	To analyze associations between asthma and occupational exposure to disinfectants	Stratified random sampling of various healthcare departments	543, 59 males, 474 females, mean age 39.9 years	Occupational exposure assessment through a work questionnaire, workplace studies (chlorine, GU, QACs, spray)	Asthma, new-onset asthma, nasal symptoms at work, specific IgE assays (RC, ON)	Age, BMI, gender, smoking status, co-exposures	Disinfectant’s dilution and mixing associated with RC
Hawley et al., 2018 [38] Cross-sectional	Low	To assess respiratory symptoms in hospital cleaning staff exposed to PAA, AA, and HP	Hospital cleaning staff on all three shifts	50, 57% female, median age 40 years	Full-shift samples for HP, PAA, and AA; personal and mobile-area sampling; observation of cleaning tasks (PAA, AA, and HP)	Acute upper and lower airway symptoms from post-shift survey; chronic respiratory symptoms from extended questionnaire	Age, gender, and smoking status	PAA, AA, and HP associated with eye symptoms and RC
Kobos et al., 2022 [39] Cross-sectional	High	To characterize the prevalence of cleaning and disinfection product use, glove use during cleaning and disinfection, and skin/allergy symptoms by occupation	Current employees	559, 77% female, median age 49 years	Questionnaire on cleaning and disinfection product use, glove use, and skin/allergy symptoms (chlorine, PAA, AA, and HP, QACs, MM)	Prevalence of skin disorders and allergic reactions, glove use frequency (SC)	Age, sex, occupation, and product use frequency	Bleach, alcohol, and QACs associated with skin disorders
Kurth et al., 2017 [40] Cross-sectional	High	To estimate the prevalence of current asthma and asthma-like symptoms and their association with workplace exposures and tasks	Convenience sample	562, 78% female, mean age 46.5 years	Questionnaire on respiratory health, work characteristics, tasks performed, products used, and exposures (GDTs)	Self-reported current asthma, asthma-like symptoms, and breathing problems (RC)	Age, sex, race, smoking status, allergy	Disinfection tasks associated with RC
Laborde-Castérot et al., 2012 [41] Case series	High	To report cases of work-related rhinitis and asthma associated with exposure to EDTA-containing detergents or disinfectants	Patients with work-related rhinitis referred for NPT with EDTA	28, 21 females	History of exposure to aerosols of EDTA-containing products, NPT with tetrasodium EDTA (1–4%)	Positive NPT, presence of rhinitis symptoms, asthma-like symptoms, pulmonary function tests	N/A	EDTA associated with RC
Lee et al., 2014 [42] Cross-sectional	High	To investigate acute symptoms associated with chemical exposures among HCW’s work practices	Convenience sample of HCWs employed	183, 81 males, 102 females mean age 48 years	Self-reported data on chemical exposure, tasks performed, and use of PPE (spray, GDTs)	CRS (respiratory, eye, skin, neurological, gastrointestinal), interviews, or questionnaires (RC)	Age, sex, and job title	Use of spray and disinfectants associated with CRS
Mac Hovcová et al., 2013 [43] Cohort	High	To analyze the causes and trends in allergic and irritant-induced skin diseases in the healthcare sector	Data extracted from the National Registry of Occupational Diseases in the Czech Republic from 1997 to 2009	545, 95% female, mean age 38 years	Analysis of reported cases of occupational skin diseases, including patch testing and workplace hygiene evaluation	Prevalence and incidence of occupational skin diseases, trends over time, common causative agents	N/A	Disinfectants first cause of allergic skin diseases
Mehtar et al., 2016 [5] Cross-sectional	High	To determine the adverse effects of chlorine spray exposure on humans	Volunteers, including HCWs, Ebola survivors, and quarantined contacts	1550, 576 males, 974 females, 19–50 years	Self-reported chlorine spray exposure, frequency, and clinical condition post-exposure (chlorine, spray)	Prevalence of eye, respiratory, and skin conditions following chlorine exposure (RC, ON, SC)	Ebola disease effects on eyes	Spray of chlorine associated with eye, skin, and RC
Mwanga et al., 2023 [44] Cross-sectional	Low	To investigate occupational risk factors and exposure–response relationships for airway disease among HCWs exposed to cleaning agents	Stratified random sampling	699, 77% female, median age 42 years	Self-reported exposure to cleaning agents and related tasks, fractional exhaled nitric oxide testing, blood samples for atopy determination (chlorine, GU, QACs, spray)	ASS, WRONS, WRAS, FeNO levels (ON, RC)	Atopy, gender, smoking, age	Disinfectants and use of spray associated with RC
Nayebzadeh, 2007 [45] Mix method	High	To evaluate the impact of work practices and general ventilation systems on HCWs’ peak exposure to GU	HCWs from five hospitals in Quebec, Canada	42 personal samples, 53 HCWs interviewed	Breathing zone personal air samples, classified work practices, presence of local or general ventilation system (GU, MM)	Concentration of GU, exposure levels, prevalence of symptoms like headache and itchy eyes among HCWs	N/A	Work practices affect GU exposure
Ndlela & Naidoo, 2023 [46] Cross-sectional	Low	To investigate the relationship between exposure to cleaning and disinfecting agents and respiratory outcomes	Eligible cleaners from three public hospitals	174, 81% female, mean age 43.2 years	Self-reported frequency and duration of cleaning tasks and agent exposure, skin prick testing, spirometry (chlorine, QACs)	Respiratory symptoms, chest illnesses (asthma, tuberculosis, hay fever, chronic bronchitis), lung function measures (RC)	Sex, age, smoking history, any allergy, smoke	Disinfectant associated with RC
Nettis et al., 2002 [47] Cohort	High	To determine the prevalence and causes of occupational irritant and allergic contact dermatitis	HCWs referred to the Section of Allergy and Clinical Immunology at the University of Bari from 1994 to 1998	360, 280 females, 80 males; mean age 37.8 years	Patch testing with standard series and ‘health’ screening series, additional patch test with rubber allergens when necessary	Positive patch test reactions, diagnoses of allergic and irritant contact dermatitis	N/A	Disinfectants associated with allergic contact dermatitis
Norbäck, 1988 [48] Cross-sectional	Low	To study the prevalence of certain symptoms among HCWs with and without exposure to GU during cold sterilization	HCWs handling GU and a reference group of unexposed workers	107, 98 females	Hygienic measurements in the breathing zone (GU, MM)	Self-reported symptoms from a questionnaire, including eye, skin, and airway symptoms, headache, nausea, and fatigue (ON, RC)	Demographic data	Ventilation mitigates GU exposure; GU associated with RC
Otterspoor & Farrell, 2019 [49] Case report	High	To evaluate buffered PAA as an alternative to chlorine and HP	NA	20, N/A, N/A	Assessment of adverse staff reactions, safe-work related incident reporting (PAA, AA, and HP)	Acceptance, cost analysis, efficacy (RC)	N/A	PAA, AA, and HP higher acceptance than chlorine
Patel et al., 2023 [50] Cross-sectional	Low	To examine associations of cleaning tasks and products with WRAS in HCWs in Texas in 2016, comparing them to prior results from 2003	Representative sample of Texas HCWs from state licensing boards	2421, 83% female, average age 48.8 years;	Self-reported data on cleaning tasks, products used, and occupational exposures (chlorine, GU, QACs, spray)	Self-reported physician-diagnosed asthma, new onset asthma, work-exacerbated asthma, and bronchial hyperresponsiveness	Age, gender, race, atopy, obesity, smoking status, and years on the job	Use of spray, bleach, QACs associated with WRAS
Su et al., 2019 [51] Cross-sectional	High	To identify and group HCWs with similar patterns of asthma symptoms and explore their associations with patterns of cleaning and disinfecting activities (CDAs)	HCWs from nine selected occupations	2029, 1542 females, 487 males, N/A	Self-reported information on asthma symptoms/care, CDAs, demographics, smoking status, allergic status (chlorine, QACs)	Asthma symptom clusters and their associations with exposure clusters (ECs) through multinomial logistic regression (RC)	Age, gender, education, smoking status, and allergic status	Chlorine associated with RC

N/A = not available, NA = not applicable, HCWs = healthcare workers, OEL = occupational exposure limits, PAA = peracetic acid, AA = acetic acid, HP = hydrogen peroxide, GU = glutaraldehyde, PEF = peak expiratory flow, RC = respiratory conditions, MM = mitigation measure, ON = ocular–nasal conditions, SC = skin conditions, GDTs = General disinfection tasks, QACs = Quaternary ammonium compounds, PPE = personal protective equipment, CRS = Chemical-related symptoms, ASS = Asthma Symptom Score, WRONS = work-related ocular–nasal symptoms, WRAS = work-related asthma symptoms, HLDs = high-level disinfectants, BMI = body mass index, JTEM = job–task–exposure matrix, NPT = nasal provocation test, NC = neurological conditions.

**Table 2:** Summary of findings table. Assessment of evidence for the risk of studied outcomes based on the Grading of Recommendations, Assessment, Development, and Evaluation (GRADE) framework.

Intervention or Comparison	OR/ROR. (95% CI)	Quality of Study (Risk of Bias) High ↓	Inconsistency I^2^ > 50% ↓	Indirectness of Evidence ↓	Imprecision Cl Crossing 1 ↓	Publication Bias, Yes or Unclear ↓	Overall Certainty of Evidence
Chlorine-Based Products	OR: 1.71 (1.41–2.08)	Yes ↓ ^1^	None (-)	None (-)	None (-)	None (-)	Moderate
Glutaraldehyde	OR: 1.44 (1.14–1.81)	Yes ↓ ^1^	None (-)	None (-)	None (-)	None (-)	Moderate
QACs	OR: 1.39 (0.69–2.78)	Yes ↓ ^2^	Yes ↓ ^4^	None (-)	Yes ↓ ^5^	None (-)	Very Low
Glutaraldehyde vs. Chlorine	ROR: 0.84 (0.62–1.14)	Yes ↓ ^3^	Not applicable	None (-)	Yes ↓ ^5^	Not applicable	Low
QACs vs. Chlorine	ROR: 0.81 (0.39–1.68)	Yes ↓ ^3^	Not applicable	None (-)	Yes ↓ ^5^	Not applicable	Low
Use of spray	OR: 2.25 (1.61–3.14)	Yes ↓ ^1^	None (-)	None (-)	None (-)	None (-)	Moderate
GDTs	OR: 2.20 (1.66–2.90)	Yes ↓ ^1^	None (-)	None (-)	None (-)	None (-)	Moderate
GDTs vs. Spraying	ROR: 0.98 (0.63–1.51)	Yes ↓ ^3^	Not applicable	None (-)	Yes ↓ ^5^	Not applicable	Low

^1^ Variability in study quality, ^2^ variability in study quality and exposure assessments, ^3^ risk of bias in pooled ORs, ^4^ I^2^ = 63.2%, ^5^ CIs crossing the null value.

## Data Availability

The original contributions presented in this study are included in the article/Supplementary Materials. Further inquiries can be directed to the corresponding author(s).

## Supplementary Materials

The following supporting information can be downloaded at https://www.mdpi.com/article/10.3390/ijerph22060942/s1: Figure S1. Alluvial plot displaying the clustering of studies based on the intervention or exposure assessed and the associated health outcomes. Table S1. Search strategy. Table S2. Eligibility criteria. Table S3. Data extraction template for included studies. Table S4. Risk of bias instrument. Table S5. Quantitative data extracted from included studies. Table S6. Excluded studies and reasons for exclusion. Table S7. Risk of bias assessment for included studies. Table S8. Combined odds ratios (ORs) and confidence intervals (CIs) for respiratory conditions across studies. Table S9. Studies excluded from the meta-analysis and reasons for exclusion. Table S10. Funnel plots. Table S11. Meta-regression results. Table S12. Leave-one-out analysis results

## Abbreviations

The following abbreviations are used in this manuscript:

AA	Acetic Acid
ACH	Air Changes per Hour
CI	Confidence Interval
COPD	Chronic Obstructive Pulmonary Disease
CRS	Chemical-Related Symptoms
EC	Exposure Cluster
GDTs	General Disinfection Tasks
GRADE	Grading of Recommendations, Assessment, Development, and Evaluation
GU	Glutaraldehyde
HCWs	Healthcare Workers
HLDs	High-Level Disinfectants
HP	Hydrogen Peroxide
I^2^	I-squared (statistical measure of heterogeneity)
JTEM	Job–Task–Exposure Matrix
MM	Mitigation Measure
OEL	Occupational Exposure Limit
ON	Ocular–Nasal Condition
OR	Odds Ratio
PAA	Peracetic Acid
PECO	Population, Exposure, Comparator, Outcome
PEF	Peak Expiratory Flow
PPE	Personal Protective Equipment
PRISMA	Preferred Reporting Items for Systematic Reviews and Meta-Analyses
QACs	Quaternary Ammonium Compounds
RC	Respiratory Condition
ROR	Relative Odds Ratio
SC	Skin Condition
SOP	Standard Operating Procedure
