# Supplementary material for: Occupational Health Effects of Chlorine Spraying in Healthcare Workers: A Systematic Review and Meta-Analysis of Alternative Disinfectants and Application Methods
Source: Int J Environ Res Public Health. 2025 Jun 16;22(6):942. doi: 10.3390/ijerph22060942 (PMC12192869; doi:10.3390/ijerph22060942)
Supplement: Supplementary file 1 [file ijerph-22-00942-s001.zip › ijerph-3679810-supplementary.pdf]

Supplementary materials

Contents

Methodology 3

Outcome clustering 3

Single pair-wise comparison 5

GRADE 6

Figure S1. Alluvial plot displaying the clustering of studies based on the intervention or exposure assessed and the associated health outcomes..... 8

Table S1. Search strategy 9

Table S2. Eligibility criteria 13

Table S3. Data extraction template for included studies 14

Table S4. Risk of bias instrument 15

Table S5. Quantitative data extracted from included studies 20

Table S6. Excluded studies and reasons for exclusion 26

Table S7. Risk of bias assessment for included studies 37

Table S8. Combined Odds Ratios (ORs) and Confidence Intervals (CIs) for respiratory conditions across studies 40

Table S9. Studies excluded from the meta-analysis and reasons for exclusion 42

Table S10. Funnel plots 44

Table S11. Meta-regression results 47

Table S12. Leave-one-out analysis results 49

References 52

Methodology

Outcome clustering

Studies were categorized into groups based on the specific intervention assessed: four groups for disinfectants (chlorine-based products, glutaraldehyde, peracetic acid (PAA), acetic acid (AA), and hydrogen peroxide (HP), and quaternary ammonium compounds (QACs)); two groups for application methods (use of spray and general disinfection tasks (GDTs), defined as any other disinfection-related activities except spraying, such as wiping, mopping, disinfection of patient rooms, furniture surfaces, equipment, and preparation and dilution of products); and one group for mitigation measures (indoor ventilation and personal protective equipment (PPE)), which were included for completeness.

Health outcomes reported in the included studies were grouped into clusters based on their shared physiological pathways, clinical manifestations, and relevance to occupational exposure. This approach was necessary to streamline the meta-analytic process, minimize heterogeneity within outcome categories, and facilitate meaningful comparisons between studies. Below, the rationale for each outcome cluster is detailed:

Condition	Included outcomes	Theoretical basis	Relevance to disinfectant exposure
Respiratory Conditions	New-onset asthma, poorer asthma control, asthma exacerbations, undiagnosed/untreated asthma, wheeze, cough, phlegm, shortness of breath, breathlessness, throat irritation, difficulty breathing	The respiratory system is the primary target of airborne chemical exposures in occupational settings, particularly from disinfectants. Inhalation of irritants or sensitizers can trigger airway inflammation, bronchial hyperresponsiveness, and obstruction, which manifest as asthma, wheezing, coughing, and other related symptoms.	Chlorine-based products and other disinfectants are known to release volatile compounds that irritate the respiratory mucosa, leading to both acute and chronic respiratory conditions. This grouping captures both disease outcomes (e.g., asthma) and symptomatology (e.g., wheeze, cough) to reflect the full spectrum of respiratory effects.
Ocular-Nasal Conditions	Eye irritation, nasal irritation, sneezing, nasal obstruction,	The conjunctivae and nasal mucosa are highly sensitive to chemical irritants, making them common sites of	Many disinfectants, including chlorine-based products, release fumes or vapors that can irritate the eyes and nasal

	nasal catarrh, ocular-nasal symptoms	inflammation and irritation following exposure to airborne disinfectants. These symptoms are often the first indicators of exposure to volatile compounds.	passages, causing tearing, redness, nasal congestion, and sneezing. These effects often co-occur, justifying their grouping under a single category.
Skin Conditions	Skin disorders, eczema, rashes, allergic skin reactions.	The skin is a common site of occupational exposure during disinfection tasks, especially when proper protective measures (e.g., gloves) are not used. Direct contact with disinfectants can lead to skin irritation or allergic dermatitis.	Both chlorine-based and non-chlorine-based disinfectants can act as irritants or allergens, leading to conditions such as eczema or contact dermatitis. Grouping these outcomes together reflects the shared mechanism of dermal exposure and reaction.
Neurological Conditions	Headache	Neurological symptoms, such as headaches, can result from systemic absorption of chemical vapors or indirect effects of irritant exposures (e.g., stress or discomfort caused by respiratory or ocular symptoms).	Headaches are frequently reported by workers exposed to volatile compounds released by disinfectants. Though less directly related to specific mechanisms of exposure than other clusters, they are an important indicator of occupational discomfort and systemic effects.
Gastrointestinal Conditions	Nausea	Inhalation of volatile compounds from disinfectants can irritate the gastrointestinal mucosa or trigger systemic effects, leading to nausea. Nausea may also result from the aversive	Nausea is a commonly reported symptom among healthcare workers exposed to chemical disinfectants and reflects the broader systemic effects of occupational exposure.

		odors of disinfectants, particularly in poorly ventilated environments.	
Immunological Conditions	Allergic reactions.	Allergic reactions occur as hypersensitivity responses triggered by exposure to sensitizing agents in disinfectants. These reactions often manifest systemically or as localized skin conditions (in that case, they are reported as skin conditions).	Certain disinfectants, such as quaternary ammonium compounds (QACs) or glutaraldehyde, are known allergens that can provoke immune responses in sensitive individuals. This grouping reflects the broader immunological effects of disinfectant exposure beyond localized reactions.

The clustering of outcomes was guided by:

- Physiological pathways: Outcomes within the same cluster share common exposure routes or biological mechanisms (e.g., inhalation for respiratory and ocular-nasal conditions, direct contact for skin conditions).
- Clinical presentation: Clustering reflects common patterns of symptoms or conditions that healthcare workers experience during occupational exposure, facilitating meaningful interpretation of results.
- Consistency across studies: Grouping similar outcomes ensures comparability across studies with diverse reporting practices, enabling consistent meta-analysis.
- Reduction of heterogeneity: By consolidating related outcomes, the grouping minimizes heterogeneity within clusters, improving the validity of pooled estimates in the meta-analysis.

Single pair-wise comparison

For studies reporting multiple outcomes or subgroups within the same population, a statistical approach was applied to combine these groups into a single pair-wise comparison to avoid unit-of-analysis errors. This method ensures that each study contributes only one effect size to the overall

meta-analysis. The process involved calculating a pooled effect estimate for the multiple groups or outcomes using inverse-variance weighting. Specifically, the log-transformed effect sizes (e.g., odds ratios) and their corresponding standard errors were extracted for each group. The combined log-effect size was calculated as the weighted average of the individual log-effect sizes, with weights proportional to the inverse of the squared standard errors. The combined standard error was then derived from the total inverse variance, ensuring that the pooled estimate reflected the precision of the individual groups. The pooled log-effect size was back-transformed to the original scale (e.g., odds ratio) for interpretation, and 95% confidence intervals were calculated to represent the uncertainty around the combined estimate.

GRADE

The Grading of Recommendations, Assessment, Development, and Evaluations (GRADE) approach (The GRADE Working Group, 2013) was applied systematically to assess the certainty of evidence across all interventions and comparisons included in this review. This methodology evaluates the quality of evidence by considering five key domains: risk of bias, inconsistency, indirectness, imprecision, and publication bias. Each domain was assessed to determine its influence on the confidence in the pooled results.

The assessment of risk of bias focused on the methodological quality of the included studies. Variability in study design, reliance on self-reported outcomes, and the potential for bias inherent to cross-sectional designs were carefully evaluated. A hybrid tool, combining elements from the Scottish Intercollegiate Guidelines Network (SIGN) and the Critical Appraisal Skills Program (CASP), was used to categorize the risk of bias as high, low, or unclear.

To evaluate inconsistency, we examined the variability in effect estimates across the included studies. The Higgins I^2 statistic was employed to quantify the extent of heterogeneity. When the I^2 value was below 50%, inconsistency was deemed minimal, and no downgrades were applied. However, if the I^2 value exceeded 50%, it was considered indicative of significant heterogeneity, resulting in a downgrade of the certainty of evidence.

The indirectness domain assessed the relevance of the evidence to the research questions posed in this review. Studies were evaluated to confirm alignment with the population of interest (healthcare workers), the specific exposures (disinfectants and their application methods), and the outcomes of interest (respiratory conditions and other health effects). Studies directly addressing these aspects were not downgraded, while those with mismatches in relevance were penalized accordingly.

Imprecision was assessed by analyzing the confidence intervals (CIs) around the pooled effect estimates. Wide confidence intervals that crossed the null value (1.0) indicated uncertainty in the results, leading to a downgrade in certainty. Additionally, imprecision was influenced by the number of included studies and the total sample size, with small study pools contributing to less confidence in the findings.

Publication bias was evaluated through a combination of funnel plot analysis and Egger's test for asymmetry. Where no evidence of significant publication bias was detected (Egger's test p-value ≥ 0.05), no downgrades were applied. For derived comparisons, such as relative odds ratios (RORs), publication bias assessments were not applicable, as these comparisons relied on pooled estimates from prior meta-analyses rather than individual study-level data.

Based on the evaluation across these domains, the certainty of evidence was categorized into four levels: high, moderate, low, or very low. High-certainty evidence reflected strong confidence in the findings, while moderate-certainty evidence indicated some limitations in one domain. Evidence was classified as low or very low when significant concerns were identified across two or more domains.

Special considerations were applied to relative comparisons, such as RORs. These comparisons used pooled odds ratios (ORs) from individual meta-analyses to derive estimates, and heterogeneity and publication bias metrics were not calculated. Certainty for RORs was primarily influenced by the methodological quality of the underlying studies and the precision of the pooled estimates. In instances where confidence intervals for RORs included both harm and benefit, downgrades were applied for imprecision.

To enhance the clarity of conclusions, narrative statements were crafted according to GRADE recommendations. For high-certainty evidence, definitive terms such as 'is,' 'does,' 'has,' or 'will' were used to reflect very strong confidence in the findings. For moderate-certainty evidence, terms such as "probably" or "likely" were used to reflect reasonably strong confidence. Low-certainty evidence was conveyed using phrases like "may" or "the evidence suggests," while findings with very low certainty were described as "very uncertain." This phrasing allowed readers to clearly understand the strength of the evidence supporting each conclusion.

Figure S1. Alluvial plot displaying the clustering of studies based on the intervention or exposure assessed and the associated health outcomes. Each study is linked to specific interventions or exposures, which are then connected to various health outcomes.

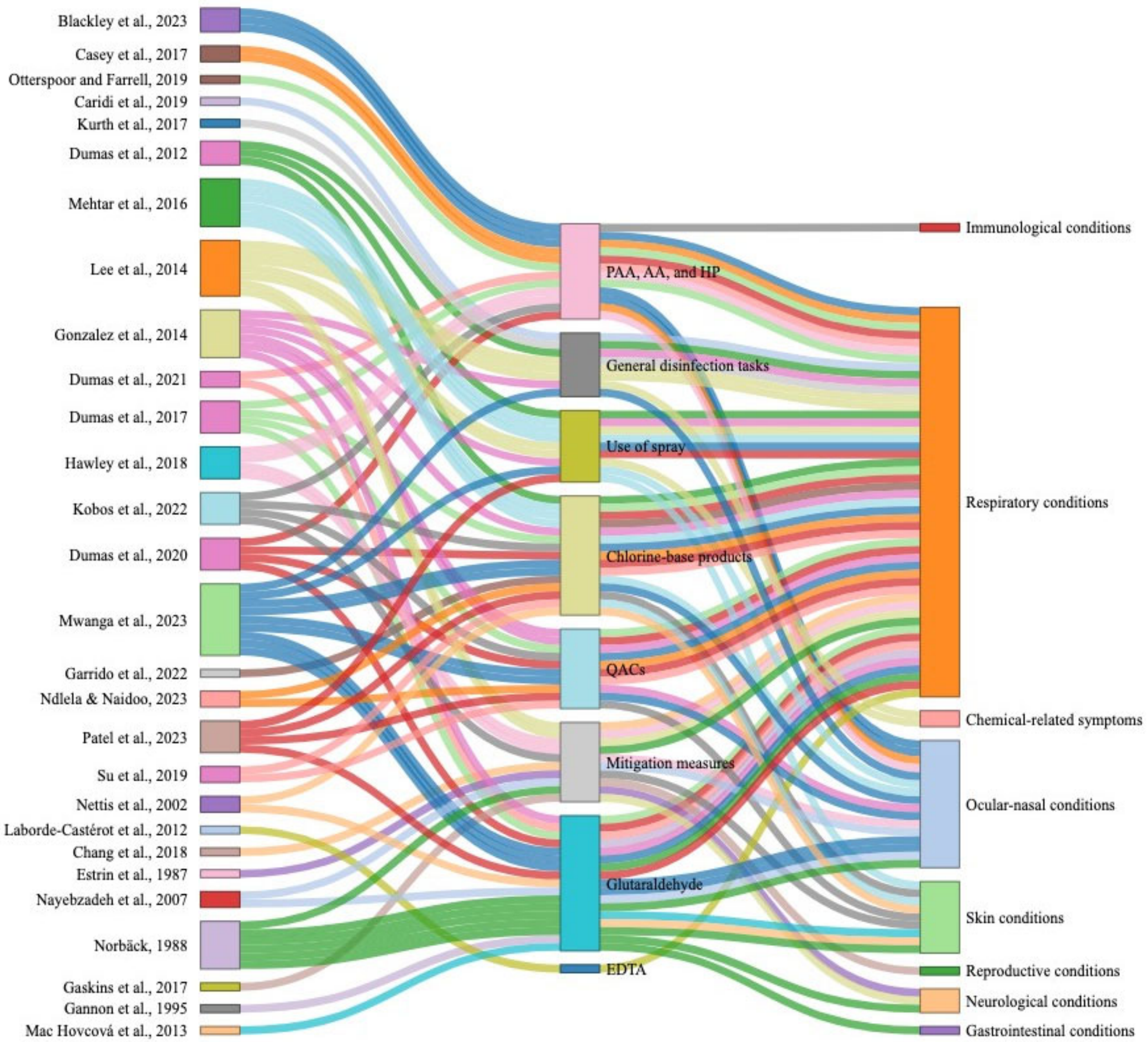

Table S1. Search strategy

Database	Search string
PubMed	1: "health personnel"[Mesh] OR ("health personnel"[tiab:~3] OR "healthcare personnel"[tiab:~3] OR "health provider"[tiab:~3] OR "health providers"[tiab:~3] OR "healthcare provider"[tiab:~3] OR "healthcare providers"[tiab:~3] OR "health worker"[tiab:~3] OR "health workers"[tiab:~3] OR "healthcare worker"[tiab:~3] OR "healthcare workers"[tiab:~3] OR "health professional"[tiab:~3] OR "health professionals"[tiab:~3] OR "healthcare professional"[tiab:~3] OR "healthcare professionals"[tiab:~3] OR "healthcare assistant"[tiab] OR "healthcare assistants"[tiab] OR "health assistant"[tiab:~3] OR "health assistants"[tiab:~3] OR paramedic*[tiab] OR nurse*[tiab] OR "Nursing Assistant"[tiab] OR "Nursing Auxiliaries"[tiab] OR "Nursing Auxiliary"[tiab] OR Anesthesiologist*[tiab] OR anesthetist*[tiab] OR Caregiver*[tiab] OR Carer*[tiab] OR "Care Givers"[tiab] OR "Care Giver"[tiab] OR "Case Manager"[tiab] OR "Case Managers"[tiab] OR Coroner*[tiab] OR "Medical Examiner"[tiab] OR "Medical Examiners"[tiab] OR surgeon*[tiab] OR "Emergency dispatcher"[tiab] OR "Emergency dispatchers"[tiab] OR "Infection Control Practitioner"[tiab] OR "Infection Control Practitioners"[tiab] OR "Laboratory Personnel"[tiab] OR "Laboratory Scientists"[tiab] OR "Laboratory Scientist"[tiab] OR "Medical Technologists"[tiab] OR "Medical Technologist"[tiab] OR "Laboratory Technicians"[tiab] OR "Laboratory Technician"[tiab] OR "Laboratory Assistants"[tiab] OR "Laboratory Assistant"[tiab] OR "Medical Staff"[tiab] OR "Medical Staffs"[tiab] OR Physician*[tiab] OR "hospital personnel"[tiab] OR doctor*[tiab] OR Pediatrician*[tiab] OR Neonatologist*[tiab] OR Pulmonologist*[tiab] OR Surgeon*[tiab] OR cleaning-worker*[tiab] OR cleaner*[tiab])<br>2: ("Disinfectants"[Mesh:noExp] OR "Disinfectants"[Pharmacological Action] OR disinfect*[tiab] OR detergents[tiab] OR sterili*[tiab] OR decontaminat*[tiab]) AND (chlorine-dioxide*[tiab] OR hypochlorite*[tiab] OR Chlorine-based[tiab] OR hypochlorous-acid*[tiab] OR bleach*[tiab] OR chlorine-product*[tiab] OR Chlorin*[tiab] OR sodium-dichloroisocyanurate*[tiab] OR Chloramine-T[tiab] OR High-level-disinfectant*[tiab] OR benzalkonium-chloride[tiab])

	3: (("Disinfectants"[Mesh:noExp] OR "Disinfectants"[Pharmacological Action] OR disinfect*[tiab] OR detergent*[tiab] OR sterili*[tiab] OR decontaminat*[tiab]) AND (spray*[tiab] OR Aerosol*[tiab] OR airway*[tiab] OR vapour* OR airborn*[tiab]))<br>4: #1 AND (#2 OR #3)<br>5: (adverse*[tw] OR safe[tw] OR safety[tw] OR side-effect*[tw] OR undesirable-effect*[tw] OR toxicity[tw] OR tolerability[tw] OR reaction*[tw] OR hazard*[tiab]) OR "Chemical Safety"[Mesh] OR "Safety Management"[Mesh] OR irritant*[tw] OR irritat*[tw] OR allerg*[tw] OR exposure*[tw] OR exposed[tw] OR increased-risk*[tw] OR health-problem*[tw])<br>6: #4 AND #5<br>7: "Letter"[Publication Type] OR "Editorial"[Publication Type] OR "comment"[Publication Type]<br>8: ("Animals"[Mesh] NOT ("Animals"[Mesh] AND "Humans"[Mesh]))<br>9: #6 not (#7 OR #8)
Scopus	1: (TITLE-ABS-KEY (((health*) w/3 (personnel OR provider* OR worker* OR professional* OR assistant*)) OR paramedic* OR nurse* OR nursing-assistant* OR nursing-auxilliar* OR Anesthesiologist* OR anesthetist* OR Caregiver* OR Carer* OR "Care Givers" OR "Care Giver" OR Case-Manager* OR Coroner* OR Medical- Examiner* OR Emergency-dispatcher* OR Infection-Control-Practitioner* OR Laboratory-Personnel OR Laboratory-Scientist* OR Medical-Technologist* OR Laboratory-Technician* OR Lab-technician* OR Laboratory-Assistant* OR Medical-Staff OR Physician* OR "hospital personnel" OR doctor* OR Pediatrician* OR Neonatologist* OR Pulmonologist* OR Surgeon* OR cleaning-worker* OR cleaner* OR ((hospital) w/5 (staff)))) AND<br>2: (TITLE-ABS-KEY (((disinfect* OR detergent* OR sterili* OR decontaminat* OR chlorine OR cleaning) AND (spray* OR aerosol* OR airway* OR vapour* OR airborn*))) OR<br>3: TITLE-ABS-KEY (((disinfect* OR detergent* OR sterili* OR decontaminat* OR (surface W/3 clean*)) AND (chlorine-dioxide* OR hypochlorite* OR chlorine-based OR hypochlorous-acid* OR bleach* OR chlorine-product* OR

	chlorin* OR sodium-dichloroisocyanurate* OR chloramine-t OR high-level-disinfectant* OR benzalkonium-chloride)))) AND<br>4: (TITLE-ABS-KEY (adverse* OR safe OR safety OR side-effect* OR undesirable-effect* OR toxicity OR tolerability OR hazard* OR irritant* OR irritat* OR allerg* OR exposure* OR exposed OR increased-risk* OR health-problem*))<br>#1 AND (#2 OR #3) AND #4 (TITLE-ABS-KEY ((((health*) W/3 (personnel OR provider* OR worker* OR professional* OR assistant*)) OR paramedic* OR nurse* OR nursing-assistant* OR nursing-auxilliar* OR anesthesiologist* OR anesthetist* OR caregiver* OR carer* OR "Care Givers" OR "Care Giver" OR case-manager* OR coroner* OR medical- AND examiner* OR emergency-dispatcher* OR infection-control-practitioner* OR laboratory-personnel OR laboratory-scientist* OR medical-technologist* OR laboratory-technician* OR lab-technician* OR laboratory-assistant* OR medical-staff OR physician* OR "hospital personnel" OR doctor* OR pediatrician* OR neonatologist* OR pulmonologist* OR surgeon* OR cleaning-worker* OR cleaner* OR ((hospital) W/5 (staff)))) AND TITLE-ABS-KEY ((((disinfect* OR detergent* OR sterili* OR decontaminat* OR chlorine OR cleaning) AND (spray* OR aerosol* OR airway* OR vapour* OR airborn*)))) OR TITLE-ABS-KEY (((disinfect* OR detergent* OR sterili* OR decontaminat* OR (surface W/3 clean*)) AND (chlorine-dioxide* OR hypochlorite* OR chlorine-based OR hypochlorous-acid* OR bleach* OR chlorine-product* OR chlorin* OR sodium-dichloroisocyanurate* OR chloramine-t OR high-level-disinfectant* OR benzalkonium-chloride))) AND TITLE-ABS-KEY ((adverse* OR safe OR safety OR side-effect* OR undesirable-effect* OR toxicity OR tolerability OR hazard* OR irritant* OR irritat* OR allerg* OR exposure* OR exposed OR increased-risk* OR health-problem*)))
Embase	1: health care personnel'/exp OR (((health*) NEAR/3 (personnel OR provider* OR worker* OR professional* OR assistant*)) OR paramedic* OR nurse* OR nursing-assistant* OR nursing-auxilliar* OR Anesthesiologist* OR anesthetist* OR Caregiver* OR Carer* OR "Care Givers" OR "Care Giver" OR Case-Manager* OR Coroner* OR Medical- Examiner* OR Emergency-dispatcher* OR Infection-Control-Practitioner* OR Laboratory-Personnel OR Laboratory-Scientist* OR Medical-Technologist* OR Laboratory-Technician* OR Lab-technician* OR Laboratory-

	Assistant* OR Medical-Staff OR Physician* OR "hospital personnel" OR doctor* OR Pediatrician* OR Neonatologist* OR Pulmonologist* OR Surgeon* OR cleaning-worker* OR cleaner* OR ((hospital) NEAR/5 (staff))):ti,ab,kw,de<br>2: ('disinfectant agent'/exp AND ('chlorine dioxide'/exp OR 'chloramine derivative'/exp OR 'hypochlorite sodium'/exp OR 'chlorine derivative'/exp)) OR ((disinfect* OR detergent* OR sterili* OR decontaminat* OR (surface NEAR/3 clean*)) AND (chlorine-dioxide* OR hypochlorite* OR Chlorine-based OR hypochlorous-acid* OR bleach* OR chlorine-product* OR Chlorin* OR sodium-dichloroisocyanurate* OR Chloramine-T OR High-level-disinfectant* OR benzalkonium-chloride)):ti,ab<br>3: ('disinfectant agent'/exp AND 'aerosol'/exp) OR ((disinfect* OR detergent* OR sterili* OR decontaminat* OR chlorine OR cleaning) AND (spray* OR aerosol* OR airway* OR vapour* OR airborn*)):ti,ab<br>4: #1 AND (#2 OR #3)<br>5: adverse event'/exp OR 'chemical safety'/exp OR 'product safety'/exp OR (adverse* OR safe OR safety OR side-effect* OR undesirable-effect* OR toxicity OR tolerability OR hazard* OR irritant* OR irritat* OR allerg* OR exposure* OR exposed OR increased-risk* OR health-problem*):ti,ab<br>6: #4 AND #5<br>7: ([animals]/lim NOT ([animals]/lim AND [humans]/lim))<br>8: [letter]/lim OR [conference abstract]/lim OR [conference paper]/lim OR [conference review]/lim OR [editorial]/lim OR [note]/lim<br>9: #6 NOT (#7 OR #8)

Table S2. Eligibility criteria

Category	Inclusion	Exclusion
Participants/population	Healthcare workers (HCWs) or workers exposed to chemical disinfectant products in occupational settings, such as hospitals, clinics, or laboratories.	HCWs or other frontline workers not exposed to chemical disinfectant products in occupational settings (e.g., administrative staff, security personnel).
Exposure(s)	- Occupational exposure to chlorine-based disinfectants.<br>- Exposure to non-chlorine-based disinfectants (e.g., hydrogen peroxide, quaternary ammonium compounds, alcohol-based disinfectants, peracetic acid, acetic acid).<br>- Comparisons between disinfectant types (chlorine-based vs. non-chlorine-based) or between disinfectants and no exposure.<br>- Studies solely assessing non-chlorine-based disinfectants or different application methods (e.g., spraying, wiping, mopping).	- Studies with no occupational exposure to disinfectants or lacking clear differentiation between disinfectant types or application methods.
Comparator(s)/control	- HCWs exposed to chlorine-based disinfectants versus non-chlorine-based disinfectants.<br>- HCWs exposed to disinfectants versus no disinfectants.<br>- HCWs exposed to different application methods (e.g., spraying, wiping, mopping).	- Groups with elevated risks due to non-occupational exposures (e.g., smoking, pollution).<br>- Studies not reporting clear comparator groups or exposure details.

Outcome	- Respiratory: asthma, COPD, or other obstructive diseases.<br>- Skin: dermatitis, burning, allergic reactions.<br>- Eye: pain, irritation, vision issues.<br>- Reproductive: pregnancy-related outcomes (e.g., preterm birth, pregnancy loss).<br>- Exposure markers: airborne gas levels.	- Medical conditions not linked to disinfectant exposure (e.g., unrelated chronic illnesses).<br>- Outcomes not linked to occupational exposure or disinfection practices.
Study design	Case reports, case series, cohort studies, case-control studies, cross-sectional studies, experimental studies, and observational studies.	Qualitative studies, studies with only abstracts, conference papers/posters, reviews, letters, editorials.

Table S3. Data extraction template for included studies

**Authors, Year, study design**	Risk of bias	Study objective	Type of recruitment, population	Sex, age	Sample size	Exposure (category), assessment	Outcome (cluster) assessment	Adjustment confounding
Authors, year of publication, Type of study conducted	Results of bias assessment	Main aim or objective of the study	How participants were recruited for the study (e.g., random sampling, convenience sampling, volunteers from a specific population).	The demographics of the participants, including the percentage of male and female participants and their age range or mean age.	The total number of participants in the study.	The methods used to measure or assess the exposure of interest (e.g., self-reported questionnaires, direct measurements, use of specific equipment).	The methods used to measure the outcomes of interest (e.g., clinical evaluations, laboratory tests, surveys).	Any confounding variables that the study adjusted for in its analysis (e.g., age, sex, smoking status, socioeconomic status).

Table S4. Risk of bias instrument

Major risk of bias domains	Risk	Criteria
1. Recruitment procedure & follow-up (in cohort studies):<br><br>For cohort studies<br>HINT: We are looking for selection bias:<br>- Was the cohort representative of a defined population? #<br>- Was everybody included who should have been included? #<br><br>PRELIMINARY RULING: If the cohort recruitment is based on a convenient/ self-reported sampling OR if response is <10%, the study will be excluded from analysis.	Low	▪ Cohort recruitment was acceptable. #<br>▪ Baseline response level is acceptable (50% or more) OR is <50% and >30%, but substantial differential selection could be excluded (e. g. by a non-responder analysis).<br>▪ Loss to follow-up is below 20% in total and not different between the two groups (up to 10% difference). *
	High	▪ Cohort recruitment was not acceptable. #<br>▪ Response not reported/ not calculable.<br>▪ Total loss to follow-up is larger than acceptable (20% or more) * OR drop out differs between the groups by more than 10%* OR the reasons for drop out considerably differ between exposed and non-exposed groups. *
For case-control studies<br><br>HINT: We are looking for selection bias:<br>- Were the cases and control subjects representative of the same defined population ("study base"; geographically and/or temporally)? #	Low	▪ Case selection and recruitment were acceptable. #<br>▪ Control subjects' selection and recruitment were acceptable. #<br>▪ Non-response was less than 50% for cases and/or control subjects OR it was >50% and <70%, but substantial differential selection of cases and control subjects could be excluded (e.g., by a non-responder analysis) *

- Was there an established reliable system for selecting all the cases? #<br>- The same exclusion criteria are used for both cases and controls. #<br>- Comparison is made between participants and non-participants to establish their similarities or differences. #<br><br>PRELIMINARY RULING: If the recruitment is based on a convenient/ self-reported sampling OR if response is <10%, the study will be excluded from analysis.	High	▪ Case selection and recruitment were not acceptable. #<br>▪ Control subjects' selection and recruitment were not acceptable. #<br>▪ Non-response was >70% for cases or control subjects OR it was >50% and<70%, but substantial differential selection of cases and control subjects could not be excluded. *<br>▪ Response not reported/ not calculable
For cross-sectional studies<br><br>HINT: We are looking for selection bias:<br>- Was the study population representative of a defined population? #<br>- Was everybody included who should have been included? #<br><br>PRELIMINARY RULING: If the recruitment is based on a convenient/ self-reported sampling OR if response is <10%, the study will be excluded from analysis.	Low	▪ Recruitment of the study population was acceptable. #<br>▪ Non-response was less than 50% OR it was >50% and <70%, but substantial differential selection of the study population could be excluded (e.g., by a non-responder analysis). *
	High	▪ Recruitment of the study population was not acceptable. #<br>▪ Non-response was >70% OR it was >50% and <70%, but substantial differential selection of the<br>▪ study population could not be excluded. *<br>▪ Response not reported/ not calculable.
2. Exposure definition and measurement	Low	▪ Exposure definition included at least basic job characteristics (e.g., job tasks, length of employment).

		▪ Exposure was accurately measured to minimize bias: use of external validation, validated questionnaire or exposure is connected to a task) # ▪ Adequate comparison group of non-exposed workers (e.g., office workers) included.
	High	▪ Exposure does not cover basic job characteristics. ▪ Exposure was not accurately measured (e.g., use of JEM only) # ▪ Different methods were used to measure exposure in different groups/ cases and control subjects (in case-control studies). ▪ No adequate comparison group of non-exposed workers included
	Unclear	▪ Not reported
3. Outcome, Source and validation	Low	▪ Outcome was accurately/ objectively measured to minimize bias ▪ Measurement methods were similar in the different groups. #
	High	▪ Outcome was not accurately or subjectively measured (e.g., self-reported physician diagnosis or questionnaire). # ▪ Measurement methods were different in the groups.
	Unclear	▪ Not reported

4. Confounding and effect modification	Low	▪ If risk estimators were calculated, major confounding factors (at least age, sex, atopy) were considered.<br>▪ If only prevalence or incidence was assessed, at least sex & age (at least mean values for the study population) are described.
	High	▪ Major confounding factors or effect modifiers were not considered.
	Unclear	▪ Not reported
5. Analysis method: methods to reduce research specific bias	Low	▪ Authors used adequate statistical models to reduce bias (e.g., standardization, matching, adjustment in multivariate model, stratification, propensity scoring). § For prevalences, matching/stratification may not be required as long as a good description of the age structure and immunization status of the population is given.
	High	▪ Authors did not use adequate statistical models to reduce bias.
	Unclear	▪ Not reported
6. Chronology	Low	▪ Incident diseases were included. #<br>▪ Temporal relation may be established (exposure precedes the outcome). #
	High	▪ Prevalent diseases were included OR prevalent diseases of baseline were not excluded (in cohort studies). #<br>▪ Temporal relation cannot be established.
	Unclear	▪ Not reported

Minor risk of bias domains	Risk	Criteria
7. Blinding of assessors	Low	▪ Assessors were blinded
	High	▪ Assessors were not blinded
	Unclear	▪ Not reported
8. Funding	Low	▪ Grant/ non-profit-organizations*<br>▪ Study was clearly not affected by sponsors. *
	High	▪ Sponsoring organization participated in data analysis.<br>▪ Study was probably affected by sponsors.
	Unclear	▪ Industry, combined industry grant*, unclear if study was affected by<br>▪ sponsors.<br>▪ Not reported.
9. Conflict of interest	Low	▪ Reported not having conflict of interest or clear from report/ communication that study was not affected by author(s) affiliation. *
	High	▪ Conflict of interest exists (at least one author). *
	Unclear	▪ Not reported.

Overall Assessment<br>General rule for rating	Low	▪ low risk in all major domains

Table S5. Quantitative data extracted from included studies

Chlorine-base products						
**Study**	**Specific product**	**Health outcome**	**Health condition clusters**	**OR (95% CI)**	**p-value**	**Sample size**
Dumas et al., 2017	Bleach	Poorer asthma control	Respiratory	1.55 (1.14-2.1)	0.02	1884
Dumas et al., 2012	Bleach			2.11 (0.88-5.03)	>0.05	212
Gonzalez et al., 2014	Bleach	New-onset asthma	Respiratory	2.08 (0.86-5.0)	0.1	543
Garrido et al., 2022	Bleach	Respiratory symptoms	Respiratory	2.46 (1.01-6.89)	>0.05	307
Kobos et al., 2022	Bleach	Skin Disorder	Skin	1.79 (1.14-2.8)	0.05	559
Mehtar et al., 2016	Chlorine 0,05%	Respiratory conditions	Respiratory	32.95 (22.0-49.0)	<0.001	1550
Mehtar et al., 2016	Chlorine 0,05%	Eye conditions	Ocular-nasal	30.95 (21.0-43.0)	<0.001	1550
Mehtar et al., 2016	Chlorine 0,05%	Skin conditions	Skin	22.95 (15.0-32.0)	<0.001	1550
Mwanga et al., 2023	Bleach	WRONS*	Ocular-nasal	1.12 (0.7-1.78)	>0.05	697
Mwanga et al., 2023	Bleach	WRONS**	Ocular-nasal	0.69 (0.34-1.42)	>0.05	697
Mwanga et al., 2023	Bleach	WRONS***	Ocular-nasal	2.37 (1.3-4.34)	<0.001	697
Mwanga et al., 2023	Bleach	Work-related Asthma*	Respiratory	0.65 (0.32-1.33)	>0.05	697
Mwanga et al., 2023	Bleach	Work-related Asthma**	Respiratory	0.24 (0.06-1.03)	>0.05	697
Mwanga et al., 2023	Bleach	Work-related Asthma***	Respiratory	1.16 (0.49-2.75)	>0.05	697
Ndlela & Naidoo, 2023	Chlorine	Breathless with Wheeze	Respiratory	1.26 (0.45-3.46)	>0.05	174
Ndlela & Naidoo, 2023	Chlorine	Wheeze without cold	Respiratory	1.48 (0.59-3.68)	>0.05	174
Ndlela & Naidoo, 2023	Chlorine	Shortness of Breath	Respiratory	1.27 (0.48-3.21)	>0.05	174

Ndlela & Naidoo, 2023	Chlorine	Chronic Cough	Respiratory	1.13 (0.45-2.08)	>0.05	174
Ndlela & Naidoo, 2023	Chlorine	Chronic Phlegm	Respiratory	0.64 (0.19-2.14)	>0.05	174
Ndlela & Naidoo, 2023	Chlorine	Breathlessness	Respiratory	1.04 (0.41-2.64)	>0.05	174
Ndlela & Naidoo, 2023	Bleach	Breathless with Wheeze	Respiratory	1.39 (0.47-4.08)	>0.05	174
Ndlela & Naidoo, 2023	Bleach	Wheeze without cold	Respiratory	0.8 (0.31-2.05)	>0.05	174
Ndlela & Naidoo, 2023	Bleach	Shortness of Breath	Respiratory	1.68 (0.55-5.14)	>0.05	174
Ndlela & Naidoo, 2023	Bleach	Chronic Cough	Respiratory	1.5 (0.61-3.65)	>0.05	174
Ndlela & Naidoo, 2023	Bleach	Chronic Phlegm	Respiratory	0.34 (0.97-1.2)	>0.05	174
Ndlela & Naidoo, 2023	Bleach	Breathlessness	Respiratory	1.33 (0.48-3.68)	>0.05	174
Su et al., 2019	Chlorine	U-Asthma	Respiratory	3.11 (1.46-6.63)	0.003	885
Su et al., 2019	Chlorine	Asthma exacerbations	Respiratory	2.71 (1.25-5.86)	0.011	885
Patel et al, 2023	Bleach	New asthma onset	Respiratory	1.91 (1.10-3.33)	<0.05	2,421
**Glutaraldehyde**						
**Study**	**Specific product**	**Health outcome**	**Health outcome cluster**	**OR (95% CI)**	**p-value**	**Sample size**
Gonzalez et al., 2014	GU	New-onset asthma	Respiratory	3.01 (0.92-9.86)	0.06	248
Dumas et al., 2017	GU	Poorer asthma control	Respiratory	1.54 (1.15-2.06)	0.02	2193
Norbäck, 1988	GU	Nasal catarrh	Ocular-nasal	3.0 (NA-NA)	0.04	107
Dumas et al., 2020	GU	Asthma incidence	Respiratory	(HR) 1.11 (0.88-1.41)	>0.05	35,665
Dumas et al., 2021	GU	Asthma incidence	Respiratory	(HR) 1.61 (1.00-2.59)	>0.05	1,869
Norbäck, 1988	GU	Nasal obstruction	Respiratory	2.9 (NA-NA)	0.03	107

Norbäck, 1988	GU	Smarting of the throat	Respiratory	3.6 (NA-NA)	0.02	107
Norbäck, 1988	GU	Headache	Neurological	2.6 (NA-NA)	0.04	107
Norbäck, 1988	GU	Nausea	Gastrointestinal	4.9 (NA-NA)	0.05	107
Norbäck, 1988	GU	Rashes on the hands	Skin	4.4 (NA-NA)	0.0009	107
Norbäck, 1988	GU	Eczema	Skin	6.2 (NA-NA)	0.002	107
Mwanga et al., 2023	GU	WRONS*	Ocular-nasal	1.2 (0.56-2.57)	>0.05	697
Mwanga et al., 2023	GU	WRONS**	Ocular-nasal	0.57 (0.17-1.95)	>0.05	697
Mwanga et al., 2023	GU	WRONS***	Ocular-nasal	3.69 (1.3-10.45)	<0.05	697
Mwanga et al., 2023	GU	Work-related Asthma*	Respiratory	0.88 (0.29-2.66)	>0.05	697
Mwanga et al., 2023	GU	Work-related Asthma**	Respiratory	0.79 (0.18-3.57)	>0.05	697
Mwanga et al., 2023	GU	Work-related Asthma***	Respiratory	1.45 (0.3-6.95)	>0.05	697
Patel et al., 2023	GU	New onset asthma	Respiratory	1.24 (0.78-1.95)	>0.05	2,421
**Quaternary Ammonium Compounds**						
**Study**	**Specific product**	**Health outcome**	**Health outcome cluster**	**OR (95% CI)**	**p-value**	**Sample size**
Gonzalez et al., 2014	QACs	New asthma	Respiratory	7.56 (1.84-31.05)	0.005	543
Gonzalez et al., 2014	QACs	Nasal symptoms	Ocular-nasal	3.21 (1.42-7.22)	0.005	543
Dumas et al., 2017	QACs	Poorer asthma control	Respiratory	1.31 (0.97-1.75)	0.14	4102
Kobos et al., 2022	QACs	Skin Disorders	Skin	2.49 (1.25-4.94)	0.05	559
Su et al., 2019	QACs	Mild asthma	Respiratory	0.64 (0.35-1.19)	NA	2030
Su et al., 2019	QACs	U-Asthma	Respiratory	1.29 (0.47-3.49)	NA	2030
Su et al., 2019	QACs	Asthma exacerbations	Respiratory	1.37	NA	2030

				(0.48-3.9)		
Ndlela & Naidoo, 2023	QACs	Breathless with Wheeze	Respiratory	0.95 (0.33-2.73)	>0.05	174
Ndlela & Naidoo, 2023	QACs	Wheeze without cold	Respiratory	1.28 (0.49-3.33)	>0.05	174
Ndlela & Naidoo, 2023	QACs	Shortness of Breath	Respiratory	3.44 (1.13-10.51)	<0.05	174
Ndlela & Naidoo, 2023	QACs	Chronic Cough	Respiratory	0.69 (0.26-1.82)	>0.05	174
Ndlela & Naidoo, 2023	QACs	Chronic Phlegm	Respiratory	0.59 (0.18-1.91)	>0.05	174
Ndlela & Naidoo, 2023	QACs	Breathlessness	Respiratory	1.72 (0.66-4.47)	>0.05	174
Mwanga et al., 2023	QACs	WRONS	Ocular-nasal	1.04 (0.33-3.26)	>0.05	697
Mwanga et al., 2023	QACs	Work-related Asthma	Respiratory	2.7 (0.71-10.23)	>0.05	697
Patel et al, 2023	QACs	New asthma onset	Respiratory	1.91 (1.10-3.33)	<0.05	2,421
**Peracetic acid, acetic acid and hydrogen peroxide**						
**Study**	**Specific product**	**Health outcome**	**Health outcome cluster**	**OR (95% CI)**	**p-value**	**Sample size**
Otterspoor and Farrell, 2019	PAA	increased safe-work related incident reporting		NA	NA	64
Casey et al., 2017	PAA, AA, and HP	watery eyes	Ocular-nasal	2.88 (NA-NA)	>0.05	163
Kobos et al., 2022	HP	Allergic Reactions	Immunological	6.12 (1.18-44.47)	0.05	559
Hawley et al., 2018	PAA, AA, and HP	eye (44%), upper airway (58%), and lower airway (34%) symptoms				50
Blackley et al., 2023	PAA, AA, and HP	Nasal irritation	Ocular-nasal	1.91 (1.12-3.23)	NA	67
Blackley et al., 2023	PAA, AA, and HP	Eye irritation	Ocular-nasal	1.72 (1.14-2.6)	NA	67
**Use of spraying**						
**Study**	**Exposure**	**Health outcome**	**Health outcome cluster**	**OR (95% CI)**	**p-value**	**Sample size**
Lee et al., 2014	high exposure	SRCE	CRS	1.79 (0.81-3.96)	>0.05	183
Lee et al., 2014	high exposure	Respiratory symptoms	Respiratory	1.98	>0.05	183

				(0.87-4.51)		
Lee et al., 2014	medium exposure	SRCE	CRS	2.82 (1.16-6.82)	<0.05	183
Lee et al., 2014	NA	Respiratory symptoms	Respiratory	3.16 (1.24-8.04)	<0.05	18
Mwanga et al., 2023	medium exposure	HASS	Respiratory	5.01 (1.8-13.91)	<0.01	697
Dumas et al., 2012	NA	Current asthma	Respiratory	2.87 (1.02-8.11)	<0.05	724
Mehtar et al., 2016	NA	Respiratory conditions	Respiratory	32.95 (22-49)	<0.001	1550
Mehtar et al., 2016	NA	Eye conditions	Ocular-nasal	30.95 (21-43)	<0.001	1550
Mehtar et al., 2016	NA	Skin conditions	Skin conditions	22.95 (15-32)	<0.001	1550
Gonzalez et al., 2014	NA	New-onset asthma	Respiratory	1.3 (0.56-3.04)	0.53	543
Patel et al, 2023		New asthma onset	Respiratory	1.92 (1.12-3.47)	<0.05	2,421

General disinfection tasks

Study	tasks	Exposure	Health outcome	Health outcome cluster	OR (95% CI)	p-value	Sample size
Caridi et al., 2019	Clean Fixed Surfaces	NA	moderate exacerbation	Respiratory	3.10 (1.25-7.67)	<0.05	2,030
Caridi et al., 2019	Clean Fixed Surfaces	NA	Current asthma	Respiratory	1.84 (1.26, 2.68)	<0.05	2,030
Caridi et al., 2019	Clean Fixed Surfaces	NA	bronchial hyper-responsiveness-related symptoms	Respiratory	1.38 (1.08-1.77)	<0.05	2,030
Gonzalez et al., 2014	general disinfection	NA	New-onset asthma	Respiratory	4.68 (1.08-20.22)	0.03	543
Gonzalez et al., 2014	dilution of disinfectants	NA	New-onset asthma	Respiratory	4.56 (1-20.29)	0.049	543

Dumas et al., 2012	cleaning/disinfecting tasks	moderate/high intensity	Current asthma	Respiratory	2.32 (1.11-4.86)	<0.05	724
Lee et al., 2014	Mopping, wet cleaning, and damp wiping	medium exposure	SRCE	CRS	1.36 (0.52-3.61)	>0.05	183
Lee et al., 2014	Mopping, wet cleaning, and damp wiping	high exposure	SRCE	CRS	1.62 (0.58-4.55)	>0.05	183
Lee et al., 2014	Mopping, wet cleaning, and damp wiping	medium exposure	Respiratory symptoms	Respiratory	2.3 (0.74-7.17)	>0.05	183
Lee et al., 2014	Mopping, wet cleaning, and damp wiping	high exposure	Respiratory symptoms	Respiratory	3.11 (0.94-10.3)	>0.05	183
Mwanga et al., 2023	Manually sterilise/disinfection medical instruments	≥100 min/week	WRONS	Ocular-nasal	2.92 (1.33-6.41)	<0.01	37

OR = Odds ratio, 95% CI = 95% confidence interval, WRONS = Work-related ocular-nasal symptoms, * = Exposed versus not exposed, **= exposed 1–99 min/week, ***= exposed ≥100 min/week, U-Asthma = Undiagnosed/untreated asthma, GU = glutaraldehyde, CH = Cohort study, CR = Cross-sectional study, CA = Case control study, CS = Case study, SRCE = Symptoms related to chemical exposure, CRS= Chemical-related symptoms, HASS = Higher Asthma Symptom Score, NA = Not applicable, HR = hazard rate

Table S6. Excluded studies and reasons for exclusion

Reference<br>(o = outcome, i = intervention, p = population, d = design, b = background article, t -= publication type, l = language, u = Update available, r = only research protocol, n = reports not retrieved	Reasons for exclusion									
	o	i	p	d	b	t	l	u	r	n
(L. AD et al., 1993)			X							
(T. AD et al., 1991)			X							
(Afework et al., 2023)		X								
(Agathos & HA, 1982)				X						
(Agthe et al., 2009)		X								
(Ahmed-Lecheheb et al., 2012)		X								
(Alexandre et al., 2021)		X								
(Alexis DESCATHA Jean-Baptiste BARBE-RICHAUD, Jeremy BLOTTIAUX, 2022)		X								
(Alimohammadi & Naderi, 2021)				X						
(Alnimr et al., 2021)	X									
(Ambrosino et al., 2022)	X									
(Amini Tapouk et al., 2020)	X									
(Amlôt et al., 2017)			X							
(Amr & Bollinger, 2004)		X								
(Angelova-Fischer et al., 2016)	X									
(Anggraini & Lestari, 2022)	X									
(Antolín et al., 2021)			X							
(Archangelidi et al., 2021)				X						
(Assadian et al., 2021)			X							
(Astrid et al., 2021)	X									
(Azmi et al., 2021)		X								
(Babić et al., 2020)			X							
(Baccolini et al., 2019)		X								
(Barbaud, 2002)			X							
(Bardaweel et al., 2023)		X								
(Batalla et al., 2012)							X			
(Bauer et al., 2023)			X							
(Bédard et al., 2014)			X							
(Berger et al., 2022)	X									

Reference (o = outcome, i = intervention, p = population, d = design, b = background article, t -= publication type, l = language, u = Update available, r = only research protocol, n = reports not retrieved	Reasons for exclusion									
	o	i	p	d	b	t	l	u	r	n
(Bernardini et al., 1983)										X
(Bessonneau et al., 2013)		X								
(Bhat et al., 2022)				X						
(Bijlsma & Cohen, 2016)						X				
(Brune et al., 2021)	X									
(Burgaz et al., 1992)	X									
(Byrns et al., 2017)	X									
(Caetano et al., 2021)	X									
(Carling, 2021)				X						
(Carling et al., 2023)	X									
(Carol Sharma et al., 2023)			X							
(Carvalho & Conte-Junior, 2021)		X								
(CC et al., 2011)			X							
(Cebeci et al., 2021)		X								
(Centers for Diseases Prevention and Control, 1984)										X
(Chaari et al., 2010)					X					
(Charlier et al., 2021)				X						
(Choi et al., 2021)				X						
(CL et al., 2016)		X								
(Clausen et al., 2020)				X						
(Cochrane et al., 2015)				X						
(Coggon et al., 2004)			X							
(CP et al., 2005)					X					
(Cucurachi & MG, 2010)			X							
(Curran et al., 2019)				X						
(Cutts et al., 2021)	X									
(Cutuli et al., 2021)		X								
(CY et al., 2003)							X			
(Daba et al., 2022)		X								
(Davidovits et al., 2003)			X							

(Dalton et al., 2023)			X							
Reference<br>(o = outcome, i = intervention, p = population, d = design, b = background article, t -= publication type, l = language, u = Update available, r = only research protocol, n = reports not retrieved	Reasons for exclusion									
	o	i	p	d	b	t	l	u	r	n
(De Groot et al., 2021)		X								
(de Oliveira et al., 2023)	X									
(De Troeyer et al., 2022)			X							
(Delclos et al., 2007)		X								
(Descatha et al., 2023)		X								
(Dhama et al., 2021)			X							
(DJ et al., 1993)		X								
(Ding et al., 2021)		X								
(Doll et al., 2018)										
(Donnay et al., 2011)				X						
(Doolan & Crilly, 2019)		X								
(Dore et al., 2022)			X							
(Dorgham & Dorgham, 2021)				X						
(Dotson et al., 2020)	X									
(Dumas, 2021)								X		
(Dumas, Bédard, et al., 2021)	X									
(Dumas & Le Moual, 2020)				X						
(Dumas et al., 2014)	X									
(Dumas et al., 2015)	X									
(Dumas, Wiley, Henneberger, et al., 2017)		X								
(EL et al., 2020)		X								
(Epelle et al., 2023)				X						
(Erdem et al., 2020)		X								
(Erfani et al., 2023)				X						
(Estienney et al., 2022)	X									
(F et al., 1998)										X
(Faller et al., 2018)		X								
(Felemban et al., 2021)		X								
(Fickenscher et al., 2023)	X									

(Flyvholm, 1993)			X							
(Folletti et al., 2017)			X							
Reference (o = outcome, i = intervention, p = population, d = design, b = background article, t -= publication type, l = language, u = Update available, r = only research protocol, n = reports not retrieved	Reasons for exclusion									
	o	i	p	d	b	t	l	u	r	n
(França et al., 2019)		X								
(Frickmann et al., 2018)		X								
(García-Ávila et al., 2020)			X							
(Garvey et al., 2018)	X									
(Gasparini et al., 2020)		X								
(Ge et al., 2021)	X									
(Gerding et al., 2023)			X							
(Gerster et al., 2014)			X							
(Ghafoor et al., 2021)			X							
(Gharaibeh et al., 2021)	X									
(Ghelli et al., 2022)		X								
(Goodyear et al., 2018)	X									
(Gould, 1994)		X								
(Graham et al., 2023)			X							
(Groenewold et al., 2019)			X							
(Gruszecka et al., 2021)	X									
(GS, 1988)				X						
(Gupta et al., 2021)	X									
(Gutterman et al., 2013)		X								
(HA, 1992)		X								
(Hamnerius et al., 2021)			X							
(Hamnerius, Svedman, Bergendorff, Björk, Bruze, Engfeldt, et al., 2018)			X							
(Hamnerius, Svedman, Bergendorff, Björk, Bruze, & Pontén, 2018)		X								
(A. A. Han et al., 2022)		X								
(J. H. Han et al., 2015)				X						
(Hatt et al., 2020)	X									
(Haufroid et al., 2007)	X									
(Hautemanière et al., 2013)	X									

(Hawley et al., 2016)				X						
(Heibati et al., 2022)			X							
Reference<br>(o = outcome, i = intervention, p = population, d = design, b = background article, t -= publication type, l = language, u = Update available, r = only research protocol, n = reports not retrieved	Reasons for exclusion									
	o	i	p	d	b	t	l	u	r	n
(Held et al., 2002)			X							
(Hell & Pauser, 2007)				X						
(Henn et al., 2015)	X									
(Hetzmann et al., 2021)		X								
(HG et al., 2023)			X							
(HH, 1971)										X
(HH et al., 2021)			X							
(Hildre et al., 2023)			X							
(HK et al., 2023)		X								
(Holtjer et al., 2023)			X							
(Homyer & Mehendale, 2023)	X									
(Houben et al., 2006)		X								
(Huang et al., 2023)	X									
(IA et al., 1995)			X							
(Ilyas et al., 2020)				X						
(Ishihara et al., 2020)	X									
(Ituen et al., 2021)				X						
(JA et al., 2021)			X							
(Jacobsen et al., 2022)			X							
(Jann et al., 2021)	X									
(JD et al., 2008)			X							
(JE et al., 1990)			X							
(L. JM et al., 2022)	X									
(M. JM & DN, 2016)		X								
(Jonsdottir et al., 2023)			X							
(JP et al., 1984)	X									
(JS et al., 2020)	X									
(JW et al., 1983)										X

(KA et al., 2021)	X									
(Kampf, 2008)				X						
Reference<br>(o = outcome, i = intervention, p = population, d = design, b = background article, t -= publication type, l = language, u = Update available, r = only research protocol, n = reports not retrieved	Reasons for exclusion									
	o	i	p	d	b	t	l	u	r	n
(Kampf & Muscatiello, 2003)		X								
(Kampf et al., 2002)		X								
(Kampf et al., 2008)		X								
(Kampf et al., 2020)	X									
(Kathare et al., 2022)			X							
(Keegel & RL, 2018)			X							
(Kieć-Swierczyńska, 1996)			X							
(Kieć-Swierczyńska & Krecisz, 2000)										X
(Kiely et al., 2021)		X								
(Kirman et al., 2021)				X						
(C. KM, 2015)		X								
(O. KM & Farooque, 2014)				X						
(Konno et al., 2023)		X								
(Kovach et al., 2017)	X									
(Krause & Dolák, 2021)	X									
(Kruszewska et al., 2022)			X							
(KS, GB, & Agner, 2012)			X							
(KS et al., 2016)		X								
(KS, GB, TL, et al., 2012)		X								
(Kundrapu et al., 2012)		X								
(Laditka et al., 2020)			X							
(Larese Filon et al., 2021)				X						
(Larner et al., 2020)	X									
(S. Lee et al., 2021)		X								
(Lei et al., 2017)	X									
(Leinster et al., 1993)	X									
(Lemire et al., 2022)			X							
(Li et al., 2018)			X							

(Lin et al., 2021)			X							
(Liss et al., 2003)		X								
(Lodola et al., 2000)		X								
Reference<br>(o = outcome, i = intervention, p = population, d = design, b = background article, t -= publication type, l = language, u = Update available, r = only research protocol, n = reports not retrieved	Reasons for exclusion									
	o	i	p	d	b	t	l	u	r	n
(Lu et al., 2022)	X									
(Lu et al., 2023)	X									
(Luchini et al., 2021)			X							
(da S. MA et al., 1997)	X									
(G. J. MA et al., 2013)									X	
(Maier et al., 2015)	X									
(Małaszuk et al., 2000)										X
(Mallakpour et al., 2021)		X								
(Manyele et al., 2008)		X								
(Marena et al., 2002)	X									
(Matulonga et al., 2016)			X							
(McKinley et al., 2023)	X									
(Melgar et al., 2022)	X									
(Mirabelli et al., 2007)	X									
(Moccia et al., 2020)	X									
(Mohite et al., 2022)		X								
(Mohtar et al., 2021)	X									
(Molin et al., 2015)		X								
(MS et al., 2006)	X									
(Mungan et al., 2019)			X							
(Munyendo & Kiprop, 2016)		X								
(MV et al., 2008)			X							
(Mwanga et al., 2022)	X									
(Nakamura et al., 2020)	X									
(Navarathna et al., 2023)		X								
(Nemli et al., 2021)				X						
(Neves et al., 2021)		X								

(Nguyen et al., 2021)				X						
(Nielsen & Bach, 1999)			X							
(Obed et al., 2021)		X								
Reference<br>(o = outcome, i = intervention, p = population, d = design, b = background article, t -= publication type, l = language, u = Update available, r = only research protocol, n = reports not retrieved	Reasons for exclusion									
	o	i	p	d	b	t	l	u	r	n
(Ofstead et al., 2022)	X									
(Oie et al., 2011)	X									
(Okazaki et al., 2022)			X							
(Okeke et al., 2023)				X						
(Omrane et al., 2022)			X							
(Ortí-Lucas & Muñoz-Miguel, 2017)	X									
(Oza et al., 2022)		X								
(PA et al., 1992)	X									
(Pałczyński et al., 2001)			X							
(Parry et al., 2022)	X									
(PE et al., 1990)					X					
(Pechter et al., 2005)			X							
(Pemberton & Kimber, 2021)						X				
(Peters et al., 2022)		X								
(Petti et al., 2013)	X									
(Peyneau et al., 2022)				X						
(Pironti et al., 2021)	X									
(Polecka et al., 2023)			X							
(Polivka et al., 2022)				X						
(Pontes et al., 2008)			X							
(Popin et al., 2008)			X							
(Popp et al., 1994)	X									
(Prodi, Rui, AB, et al., 2016)		X								
(Prodi, Rui, Fortina, et al., 2016)		X								
(Purwar et al., 2022)				X						
(Quinn et al., 2015)				X						
(Quinot et al., 2018)		X								

(Quinot et al., 2017)		X								
(Rai et al., 2020a)	X									
(Rai et al., 2020b)		X								
Reference<br>(o = outcome, i = intervention, p = population, d = design, b = background article, t -= publication type, l = language, u = Update available, r = only research protocol, n = reports not retrieved	Reasons for exclusion									
	o	i	p	d	b	t	l	u	r	n
(Rangel et al., 2022)	X									
(Rava et al., 2017)			X							
(Razali et al., 2022)				X						
(Restrepo et al., 2014)	X									
(Ricke et al., 2022)			X							
(Rideout et al., 2005)										X
(Rivers et al., 2021)			X							
(Roberts et al., 2022)			X							
(Robinson et al., 2019)	X									
(Rock et al., 2022)	X									
(Rogers et al., 2023)		X								
(Rollins et al., 2020)						X				
(Rose et al., 2019)	X									
(Rustemeyer et al., 1994)			X							
(Rutala & Weber, 2019)				X						
(Rutala & Weber, 2016)				X						
(Rybka et al., 2021)	X									
(Saito et al., 2015)	X									
(Salomone et al., 2018)	X									
(Sanguinet & Edmiston, 2021)	X									
(Santovito et al., 2014)		X								
(Sattar et al., 2015)	X									
(Sauvé & Friesen, 2019)	X									
(Schäferhenrich et al., 2023)			X							
(Scheepers et al., 2017)		X								
(Schnell et al., 2021)		X								
(Schnuch et al., 1998)				X						

(Schöbel et al., 2023)	X									
(Schulte et al., 2012)		X								
(Sedeh et al., 2023)		X								
Reference<br>(o = outcome, i = intervention, p = population, d = design, b = background article, t -= publication type, l = language, u = Update available, r = only research protocol, n = reports not retrieved	Reasons for exclusion									
	o	i	p	d	b	t	l	u	r	n
(Seifi & Reza Kamali, 2021)	X									
(Sellaoui et al., 2021)	X									
(Shepherd et al., 2020)		X								
(Sher & Mulder, 2020)	X									
(Shi et al., 2022)		X								
(Si et al., 2018)	X									
(Siani & Maillard, 2015)				X						
(Siani et al., 2018)	X									
(S. E. Simmons et al., 2021)	X									
(S. Simmons et al., 2021)	X									
(Singgih et al., 1986)		X								
(Sit et al., 2021)			X							
(Sit et al., 2022)		X								
(SJ et al., 2015)		X								
(SJ et al., 2013)			X							
(Society of Gastroenterology Nurses and Associates, 1996)					X					
(Sonday et al., 2023)			X							
(Song et al., 2019)				X						
(Spencer et al., 2017)		X								
(Starke et al., 2021)				X						
(Steege et al., 2014)			X							
(Stjärne Aspelund et al., 2016)	X									
(Stoeva et al., 2020)		X								
(F.-C. Su et al., 2018)	X									
(Takigawa & Endo, 2006)				X						
(Tang et al., 2023)	X									
(Tasar et al., 2021)			X							

(Teska et al., 2022)	X									
(TL, 1987)					X					
(Tofanelli et al., 2020)	X									
(Tomb et al., 2018)	X									
Reference (o = outcome, i = intervention, p = population, d = design, b = background article, t -= publication type, l = language, u = Update available, r = only research protocol, n = reports not retrieved	Reasons for exclusion									
	o	i	p	d	b	t	l	u	r	n
(Tyski et al., 2022)				X						
(Tyski et al., 2021)	X									
(Vaidya et al., 2020)		X								
(van den Berg et al., 2021)		X								
(VC et al., 2003)		X								
(Versoza et al., 2020)	X									
(Vincent et al., 2017)	X									
(Vizcaya et al., 2013)		X								
(Walton & Rogers, 2017)				X						
(Watanabe et al., 2014)	X									
(WB et al., 1985)										
(Weber et al., 2016)	X									
(Weber et al., 2019)				X						
(Whitworth et al., 2020)			X							
(Wilson et al., 2022)		X								
(Wilson et al., 2023a)				X						
(Wilson et al., 2023b)			X							
(Wiszniewska & Walusiak-Skorupa, 2014)										X
(WJ et al., 1990)					X					
(Wright et al., 2023)	X									
(Xie et al., 2021)	X									
(Yang et al., 2017)	X									
(Yanke et al., 2021)		X								
(Ye et al., 2020)			X							
(Youssef et al., 2023)			X							
(Yüksel, Nørreslet, et al., 2022)		X								

(Yüksel, Sonne, et al., 2022)			X							
(Zahrallayali et al., 2021)			X							
(Zhang et al., 2023)		X								

Table S7. Risk of bias assessment for included studies

	Major domains						Minor domains			
Study	Recruitment procedure and follow-up	Exposure definition and measurement	Outcome source and validation	Confounding	Analysis method	Chronology	Assessor blinding	Funding	Conflict of interest	OVERALL
(Blackley et al., 2023)	✓	✓	✓	✓	✓	✓	✓	✓	✓	✓
(Caridi et al., 2019)	✓	✓	✗	✓	✓	✓	⚠	✓	✓	✗
(Casey et al., 2017)	✗	✗	✗	✗	✓	✓	⚠	✓	✓	✗
(Chang et al., 2018)	⚠	✓	✓	✗	⚠	✓	✗	✓	✓	✗
(Dumas et al., 2012)	✓	✓	✗	✓	✓	✓	✓	✓	✓	✗
(Dumas et al., 2019)	✓	✗	✗	✗	✓	✓	⚠	✓	✓	✗
(Dumas et al., 2020)	✓	✗	✗	✗	✓	✓	⚠	✓	✓	✗
(Dumas, et al., 2021)	✓	✓	✗	✗	✓	✓	⚠	✓	✓	✗
(Dumas, et al., 2017)	✓	✓	✗	✓	✓	✓	⚠	✓	✓	✗
(Estrin et al., 1987)	⚠	✗	✓	✓	✓	✓	⚠	✓	✓	✗
(Gannon et al., 1995)	✗	✗	✗	✗	✗	✗	⚠	⚠	⚠	✗
(Garrido et al., 2022)	✓	✗	✗	✗	✓	✓	⚠	✓	✓	✗
(Gaskins et al., 2017)	✓	✗	✗	✓	✓	✓	⚠	✓	✓	✗
(Gonzalez et al., 2014)	✓	✗	✗	✗	✗	✓	⚠	✓	✓	✗
(Hawley et al., 2018)	✓	✓	✓	✓	✓	✓	⚠	✓	✓	✓
(Kobos et al., 2022)	✓	✗	✗	✓	✓	✓	⚠	✓	✓	✗
(Kurth et al., 2017)	✓	✗	✗	✓	✓	✓	⚠	✓	✓	✗

(Laborde-Castérot et al., 2012)	✗	✗	✓	✗	✗	✗	⚠	✓	✓	✗
(Lee et al., 2014)	✓	✗	✗	✓	✓	✓	⚠	✓	✓	✗
(Mac Hovcová et al., 2013)	✓	✓	✗	✓	✓	✗	✗	✓	✓	✗
(Mehtar et al., 2016)	✓	✗	✗	✓	✓	✓	⚠	✓	✓	✗
(Mwanga et al., 2023)	✓	✓	✓	✓	✓	✓	⚠	✓	✓	✓
(Nayebzadeh, 2007)	✗	✓	✗	✓	✓	✓	✓	⚠	⚠	✗
(Ndlela & Naidoo, 2023)	✓	✓	✓	✓	✓	✓	✓	✓	✓	✓
(Nettis et al., 2002)	✓	✗	✓	✓	✓	✓	⚠	✓	✓	✗
(Norbäck, 1988)	✓	✓	✓	✓	✓	✓	✓	✓	✓	✓
(Otterspoor & Farrell, 2019)	✗	✗	⚠	✗	✗	✗	⚠	⚠	✓	✗
(Patel et al., 2023)	✓	✓	✓	✓	✓	✓	✓	✓	✓	✓
(F. C. Su et al., 2019)	✓	✗	✓	✓	✓	✓	⚠	✓	✓	✗

✓ Low risk; ⚠ Unclear; ✗ High risk;

Table S8. Combined Odds Ratios (ORs) and Confidence Intervals (CIs) for respiratory conditions across studies

Study	Exposure	Outcomes	OR (95%CI), p value	Combined OR (95%CI), p value
Ndlela & Naidoo, 2023	Chlorine-based products	Wheeze without cold	1.48 (0.59-3.68), p>0.05	1.19 (0.73-1.96), p>0.05
Ndlela & Naidoo, 2023		Chronic Cough	1.13 (0.45-2.08), p>0.05	
Ndlela & Naidoo, 2023		Breathlessness	1.04 (0.41-2.64), p>0.05	
Su et al., 2019		Undiagnosed/untreated asthma	3.11 (1.46-6.63), p= 0.003	2.90 (1.69-4.99), p=0.003
Su et al., 2019		Asthma attacks/exacerbations	2.71 (1.25-5.86), p=0.01	
Mwanga et al., 2023	Glutaraldehyde (exposed vs not exposed)	Work related asthma	0.88 (0.29-2.66), p=0.05	1.54 (1.15-2.06), p=0.05
Mwanga et al., 2023	Glutaraldehyde (exposed 1–99 min/week vs not exposed)		0.79 (0.18-3.57), p=0.05	
Mwanga et al., 2023	Glutaraldehyde (exposed ≥100 min/week vs not exposed)		1.45 (0.30-6.95), p=0.05	
Ndlela & Naidoo, 2023	QACs	Wheeze without cold	1.28 (0.49-3.33), p=0.05	1.16 (0.66-2.02), p=0.05
Ndlela & Naidoo, 2023		Chronic Cough	0.69 (0.26-1.82), p=0.05	
Ndlela & Naidoo, 2023		Breathlessness	1.72 (0.66-4.47), p=0.05	
Su et al., 2019	QACs	Mild asthma	0.64 (0.35-1.19) NA	0.87 (0.54-1.38) NA
Su et al., 2019		U-Asthma	1.29 (0.47-3.49) NA	
Su et al., 2019		Asthma exacerbations	1.37 (0.48-3.9) NA	
Lee et al., 2014	Use of spray (medium exposure)	Respiratory symptoms	3.16 (1.24-8.04), p=0.05	2.43 (1.31-4.50), p>0.05
Lee et al., 2014	Use of spray (high exposure)	Respiratory symptoms	1.98 (0.87-4.51), p>0.05	
Gonzalez et al., 2014	GDTs	New-onset asthma	4.68 (1.08-20.22), p=0.03	4.62 (1.62-13.2), p-0.03

<table>
<tr><td>Gonzalez et al., 2014</td><td>GDTs (dilution of disinfectant)</td><td>New-onset asthma</td><td>4.56 (1.0-20.29), p=0.03</td><td></td></tr>
<tr><td>Caridi et al., 2019</td><td>GDTs</td><td>Current asthma</td><td>1.84 (1.26-2.68), p=0.05</td><td rowspan="3">1.95 (1.39-2.74), p=0.05</td></tr>
<tr><td>Caridi et al., 2019</td><td>GDTs</td><td>exacerbation</td><td>3.1 (1.25-7.67), p=0.05</td></tr>
<tr><td>Caridi et al., 2019</td><td>GDTs</td><td>BHR symptom</td><td>1.38 (1.08-1.77), p=0.05</td></tr>
<tr><td>Lee et al., 2014</td><td>GDTs (medium exposure)</td><td>Respiratory symptoms</td><td>2.3 (0.74-7.17), NA</td><td rowspan="2">2.65 (1.16-6.05), NA</td></tr>
<tr><td>Lee et al., 2014</td><td>GDTs (high exposure)</td><td>Respiratory symptoms</td><td>3.11 (0.94-10.3) NA</td></tr>
</table>

Table S9. Studies excluded from the meta-analysis and reasons for exclusion

Intervention/Exposure assessed	Study excluded from meta-analysis	Reason for exclusion
Chlorine-based products	(Mehtar et al., 2016)	Not comparable exposure. It assessed direct spraying of chlorine on healthcare workers rather than environmental exposure alone.
	(Dumas et al., 2019)<br>(Dumas et al., 2020)	Not comparable outcome (the study estimated adjusted hazard ratio instead of odds ratio)
	(Kobos et al., 2022)	Not comparable outcome (skin disorder was quantified instead of respiratory conditions)
Glutaraldehyde	(Dumas et al., 2019)<br>(Dumas et al., 2020)	Not comparable outcome (the study estimated adjusted hazard ratio instead of odds ratio)
	(Dumas, Gaskins, et al., 2021)	Not comparable outcome (the study estimated adjusted hazard ratio instead of odds ratio)
	(Gannon et al., 1995)	Not comparable outcome (the study estimated prevalence instead of odds ratio)
	(Nayebzadeh, 2007)	Not comparable exposure. It assessed the effect of indoor ventilation on glutaraldehyde concentration in air
	(Norbäck, 1988)	Not comparable outcome (the study estimated prevalence instead of odds ratio)
QACs	(Dumas et al., 2019)<br>(Dumas et al., 2020)	Not comparable outcome (the study estimated adjusted hazard ratio instead of odds ratio)
	(Kobos et al., 2022)	Not comparable outcome (FeNO levels were quantified as proxy for respiratory conditions)

	(Mwanga et al., 2023)	Not comparable outcome (skin disorder was quantified instead of respiratory conditions)
Use of spray	(Mehtar et al., 2016)	Not comparable exposure. It assessed direct spraying of chlorine on healthcare workers rather than environmental exposure alone.
General disinfection tasks	(Kurth et al., 2017)	Not comparable outcome (the study estimated prevalence ratio instead of odds ratio)

Table S10. Funnel plots

Funnel plots depicting the relationship between the odds ratios and their standard errors for studies examining the effect of different disinfectant application methods on respiratory conditions. The vertical line represents the overall effect estimate, while the diagonal lines indicate the expected 95% confidence intervals.

Chlorine-based product

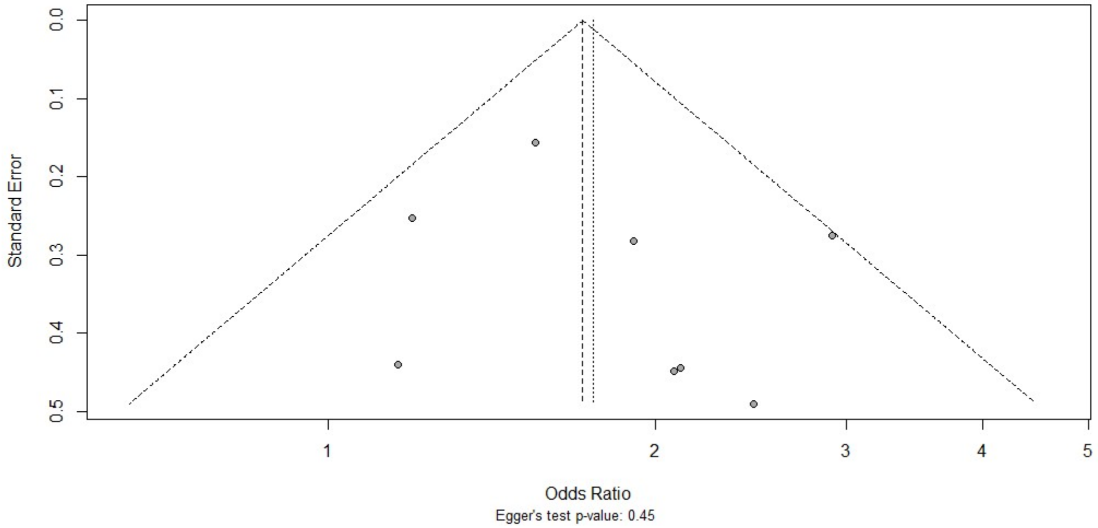

Glutaraldehyde

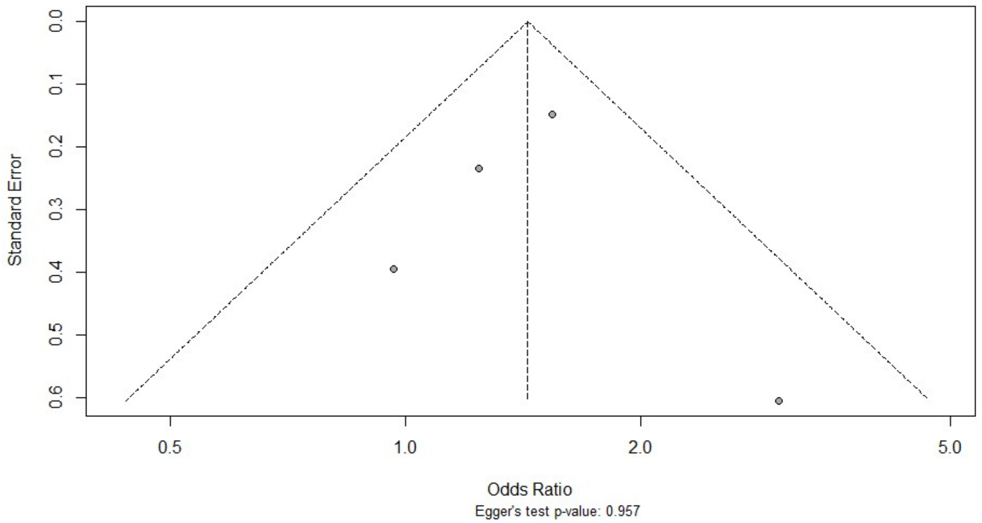

Quaternary Ammonium Compounds

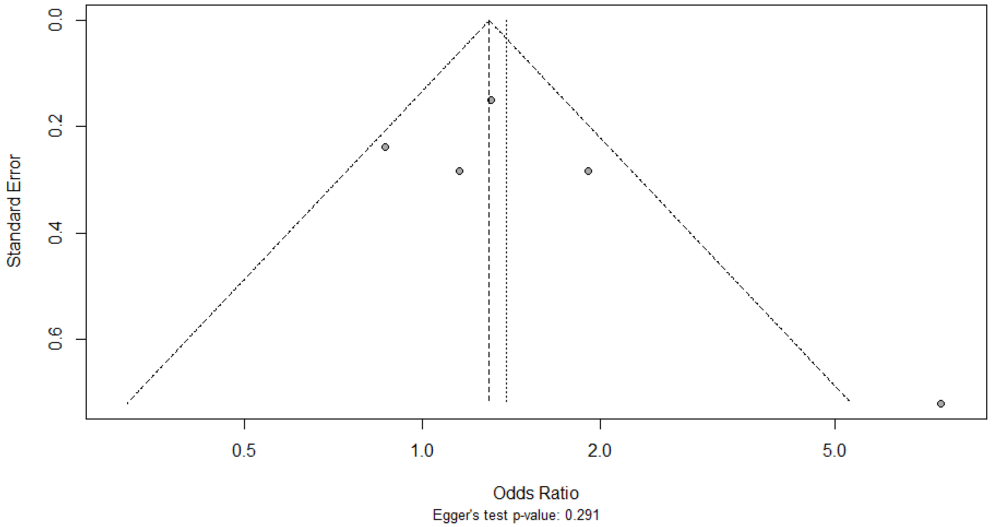

Use of spray

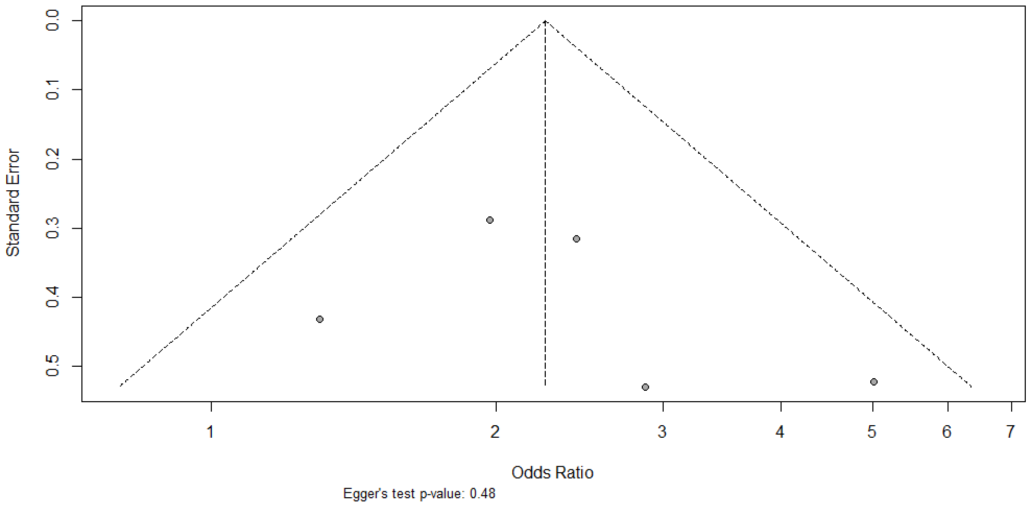

General disinfection tasks

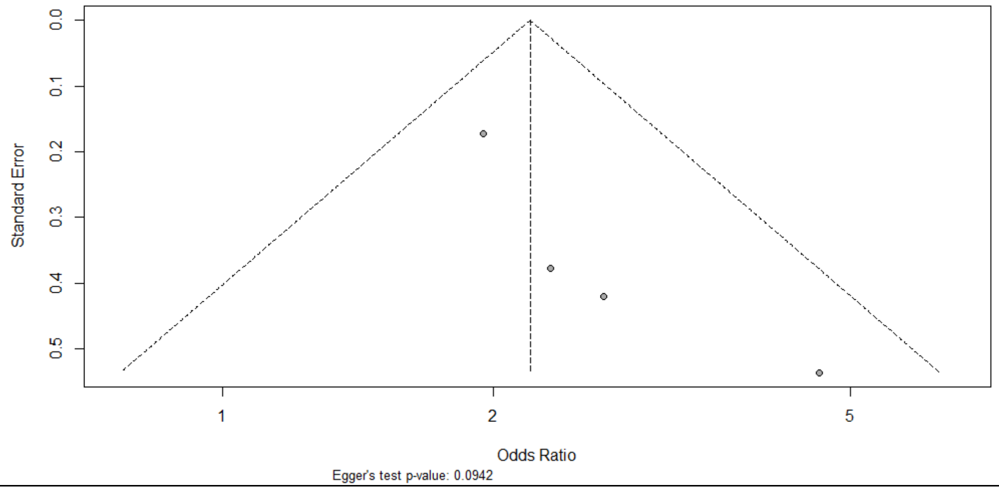

Table S11. Meta-regression results

**Chlorine-Based Product**						
Term	Estimate	Standard error	Z-value	P-value	Cl-low	Cl-high
Interceptor	0.900639566	0.569570809	1.58126005	0.11381859	-0.215698706	2.016977838
Cohort study	-0.207845075	0.663747774	-0.313138639	0.754175334	-1.508766806	1.093076657
Cross-sectional	-0.435980599	0.61387138	-0.710214897	0.477570884	-1.639146396	0.767185198
Sample size	-1.55771E-06	0.000203365	-0.007659639	0.993888552	-0.000400147	0.000397031
**Glutaraldehyde**						
Interceptor	0.837914077	1.076665414	0.778249274	0.436422074	-1.272311358	2.948139512
Cross-sectional	-0.253837148	0.786363031	-0.322798934	0.74684752	-1.795080369	1.287406072
Sample size	-0.000185195	0.000414202	-0.447111889	0.654794275	-0.000997015	0.000626626
**QACs**						
Cross-sectional	NA	NA	NA	NA	NA	NA
Interceptor*	0.615326785	0.530150554	1.16066423	0.245778474	-0.423749208	1.654402777
Sample size	-9.68933E-05	0.000210533	-0.4602292	0.645351714	-0.00050953	0.000315743
**Use of spray**						
Interceptor	1.107540023	0.716192161	1.546428574	0.122001098	-0.296170819	2.511250865
Cross-sectional	-0.21613373	0.745346564	-0.289977495	0.771833455	-1.67698615	1.244718691
Sample size	-7.35193E-05	0.000306358	-0.23997877	0.810346715	-0.000673969	0.000526931
**General Disinfection Tasks (GDT)**						
Interceptor	1.044237568	0.408096839	2.558798469	0.010503461	0.244382461	1.844092675
Cross-sectional	0.20012901	0.454762932	0.440073269	0.659884042	-0.691189958	1.091447978

Sample size	-0.000279931	0.0002168	-1.291198843	0.196634736	-0.000704851	0.000144988
*Only sample size was included as moderator as all included studies were cross-sectional studies						

Table S12. Leave-one-out analysis results

Forest plots showing the odds ratios (OR) with 95% confidence intervals (CI) for respiratory conditions associated with different disinfectants and application methods exposure after omitting individual studies. The square shapes represent the individual results. The diamond shape represents the overall effect estimate from the common effect model.

Leave-one-out chlorine-based products

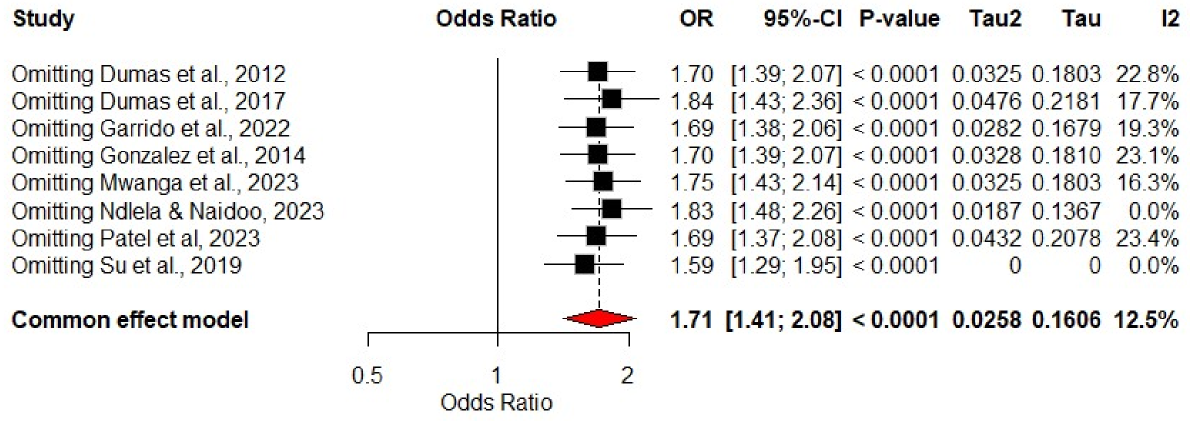

Leave-one-out glutaraldehyde

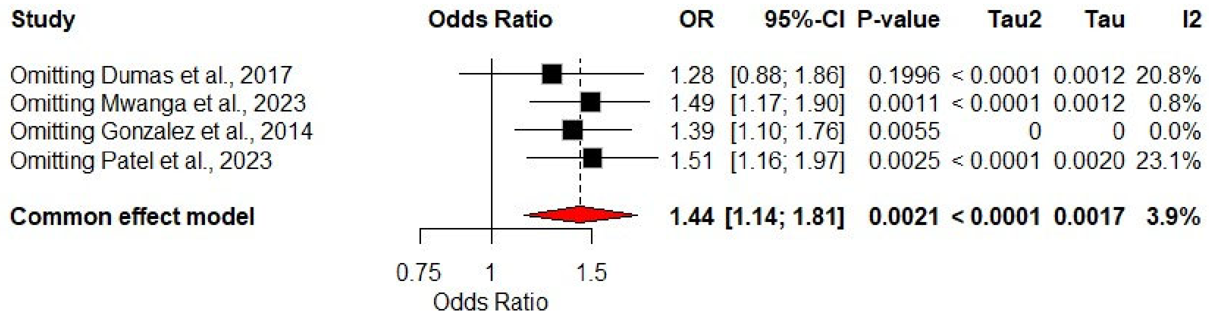

Leave-one-out QACs

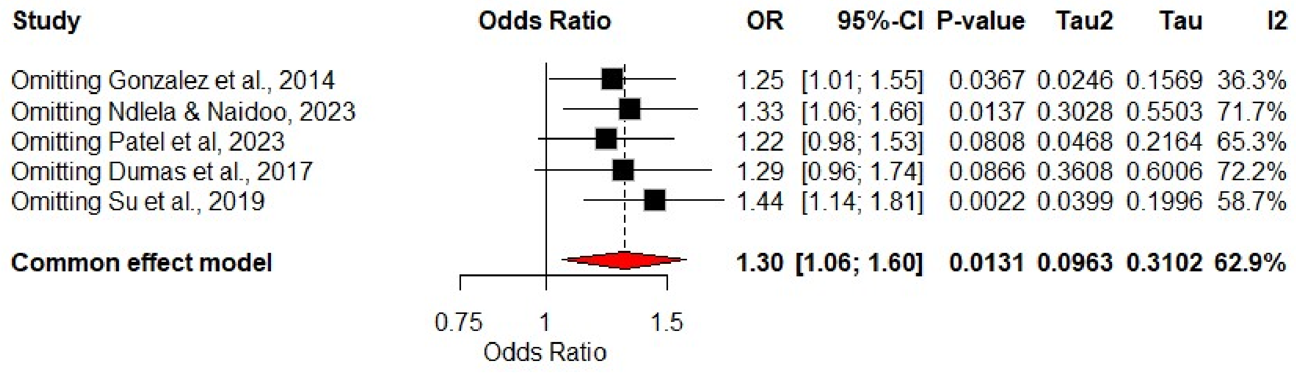

Leave-one-out use of spray

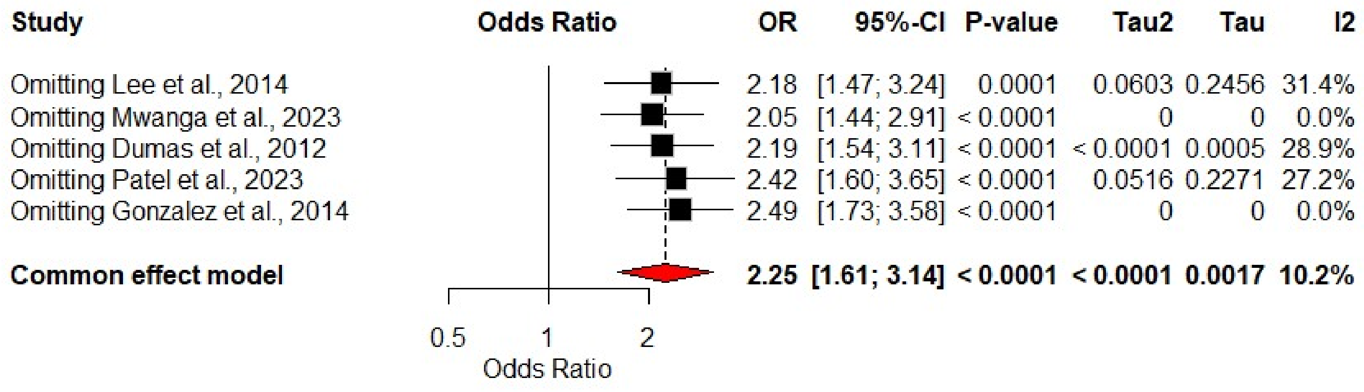

Leave-one-out general disinfection tasks

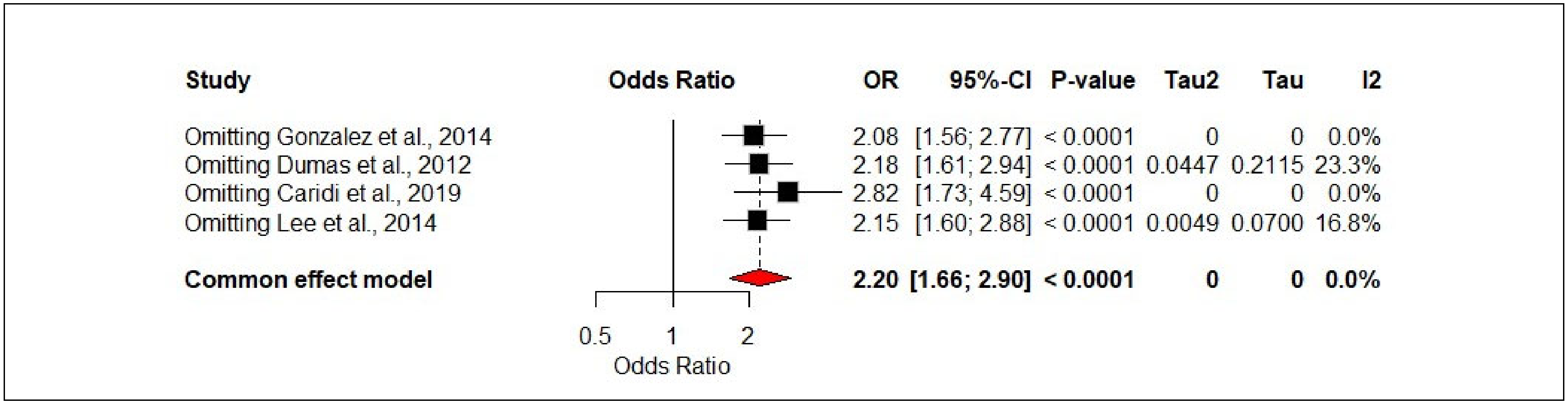
Study
Odds Ratio
OR
95%-CI
P-value
Tau2
Tau
I2
Omitting Gonzalez et al., 2014 2.08 [1.56; 2.77] < 0.0001 0 0 0.0%
Omitting Dumas et al., 2012 2.18 [1.61; 2.94] < 0.0001 0.0447 0.2115 23.3%
Omitting Caridi et al., 2019 2.82 [1.73; 4.59] < 0.0001 0 0 0.0%
Omitting Lee et al., 2014 2.15 [1.60; 2.88] < 0.0001 0.0049 0.0700 16.8%
Common effect model 2.20 [1.66; 2.90] < 0.0001 0 0 0.0%
0.5
1
2
Odds Ratio

References

AD, L., DC, C., & KT, K. (1993). Utility of the complete blood count in routine medical surveillance for ethylene oxide exposure. *American Journal of Industrial Medicine*, *24*(2), 191–206. https://doi.org/10.1002/ajim.4700240206

AD, T., Grummt, T., Törnqvist, M., PB, F., FJ, van D., H, van M., HM, S., Osterman-Golkar, S., Uebel, C., YS, T., & al., et. (1991). Biological and chemical monitoring of occupational exposure to ethylene oxide. *Mutation Research*, *250*(1), 483–497. https://doi.org/10.1016/0027-5107(91)90205-3

Afework, A., Tamene, A., Tafa, A., Tesfaye, A., & Gemede, S. (2023). The Prevalence of Occupational Accidents and the Associated Factors Among Janitorial Staff at a University Teaching Hospital in South Ethiopia. *Risk Management and Healthcare Policy*, *16*, 1499–1507. https://doi.org/10.2147/RMHP.S425313

Agathos, M., & HA, B. (1982). [Hand dermatitis in medical personnel]. In *Dermatosen in Beruf und Umwelt. Occupation and environment* (Vol. 30, Issue 2, pp. 43–47). https://pubmed.ncbi.nlm.nih.gov/6211342/

Agthe, N., Terho, K., Kurvinen, T., Routamaa, M., Peltonen, R., Laitinen, K., & Kanerva, M. (2009). Microbiological efficacy and tolerability of a new, non-alcohol-based hand disinfectant. *Infection Control and Hospital Epidemiology*, *30*(7), 685–690. https://doi.org/10.1086/598239

Ahmed-Lecheheb, D., Cunat, L., Hartemann, P., & Hautemanière, A. (2012). Dermal and pulmonary absorption of ethanol from alcohol-based hand rub. *The Journal of Hospital Infection*, *81*(1), 31–35. https://doi.org/10.1016/j.jhin.2012.02.006

Alexandre, A. C. S., Neto, N. M. G., Silva, M. A. S., Santos, D. C. S., Alcoforado, J. M. S. G., & de Melo, D. B. (2021). Construction and validation of checklist for disinfecting ambulances to transport Covid-19 patients. *Revista Gaucha de Enfermagem*, *42*. https://doi.org/10.1590/1983-1447.2021.20200312

Alexis DESCATHA Jean-Baptiste BARBE-RICHAUD, Jeremy BLOTTIAUX, G. C. (2022). Human chlorine gas exposition and its management- an umbrella review on human data. *Critical Reviews in Toxicology*, *52*, 1–19. https://doi.org/10.1080/10408444.2022.2035317

Alimohammadi, M., & Naderi, M. (2021). Effectiveness of Ozone Gas on Airborne Virus Inactivation in Enclosed Spaces: A Review Study.

Ozone: Science and Engineering, *43*(1), 21–31. https://doi.org/10.1080/01919512.2020.1822149

Alnimr, A., Alamri, A., Salama, K. F., Radi, M., Bukharie, H., Alshehri, B., Rabaan, A. A., & Alshahrani, M. (2021). The environmental deposition of severe acute respiratory syndrome coronavirus 2 in nosocomial settings: Role of the aerosolized hydrogen peroxide. *Risk Management and Healthcare Policy*, *14*, 4469–4475. https://doi.org/10.2147/RMHP.S336085

Ambrosino, A., Pironti, C., Dell'Annunziata, F., Giugliano, R., Chianese, A., Moccia, G., DeCaro, F., Galdiero, M., Franci, G., & Motta, O. (2022). Investigation of biocidal efficacy of commercial disinfectants used in public, private and workplaces during the pandemic event of SARS-CoV-2. *Scientific Reports*, *12*(1). https://doi.org/10.1038/s41598-022-09575-1

Amini Tapouk, F., Nabizadeh, R., Mirzaei, N., Hosseini Jazani, N., Yousefi, M., & Valizade Hasanloei, M. A. (2020). Comparative efficacy of hospital disinfectants against nosocomial infection pathogens. *Antimicrobial Resistance and Infection Control*, *9*(1). https://doi.org/10.1186/s13756-020-00781-y

Amlôt, R., Carter, H., Riddle, L., Larner, J., & RP, C. (2017). Volunteer trials of a novel improvised dry decontamination protocol for use during mass casualty incidents as part of the UK'S Initial Operational Response (IOR). *PloS One*, *12*(6), e0179309. https://doi.org/10.1371/journal.pone.0179309

Amr, S., & Bollinger, M. E. (2004). Latex allergy and occupational asthma in healh care workers: Adverse outcomes. *Environmental Health Perspectives*, *112*(3), 378–381. https://doi.org/10.1289/ehp.6612

Angelova-Fischer, I., Stilla, T., Kezic, S., Fischer, T. W., & Zillikens, D. (2016). Barrier function and natural moisturizing factor levels after cumulative exposure to short-chain aliphatic alcohols and detergents: Results of occlusion-modified tandem repeated irritation test. *Acta Dermato-Venereologica*, *96*(7), 880–884. https://doi.org/10.2340/00015555-2363

Anggraini, D., & Lestari, K. S. (2022). IMPLEMENTATION OF ENVIRONMENTAL SANITATION AND DISINFECTION IN HOSPITALS TO REDUCE THE SPREAD OF COVID19: A LITERATURE REVIEW. *Jurnal Kesehatan Lingkungan*, *14*(1), 63–70. https://doi.org/10.20473/jkl.v14i1.2022.63-70

Antolín, E., Herrero, B., Rodríguez, R., Illescas, T., Duyos, I., Gimeno, A., Sotillo, L., Abascal, A., Orensanz, I., Hernández, A., & Bartha, J. L. (2021). How to organize a Fetal Medicine Unit in the context of COVID-19 pandemic. Safe measures for obstetric scans and equipment cleaning. *Clinica e Investigacion En Ginecologia y Obstetricia*, *48*(1), 3–13. https://doi.org/10.1016/j.gine.2020.06.013

Archangelidi, O., Sathiyajit, S., Consonni, D., Jarvis, D., & De Matteis, S. (2021). Cleaning products and respiratory health outcomes in occupational cleaners: A systematic review and meta-analysis. *Occupational and Environmental Medicine*, *78*(8), 541–547. https://doi.org/10.1136/OEMED-2020-106776

Assadian, O., Harbarth, S., Vos, M., JK, K., Asensio, A., & AF, W. (2021). Practical recommendations for routine cleaning and disinfection procedures in healthcare institutions: a narrative review. *The Journal of Hospital Infection*, *113*, 104–114. https://doi.org/10.1016/j.jhin.2021.03.010

Astrid, F., Beata, Z., Julia, E., Elisabeth, P., & Magda, D.-E. (2021). The use of a UV-C disinfection robot in the routine cleaning process: a field study in an Academic hospital. *Antimicrobial Resistance and Infection Control*, *10*(1). https://doi.org/10.1186/s13756-021-00945-4

Azmi, N. L., Bakar, N. M. A., Rashid, N. I. A., Mafauzy, N. M., Mohamed, A. S. M. H., Mohamad, N. A., Said, S. H., Zawawi, N. I. A., & Mohamed, N. S. F. (2021). Development and validation of a questionnaire on knowledge and practices of disinfection and sterilization among healthcare workers. *Gulhane Medical Journal*, *63*(4), 280–286. https://doi.org/10.4274/GULHANE.GALENOS.2021.1564

Babić, A., Turk, R., & MacAn, J. (2020). Toxicological aspects of increased use of surface and hand disinfectants in Croatia during the COVID-19 pandemic: A preliminary report. *Arhiv Za Higijenu Rada i Toksikologiju*, *71*(3), 261–264. https://doi.org/10.2478/aiht-2020-71-3470

Baccolini, V., D'Egidio, V., P, de S., Migliara, G., Massimi, A., Alessandri, F., Tellan, G., Marzuillo, C., C, D. V., MV, R., & Villari, P. (2019). Effectiveness over time of a multimodal intervention to improve compliance with standard hygiene precautions in an intensive care unit of a large teaching hospital. In *Antimicrobial resistance and infection control* (Vol. 8, p. 92). https://doi.org/10.1186/s13756-019-0544-0

Barbaud, A. (2002). [Occupational dermatitis in health care personnel]. In *La Revue du praticien* (Vol. 52, Issue 13, pp. 1425–1432). https://pubmed.ncbi.nlm.nih.gov/12385152/

Bardaweel, S. K., Almuhaissen, S. A., Abdul-Hadi, A. A., Al-Masri, Q. S., & Musleh, H. R. (2023). Knowledge and Practices of Disinfectants and Sanitizers Use during COVID-19 Pandemic in Jordan. *Jordan Journal of Pharmaceutical Sciences*, *16*(1), 82–95. https://doi.org/10.35516/jjps.v16i1.1065

Batalla, A., García-Doval, I., & de la Torre, C. (2012). Products for Hand Hygiene and Antisepsis: Use by Health Professionals and Relationship With Hand Eczema. *Actas Dermo-Sifiliográficas (English Edition)*, *103*(3), 192–197. https://doi.org/https://doi.org/10.1016/j.adengl.2012.04.004

Bauer, A., Pesonen, M., Brans, R., Caroppo, F., Dickel, H., Dugonik, A., Larese Filon, F., Geier, J., Gimenez-Arnau, A. M., Napolitano, M., Patruno, C., Rustemeyer, T., Simon, D., Schuttelaar, M. L. A., Spiewak, R., Stingeni, L., Vok, M., Weisshaar, E., Wilkinson, M., … Uter, W. (2023). Occupational contact allergy: The European perspective–Analysis of patch test data from ESSCA between 2011 and 2020. *Contact Dermatitis*, *88*(4), 263–274. https://doi.org/10.1111/cod.14280

Bédard, A., Varraso, R., Sanchez, M., Clavel-Chapelon, F., Zock, J.-P., Kauffmann, F., & Le Moual, N. (2014). Cleaning sprays, household help and asthma among elderly women. *Respiratory Medicine*, *108*(1), 171–180. https://doi.org/10.1016/j.rmed.2013.10.018

Berger, D., Gundermann, G., Sinha, A., Moroi, M., Goyal, N., & Tsai, A. (2022). Review of aerosolized hydrogen peroxide, vaporized hydrogen peroxide, and hydrogen peroxide gas plasma in the decontamination of filtering facepiece respirators. *American Journal of Infection Control*, *50*(2), 203–213. https://doi.org/10.1016/j.ajic.2021.06.012

Bernardini, P., Carelli, G., Rimatori, V., & Contegiacomo, P. (1983). Health hazard for hospital workers from exposure to formaldehyde. *La Medicina Del Lavoro*, *74*(2), 106–110. https://pubmed.ncbi.nlm.nih.gov/6865837/

Bessonneau, V., Mosqueron, L., Berrubé, A., Mukensturm, G., Buffet-Bataillon, S., Gangneux, J.-P., & Thomas, O. (2013). VOC Contamination in Hospital, from Stationary Sampling of a Large Panel of Compounds, in View of Healthcare Workers and Patients Exposure Assessment. *PLoS ONE*, *8*(2). https://doi.org/10.1371/journal.pone.0055535

Bhat, S. A., Sher, F., Kumar, R., Karahmet, E., Haq, S. A. U., Zafar, A., & Lima, E. C. (2022). Environmental and health impacts of spraying COVID-19 disinfectants with associated challenges. *Environmental Science and Pollution Research*, *29*(57), 85648–85657.

https://doi.org/10.1007/s11356-021-16575-7

Bijlsma, N., & Cohen, M. M. (2016). Environmental chemical assessment in clinical practice: Unveiling the elephant in the room. *International Journal of Environmental Research and Public Health*, *13*(2). https://doi.org/10.3390/ijerph13020181

Blackley, B. H., Nett, R. J., Cox-Ganser, J. M., Harvey, R. R., & Virji, M. A. (2023). Eye and airway symptoms in hospital staff exposed to a product containing hydrogen peroxide, peracetic acid, and acetic acid. *American Journal of Industrial Medicine*, *66*(8), 655–669. https://doi.org/10.1002/AJIM.23488

Brune, Z., Kuschner, C. E., Mootz, J., Davidson, K. W., Pena, R. C. F., Ghanem, M. H., Fischer, A., Gitman, M., Teperman, L., Mason, C., & Becker, L. B. (2021). Effectiveness of sars-cov-2 decontamination and containment in a covid-19 icu. *International Journal of Environmental Research and Public Health*, *18*(5), 1–9. https://doi.org/10.3390/ijerph18052479

Burgaz, S., Rezanko, R., Kara, S., & Karakaya, A. E. (1992). Thioethers in urine of sterilization personnel exposed to ethylene oxide. *Journal of Clinical Pharmacy and Therapeutics*, *17*(3), 169–172. https://doi.org/10.1111/j.1365-2710.1992.tb01287.x

Byrns, G., Barham, B., Yang, L., Webster, K., Rutherford, G., Steiner, G., Petras, D., & Scannell, M. (2017). The uses and limitations of a hand-held germicidal ultraviolet wand for surface disinfection. *Journal of Occupational and Environmental Hygiene*, *14*(10), 749–757. https://doi.org/10.1080/15459624.2017.1328106

Caetano, M. H., Zen Siqueira, J. P., De Andrade, D., De Sousa, Á. F. L., Rigotti, M. A., Diniz, M. O., De Almeida, W. A., Ferreira, A. M., & De Almeida, M. T. G. (2021). Antimicrobial action of ozone gas on surfaces and in the air. *ACTA Paulista de Enfermagem*, *34*. https://doi.org/10.37689/ACTA-APE/2021AO02712

Caridi, M. N., Humann, M. J., Liang, X., Su, F. C., Stefaniak, A. B., LeBouf, R. F., Stanton, M. L., Virji, M. A., & Henneberger, P. K. (2019). Occupation and task as risk factors for asthma-related outcomes among healthcare workers in New York City. *International Journal of Hygiene and Environmental Health*, *222*(2), 211–220. https://doi.org/10.1016/J.IJHEH.2018.10.001

Carling, P. C. (2021). Health Care Environmental Hygiene: New Insights and Centers for Disease Control and Prevention Guidance. *Infectious*

Disease Clinics of North America, *35*(3), 609–629. https://doi.org/10.1016/j.idc.2021.04.005

Carling, P. C., Parry, M. F., & Olmstead, R. (2023). Environmental approaches to controlling Clostridioides difficile infection in healthcare settings. *Antimicrobial Resistance and Infection Control*, *12*(1). https://doi.org/10.1186/s13756-023-01295-z

Carol Sharma, B. G. H., Kumar, S. D., & Ghosh, S. (2023). Prospects and retrospects of occupational hazards amongst healthcare workers. *Biomedicine (India)*, *43*(1), 1–7. https://doi.org/10.51248/.v43i1.2562

Carvalho, A. P. A., & Conte-Junior, C. A. (2021). Recent Advances on Nanomaterials to COVID-19 Management: A Systematic Review on Antiviral/Virucidal Agents and Mechanisms of SARS-CoV-2 Inhibition/Inactivation. *Global Challenges*, *5*(5). https://doi.org/10.1002/gch2.202000115

Casey, M. L., Hawley, B., Edwards, N., Cox-Ganser, J. M., & Cummings, K. J. (2017). Health problems and disinfectant product exposure among staff at a large multispecialty hospital. *American Journal of Infection Control*, *45*(10), 1133–1138. https://doi.org/10.1016/J.AJIC.2017.04.003

CC, H., CF, W., WC, S., MF, C., CY, C., YC, C., SH, L., SY, C., & KY, W. (2011). Comparative analysis of urinary N7-(2-hydroxyethyl)guanine for ethylene oxide- and non-exposed workers. In *Toxicology letters* (Vol. 202, Issue 3, pp. 237–243). https://doi.org/10.1016/j.toxlet.2011.02.009

Cebeci, D., Karasel, S., Rıfkı, D., Yesildağlı, H., & Kalfaoglu, M. (2021). The Effect of Personal Protective Equipment (PPE) and Disinfectants on Skin Health During Covid 19 Pandemia. *Medical Archives*, *75*(5), 361–365. https://doi.org/10.5455/medarh.2021.75.361-365

Centers for Diseases Prevention and Control. (1984). Dermatitis among hospital workers--Oregon. *MMWR. Morbidity and Mortality Weekly Report*, *33*(48), 681–682. https://pubmed.ncbi.nlm.nih.gov/6239090/

Chaari, N., Sakly, A., Amri, C., Mahfoudh, A., MA, H., Khalfallh, T., Bchir, N., & Akrout, M. (2010). Occupational allergy in healthcare workers. *Recent Patents on Inflammation & Allergy Drug Discovery*, *4*(1), 65–74. https://doi.org/10.2174/187221310789895630

Chang, Y. B., Lee, F. Y., Goh, M. M., Lam, D. K. H., & Tan, A. B. H. (2018). Assessment of occupational exposure to airborne chlorine dioxide

of healthcare workers using impregnated wipes during high-level disinfection of non-lumened flexible nasoendoscopes. *Journal of Occupational and Environmental Hygiene*, *15*(12), 818–823. https://doi.org/10.1080/15459624.2018.1523617

Charlier, B., Coglianese, A., De Rosa, F., De Caro, F., Piazza, O., Motta, O., Borrelli, A., Capunzo, M., Filippelli, A., & Izzo, V. (2021). Chemical risk in hospital settings: Overview on monitoring strategies and international regulatory aspects. *Journal of Public Health Research*, *10*(1). https://doi.org/10.4081/JPHR.2021.1993

Choi, H., Chatterjee, P., Lichtfouse, E., Martel, J. A., Hwang, M., Jinadatha, C., & Sharma, V. K. (2021). Classical and alternative disinfection strategies to control the COVID-19 virus in healthcare facilities: a review. *Environmental Chemistry Letters*, *19*(3), 1945–1951. https://doi.org/10.1007/s10311-021-01180-4

CL, H., AM, P., JL, C., & RL, N. (2016). Occupational skin disease among Australian healthcare workers: a retrospective analysis from an occupational dermatology clinic, 1993-2014. *Contact Dermatitis*, *75*(4), 213–222. https://doi.org/10.1111/cod.12616

Clausen, P. A., Frederiksen, M., Sejbæk, C. S., Sørli, J. B., Hougaard, K. S., Frydendall, K. B., Carøe, T. K., Flachs, E. M., Meyer, H. W., Schlünssen, V., & Wolkoff, P. (2020). Chemicals inhaled from spray cleaning and disinfection products and their respiratory effects. A comprehensive review. *International Journal of Hygiene and Environmental Health*, *229*. https://doi.org/10.1016/j.ijheh.2020.113592

Cochrane, S. A., Arts, J. H. E., Ehnes, C., Hindle, S., Hollnagel, H. M., Poole, A., Suto, H., & Kimber, I. (2015). Thresholds in chemical respiratory sensitisation. *Toxicology*, *333*, 179–194. https://doi.org/10.1016/j.tox.2015.04.010

Coggon, D., Harris, E. C., Poole, J., & Palmer, K. T. (2004). Mortality of workers exposed to ethylene oxide: Extended follow up of a British cohort. *Occupational and Environmental Medicine*, *61*(4), 358–362. https://doi.org/10.1136/oem.2003.008268

CP, H., LG, D., & PA, R. (2005). Occupation-related allergies in dentistry. *Journal of the American Dental Association (1939)*, *136*(4), 500–510. https://doi.org/10.14219/jada.archive.2005.0207

Cucurachi, G., & MG, T. (2010). [Disinfectants for the skin of premature]. *Minerva pediatrica*, *62*(3), 157–159. https://pubmed.ncbi.nlm.nih.gov/21090087/

Curran, E. T., Wilkinson, M., & Bradley, T. (2019). Chemical disinfectants: Controversies regarding their use in low risk healthcare environments (part 1). *Journal of Infection Prevention*, *20*(2), 76–82. https://doi.org/10.1177/1757177419828139

Cutts, T., Kasloff, S., Safronetz, D., & Krishnan, J. (2021). Decontamination of common healthcare facility surfaces contaminated with SARS-CoV-2 using peracetic acid dry fogging. *Journal of Hospital Infection*, *109*, 82–87. https://doi.org/10.1016/j.jhin.2020.12.016

Cutuli, M. A., Guarnieri, A., Pietrangelo, L., Magnifico, I., Venditti, N., Recchia, L., Mangano, K., Nicoletti, F., Marco, R. D., & Petronio, G. P. (2021). Potential mucosal irritation discrimination of surface disinfectants employed against sars-cov-2 by limacus flavus slug mucosal irritation assay. *Biomedicines*, *9*(4). https://doi.org/10.3390/biomedicines9040424

CY, L., HX, G., Li, J., & WP, T. (2003). Effects of iodophor on the thyroid glands of female medical workers. In *Zhonghua yi xue za zhi* (Vol. 83, Issue 8, pp. 647–649). https://pubmed.ncbi.nlm.nih.gov/12887820/

Daba, C., Gebrehiwot, M., Asefa, L., Lemma, H., Atamo, A., Kebede, E., Embrandiri, A., & Debela, S. A. (2022). Occupational safety of janitors in Ethiopian University during COVID-19 pandemic: Results from observational study. *Frontiers in Public Health*, *10*. https://doi.org/10.3389/fpubh.2022.895977

Dalton, P. H., Maute, C., Hicks, J. B., Watson, H. N., Loccisano, A. E., & Kerger, B. D. (2023). Environmental chamber studies of eye and respiratory irritation from use of a peracetic acid-based hospital surface disinfectant. *Antimicrobial Stewardship and Healthcare Epidemiology*, *3*(1). https://doi.org/10.1017/ASH.2023.138

Davidovits, M., Barak, A., Cleper, R., Krause, I., Gamzo, Z., & Eisenstein, B. (2003). Methaemoglobinaemia and haemolysis associated with hydrogen peroxide in a paediatric haemodialysis centre: a warning note. *Nephrology, Dialysis, Transplantation : Official Publication of the European Dialysis and Transplant Association - European Renal Association*, *18*(11), 2354–2358. https://doi.org/10.1093/ndt/gfg395

De Groot, R., Van Zoelen, G. A., Leenders, M. E. C., Van Riel, A. J. H. P., De Vries, I., & De Lange, D. W. (2021). Is secondary chemical exposure of hospital personnel of clinical importance? *Clinical Toxicology*, *59*(4), 269–278. https://doi.org/10.1080/15563650.2020.1860216

de Oliveira, C. R., de Oliveira Carvalho, M. C., Schmitz, G. V, de Souza Botelho Almeida, T., Carvalho, H. C., Fernandes, A. B., & de Lima, C. J.

(2023). Ozonated water in disinfection of hospital instrument table. *Research on Biomedical Engineering*, *39*(2), 329–334. https://doi.org/10.1007/s42600-023-00272-0

De Troeyer, K., De Man, J., Vandebroek, E., Vanoirbeek, J. A., Hoet, P. H. M., Nemery, B., Vanroelen, C., Casas, L., & Ronsmans, S. (2022). Identifying cleaning products associated with short-term work-related respiratory symptoms: A workforce-based study in domestic cleaners. *Environment International*, *162*, 107170. https://doi.org/https://doi.org/10.1016/j.envint.2022.107170

Delclos, G. L., Gimeno, D., Arif, A. A., Burau, K. D., Carson, A., Lusk, C., Stock, T., Symanski, E., Whitehead, L. W., Zock, J.-P., Benavides, F. G., & Antó, J. M. (2007). Occupational risk factors and asthma among health care professionals. *American Journal of Respiratory and Critical Care Medicine*, *175*(7), 667–675. https://doi.org/10.1164/rccm.200609-1331OC

Descatha, A., Hamzaoui, H., Takala, J., & Oppliger, A. (2023). A systematised overview of published reviews on biological hazards, occupational health and safety. *Safety and Health at Work*. https://doi.org/https://doi.org/10.1016/j.shaw.2023.10.008

Dhama, K., Patel, S. K., Kumar, R., Masand, R., Rana, J., Yatoo, M. I., Tiwari, R., Sharun, K., Mohapatra, R. K., Natesan, S., Dhawan, M., Ahmad, T., Emran, T. B., Malik, Y. S., & Harapan, H. (2021). The role of disinfectants and sanitizers during COVID-19 pandemic: advantages and deleterious effects on humans and the environment. *Environmental Science and Pollution Research*, *28*(26), 34211–34228. https://doi.org/10.1007/s11356-021-14429-w

Ding, M., Lawson, C., Johnson, C., Rich-Edwards, J., Gaskins, A. J., Boiano, J., Henn, S., Rocheleau, C., & Chavarro, J. E. (2021). Occupational exposure to high-level disinfectants and risk of miscarriage among nurses. *Occupational and Environmental Medicine*, *78*(10), 731–737. https://doi.org/10.1136/OEMED-2020-107297

DJ, T., Haines, T., Lawrence, M., & Rosa, N. (1993). A study of sister chromatid exchange and somatic cell mutation in hospital workers exposed to ethylene oxide. *Environmental Health Perspectives*, *101*, 159–164. https://doi.org/10.1289/ehp.93101s3159

Doll, M., Stevens, M., & Bearman, G. (2018). Environmental cleaning and disinfection of patient areas. *International Journal of Infectious Diseases*, *67*, 52–57. https://doi.org/10.1016/j.ijid.2017.10.014

Donnay, C., Denis, M.-A., Magis, R., Fevotte, J., Massin, N., Dumas, O., Pin, I., Choudat, D., Kauffmann, F., & Le Moual, N. (2011). Under-estimation of self-reported occupational exposure by questionnaire in hospital workers. *Occupational and Environmental Medicine*, *68*(8), 611–617. https://doi.org/10.1136/oem.2010.061671

Doolan, B. T., & Crilly, H. M. (2019). Chlorhexidine wipes: Time to stop and think about allergy. *Anaesthesia and Intensive Care*, *47*(1), 90–95. https://doi.org/10.1177/0310057X18811974

Dore, M. A., Torabizadeh, C., & Keshtkaran, Z. (2022). Threats to operating room personnel's occupational safety and health: a qualitative study. *Anaesthesia, Pain and Intensive Care*, *26*(3), 368–381. https://doi.org/10.35975/apic.v26i3.1912

Dorgham, N. A., & Dorgham, D. A. (2021). Disinfectants and skin antiseptics for safe prophylaxis against covid-19, review of literature. *Open Dermatology Journal*, *15*(1), 16–22. https://doi.org/10.2174/1874372202115010016

Dotson, G. S., Lotter, J. T., Zisook, R. E., Gaffney, S. H., Maier, A., & Colvin, J. (2020). Setting occupational exposure limits for antimicrobial agents: A case study based on a quaternary ammonium compound-based disinfectant. *Toxicology and Industrial Health*, *36*(9), 619–633. https://doi.org/10.1177/0748233720970438

Dumas, O. (2021). Cleaners and airway diseases. *Current Opinion in Allergy and Clinical Immunology*, *21*(2), 101–109. https://doi.org/10.1097/ACI.0000000000000710

Dumas, O., Bédard, A., Marbac, M., Sedki, M., Temam, S., Chanoine, S., Severi, G., Boutron-Ruault, M.-C., Garcia-Aymerich, J., Siroux, V., Varraso, R., & Le Moual, N. (2021). Household Cleaning and Poor Asthma Control Among Elderly Women. *Journal of Allergy and Clinical Immunology: In Practice*, *9*(6), 2358-2365.e4. https://doi.org/10.1016/j.jaip.2021.02.022

Dumas, O., Boggs, K. M., Quinot, C., Varraso, R., Zock, J. P., Henneberger, P. K., Speizer, F. E., Le Moual, N., & Camargo, C. A. (2020). Occupational exposure to disinfectants and asthma incidence in U.S. nurses: A prospective cohort study. *American Journal of Industrial Medicine*, *63*(1), 44–50. https://doi.org/10.1002/AJIM.23067

Dumas, O., Donnay, C., Heederik, D. J. J., Héry, M., Choudat, D., Kauffmann, F., & Moual, N. Le. (2012). Occupational exposure to cleaning

products and asthma in hospital workers. *Occupational and Environmental Medicine*, *69*(12), 883–889. https://doi.org/10.1136/OEMED-2012-100826

Dumas, O., Gaskins, A. J., Boggs, K. M., Henn, S. A., Le Moual, N., Varraso, R., Chavarro, J. E., & Camargo, C. A. (2021). Occupational use of high-level disinfectants and asthma incidence in early to mid-career female nurses: a prospective cohort study. *Occupational and Environmental Medicine*, *78*(4), 244. https://doi.org/10.1136/OEMED-2020-106793

Dumas, O., & Le Moual, N. (2020). Damaging effects of household cleaning products on the lungs. *Expert Review of Respiratory Medicine*, *14*(1), 1–4. https://doi.org/10.1080/17476348.2020.1689123

Dumas, O., Siroux, V., Luu, F., Nadif, R., Zock, J.-P., Kauffmann, F., & Le Moual, N. (2014). Cleaning and asthma characteristics in women. *American Journal of Industrial Medicine*, *57*(3), 303–311. https://doi.org/10.1002/ajim.22244

Dumas, O., Varraso, R., Boggs, K. M., Quinot, C., Zock, J. P., Henneberger, P. K., Speizer, F. E., Le Moual, N., & Camargo, C. A. (2019). Association of Occupational Exposure to Disinfectants With Incidence of Chronic Obstructive Pulmonary Disease Among US Female Nurses. *JAMA Network Open*, *2*(10), e1913563–e1913563. https://doi.org/10.1001/JAMANETWORKOPEN.2019.13563

Dumas, O., Varraso, R., Zock, J. P., Henneberger, P. K., Speizer, F. E., Wiley, A. S., Le Moual, N., & Camargo, C. A. (2015). Asthma history, job type and job changes among US nurses. *Occupational and Environmental Medicine*, *72*(7), 482–488. https://doi.org/10.1136/oemed-2014-102547

Dumas, O., Wiley, A. S., Henneberger, P. K., Speizer, F. E., Zock, J.-P., Varraso, R., Le Moual, N., Boggs, K. M., & Camargo, C. A. (2017). Determinants of disinfectant use among nurses in U.S. healthcare facilities. *American Journal of Industrial Medicine*, *60*(1), 131–140. https://doi.org/10.1002/ajim.22671

Dumas, O., Wiley, A. S., Quinot, C., Varraso, R., Zock, J. P., Henneberger, P. K., Speizer, F. E., Le Moual, N., & Camargo, C. A. (2017). Occupational exposure to disinfectants and asthma control in US nurses. *European Respiratory Journal*, *50*(4). https://doi.org/10.1183/13993003.00237-2017

EL, B., JD, L., JA, N., Xu, M., Joo, T., NL, N., BE, S., PJ, S., & MG, F. (2020). Enveloped Virus Inactivation on Personal Protective Equipment by Exposure to Ozone. In *medRxiv : the preprint server for health sciences*. https://doi.org/10.1101/2020.05.23.20111435

Epelle, E. I., Macfarlane, A., Cusack, M., Burns, A., Okolie, J. A., Mackay, W., Rateb, M., & Yaseen, M. (2023). Ozone application in different industries: A review of recent developments. *Chemical Engineering Journal*, *454*, 140188. https://doi.org/https://doi.org/10.1016/j.cej.2022.140188

Erdem, Y., IK, A., A, A. Ç., Inal, S., Ugurer, E., Sivaz, O., HE, K., IE, G., Sekerlisoy, G., Vural, O., & Özkaya, E. (2020). The risk of hand eczema in healthcare workers during the COVID-19 pandemic: Do we need specific attention or prevention strategies? *Contact Dermatitis*, *83*(5), 422–423. https://doi.org/10.1111/cod.13632

Erfani, B., Vilela, L., Julander, A., & Schenk, L. (2023). Safety data sheets as an information pathway on hazards of occupationally used cleaning agents. *Regulatory Toxicology and Pharmacology*, *142*. https://doi.org/10.1016/j.yrtph.2023.105447

Estienney, M., Daval-Frerot, P., LS, A.-G., Piroth, L., Stabile, P., JY, G., Rouleau, R., A, de R., & Belliot, G. (2022). Use of a Hydrogen Peroxide Nebulizer for Viral Disinfection of Emergency Ambulance and Hospital Waiting Room. *Food and Environmental Virology*, *14*(2), 217–221. https://doi.org/10.1007/s12560-022-09519-y

Estrin, W. J., Cavalieri, S. A., Wald, P., Becker, C. E., Jones, J. R., & Cone, J. E. (1987). Evidence of Neurologic Dysfunction Related to Long-term Ethylene Oxide Exposure. *Archives of Neurology*, *44*(12), 1283–1286. https://doi.org/10.1001/ARCHNEUR.1987.00520240057012

F, D. S., Siriruttanapruk, S., JS, M., & PS, B. (1998). Occupational asthma due to glutaraldehyde. *Monaldi Archives for Chest Disease = Archivio Monaldi per Le Malattie Del Torace*, *53*(1), 50–55. https://pubmed.ncbi.nlm.nih.gov/9632908/

Faller, E. M., Bin Miskam, N., & Pereira, A. (2018). Exploratory study on occupational health hazards among health care workers in the Philippines. *Annals of Global Health*, *84*(3), 338–341. https://doi.org/10.29024/aogh.2316

Felemban, E. M., Youssef, H. A. M., & Thobaity, A. A. (2021). Factors affecting the decontamination process in hospitals in Saudi Arabia. *Risk Management and Healthcare Policy*, *14*, 357–363. https://doi.org/10.2147/RMHP.S295262

Fickenscher, M.-C., Stewart, M., Helber, R., Quilligan, E. J., Kreitenberg, A., Prietto, C. A., & Gardner, V. O. (2023). Operating room disinfection: operator-driven ultraviolet 'C' vs. chemical treatment. *Infection Prevention in Practice*, *5*(3), 100301. https://doi.org/https://doi.org/10.1016/j.infpip.2023.100301

Flyvholm, M.-A. (1993). Contact allergens in registered cleaning agents for industrial and household use. *British Journal of Industrial Medicine*, *50*(11), 1043–1050. https://doi.org/10.1136/oem.50.11.1043

Folletti, I., Siracusa, A., & Paolocci, G. (2017). Update on asthma and cleaning agents. *Current Opinion in Allergy and Clinical Immunology*, *17*(2), 90–95. https://doi.org/10.1097/ACI.0000000000000349

França, D., Sacadura-Leite, E., Fernandes-Almeida, C., & Filipe, P. (2019). Occupational dermatoses among healthcare workers in a hospital center in Portugal. *Revista Brasileira de Medicina Do Trabalho*, *17*(3), 285–291. https://doi.org/10.5327/Z1679443520190393

Frickmann, H., Bachert, S., Warnke, P., & Podbielski, A. (2018). Validated measurements of microbial loads on environmental surfaces in intensive care units before and after disinfecting cleaning. *Journal of Applied Microbiology*, *124*(3), 874–880. https://doi.org/10.1111/jam.13675

Gannon, P. F. G., Bright, P., Campbell, M., O'Hickey, S. P., & Sherwood Burge, P. (1995). Occupational asthma due to glutaraldehyde and formaldehyde in endoscopy and x ray departments. *Thorax*, *50*(2), 156–159. https://doi.org/10.1136/THX.50.2.156

García-Ávila, F., Valdiviezo-Gonzales, L., Cadme-Galabay, M., Gutiérrez-Ortega, H., Altamirano-Cárdenas, L., Arévalo, C. Z.-, & Flores del Pino, L. (2020). Considerations on water quality and the use of chlorine in times of SARS-CoV-2 (COVID-19) pandemic in the community. *Case Studies in Chemical and Environmental Engineering*, *2*, 100049. https://doi.org/https://doi.org/10.1016/j.cscee.2020.100049

Garrido, A. N., House, R., Lipszyc, J. C., Liss, G. M., Holness, D. L., & Tarlo, S. M. (2022). Cleaning agent usage in healthcare professionals and relationship to lung and skin symptoms. *Journal of Asthma*, *59*(4), 673–681. https://doi.org/10.1080/02770903.2021.1871740

Garvey, M. I., Wilkinson, M. A. C., Bradley, C. W., Holden, K. L., & Holden, E. (2018). Wiping out MRSA: Effect of introducing a universal disinfection wipe in a large UK teaching hospital. *Antimicrobial Resistance and Infection Control*, *7*(1). https://doi.org/10.1186/s13756-018-

0445-7

Gaskins, A. J., Chavarro, J. E., Rich-Edwards, J. W., Missmer, S. A., Laden, F., Henn, S. A., & Lawson, C. C. (2017). Occupational use of high-level disinfectants and fecundity among nurses. *Scandinavian Journal of Work, Environment and Health*, *43*(2), 171–180. https://doi.org/10.5271/SJWEH.3623

Gasparini, G., Carmisciano, L., Giberti, I., Murgioni, F., Parodi, A., & Gallo, R. (2020). Healthy Hands: a pilot study for the prevention of chronic hand eczema in healthcare workers of an Italian University Hospital. *Giornale Italiano Di Dermatologia e Venereologia : Organo Ufficiale, Societa Italiana Di Dermatologia e Sifilografia*, *155*(6), 760–763. https://doi.org/10.23736/S0392-0488.19.06220-5

Ge, T., Lu, Y., Zheng, S., Zhuo, L., Yu, L., Ni, Z., Zhou, Y., Ni, L., Qu, T., & Zhong, Z. (2021). Evaluation of disinfection procedures in a designated hospital for COVID-19. *American Journal of Infection Control*, *49*(4), 447–451. https://doi.org/10.1016/j.ajic.2020.08.028

Gerding, T., Wang, J., & Newman, N. (2023). Examining Work Stress and Air Pollutants Exposure of Home Healthcare Workers. *Atmosphere*, *14*(9). https://doi.org/10.3390/atmos14091393

Gerster, F. M., Vernez, D., Wild, P. P., & Hopf, N. B. (2014). Hazardous substances in frequently used professional cleaning products. *International Journal of Occupational and Environmental Health*, *20*(1), 46–60. https://doi.org/10.1179/2049396713Y.0000000052

Ghafoor, D., Khan, Z., Khan, A., Ualiyeva, D., & Zaman, N. (2021). Excessive use of disinfectants against COVID-19 posing a potential threat to living beings. *Current Research in Toxicology*, *2*, 159–168. https://doi.org/https://doi.org/10.1016/j.crtox.2021.02.008

Gharaibeh, A., Smith, R. H., & Conway, M. J. (2021). Reducing Spread of Infections with a Photocatalytic Reactor—Potential Applications in Control of Hospital Staphylococcus aureus and Clostridioides difficile Infections and Inactivation of RNA Viruses. *Infectious Disease Reports*, *13*(1), 58–71. https://doi.org/10.3390/IDR13010008

Ghelli, F., Cocchi, E., Bellisario, V., Buglisi, M., Squillacioti, G., Santovito, A., & Bono, R. (2022). The formation of SCEs as an effect of occupational exposure to formaldehyde. *Archives of Toxicology*, *96*(4), 1101–1108. https://doi.org/10.1007/s00204-022-03238-w

Gonzalez, M., Jégu, J., Kopferschmitt, M. C., Donnay, C., Hedelin, G., Matzinger, F., Velten, M., Guilloux, L., Cantineau, A., & de Blay, F.

(2014). Asthma among workers in healthcare settings: Role of disinfection with quaternary ammonium compounds. *Clinical and Experimental Allergy*, *44*(3), 393–406. https://doi.org/10.1111/cea.12215

Goodyear, N., Markkanen, P., Beato-Melendez, C., Mohamed, H., Gore, R., Galligan, C., Sama, S., & Quinn, M. (2018). Cleaning and disinfection in home care: A comparison of 2 commercial products with potentially different consequences for respiratory health. *American Journal of Infection Control*, *46*(4), 410–416. https://doi.org/https://doi.org/10.1016/j.ajic.2017.09.033

Gould, D. (1994). Nurses' hand decontamination practice: results of a local study. In *The Journal of hospital infection* (Vol. 28, Issue 1, pp. 15–30). https://doi.org/10.1016/0195-6701(94)90149-x

Graham, D., Lee, C., & Jordan, B. J. (2023). Dry Hydrogen Peroxide: one molecule for a One Health approach—a narrative review. *Journal of Public Health and Emergency*, *7*. https://doi.org/10.21037/jphe-22-105

Groenewold, M., Brown, L., Smith, E., Haring Sweeney, M., Pana-Cryan, R., & Schnorr, T. (2019). Burden of occupational morbidity from selected causes in the United States overall and by NORA industry sector, 2012: A conservative estimate. *American Journal of Industrial Medicine*, *62*(12), 1117–1134. https://doi.org/10.1002/ajim.23048

Gruszecka, J., Filip, R., & Gutkowska, D. (2021). The state of microbiological cleanliness of surfaces and equipment of an endoscopic examination laboratory—data from a reference tertiary clinical endoscopy center in southern poland. *International Journal of Environmental Research and Public Health*, *18*(12). https://doi.org/10.3390/ijerph18126346

GS, G. (1988). Decontamination: a microbiologist's perspective. *Journal of Healthcare Materiel Management*, *6*(1), 36–41. https://pubmed.ncbi.nlm.nih.gov/10285793/

Gupta, R. K., Micochova, P., Chadha, A., Hesseloj, T., Fraternali, F., & Ramsden, J. J. (2021). Rapid inactivation of SARS-CoV-2 by titanium dioxide surface coating. *Wellcome Open Research*, *6*. https://doi.org/10.12688/wellcomeopenres.16577.2

Gutterman, E., Jorgensen, L., Mitchell, A., & Fua, S. (2013). Adverse staff health outcomes associated with endoscope reprocessing. *Biomedical Instrumentation & Technology*, *47*(2), 172–179. https://doi.org/10.2345/0899-8205-47.2.172

HA, W. (1992). Glutaraldehyde allergy in hospital workers. In *Lancet (London, England)* (Vol. 339, Issue 8797, p. 880). https://doi.org/10.1016/0140-6736(92)90329-2

Hamnerius, N., Pontén, A., Bergendorff, O., Bruze, M., Björk, J., & Svedman, C. (2021). Skin exposures, hand eczema and facial skin disease in healthcare workers during the covid-19 pandemic: A cross-sectional study. *Acta Dermato-Venereologica*, *101*(9). https://doi.org/10.2340/00015555-3904

Hamnerius, N., Svedman, C., Bergendorff, O., Björk, J., Bruze, M., Engfeldt, M., & Pontén, A. (2018). Hand eczema and occupational contact allergies in healthcare workers with a focus on rubber additives. *Contact Dermatitis*, *79*(3), 149–156. https://doi.org/10.1111/cod.13042

Hamnerius, N., Svedman, C., Bergendorff, O., Björk, J., Bruze, M., & Pontén, A. (2018). Wet work exposure and hand eczema among healthcare workers: a cross-sectional study. *The British Journal of Dermatology*, *178*(2), 452–461. https://doi.org/10.1111/bjd.15813

Han, A. A., Buerger, A. N., Allen, H., Vincent, M., Thornton, S. A., Unice, K. M., Maier, A., & Quiñones-Rivera, A. (2022). Assessment of ethanol exposure from hand sanitizer use and potential for developmental toxicity in nursing infants. *Journal of Applied Toxicology*, *42*(9), 1424–1442. https://doi.org/10.1002/jat.4284

Han, J. H., Sullivan, N., Leas, B. F., Pegues, D. A., Kaczmarek, J. L., & Umscheid, C. A. (2015). Cleaning hospital room surfaces to prevent health care-associated infections: A technical brief. *Annals of Internal Medicine*, *163*(8), 598–607. https://doi.org/10.7326/M15-1192

Hatt, S., Schindler, B., Bach, D., & Greene, C. (2020). Washer disinfector and alkaline detergent efficacy against C. difficile on plastic bedpans. *American Journal of Infection Control*, *48*(7), 761–764. https://doi.org/https://doi.org/10.1016/j.ajic.2019.11.028

Haufroid, V., Merz, B., Hofmann, A., Tschopp, A., Lison, D., & Hotz, P. (2007). Exposure to ethylene oxide in hospitals: Biological monitoring and influence of glutathione S-transferase and epoxide hydrolase polymorphisms. *Cancer Epidemiology Biomarkers and Prevention*, *16*(4), 796–802. https://doi.org/10.1158/1055-9965.EPI-06-0915

Hautemanière, A., Cunat, L., Ahmed-Lecheheb, D., Hajjard, F., Gerardin, F., Morele, Y., & Hartemann, P. (2013). Assessment of exposure to ethanol vapors released during use of Alcohol-Based Hand Rubs by healthcare workers. *Journal of Infection and Public Health*, *6*(1), 16–26.

https://doi.org/https://doi.org/10.1016/j.jiph.2012.09.015

Hawley, B., Casey, M., Virji, M. A., Cummings, K. J., Johnson, A., & Cox-Ganser, J. (2018). Respiratory symptoms in hospital cleaning staff exposed to a product containing hydrogen peroxide, peracetic acid, and acetic acid. *Annals of Work Exposures and Health*, *62*(1), 28–40. https://doi.org/10.1093/ANNWEH/WXX087

Hawley, B., ML, C., JM, C.-G., Edwards, N., KB, F., & KJ, C. (2016). Notes from the Field: Respiratory Symptoms and Skin Irritation Among Hospital Workers Using a New Disinfection Product - Pennsylvania, 2015. *MMWR. Morbidity and Mortality Weekly Report*, *65*(15), 400–401. https://doi.org/10.15585/mmwr.mm6515a3

Heibati, B., Jaakkola, M. S., Lajunen, T. K., Ducatman, A., Veysi, R., Karimi, A., & Jaakkola, J. J. K. (2022). Do hospital workers experience a higher risk of respiratory symptoms and loss of lung function? *BMC Pulmonary Medicine*, *22*(1). https://doi.org/10.1186/s12890-022-02098-5

Held, E., Mygind, K., Wolff, C., Gyntelberg, F., & Agner, T. (2002). Prevention of work related skin problems: An intervention study in wet work employees. *Occupational and Environmental Medicine*, *59*(8), 556–561. https://doi.org/10.1136/oem.59.8.556

Hell, M., & Pauser, G. (2007). Disinfection for infection prevention over the course of time. *GMS Krankenhaushygiene Interdisziplinar*, *2*(1), Doc16. https://pubmed.ncbi.nlm.nih.gov/20200677/

Henn, S., Boiano, J. M., & Steege, A. L. (2015). Precautionary practices of healthcare workers who disinfect medical and dental devices using high-level disinfectants. *Infection Control and Hospital Epidemiology*, *36*(2), 180–185. https://doi.org/10.1017/ice.2014.37

Hetzmann, M. S., Mojtahedzadeh, N., Nienhaus, A., Harth, V., & Mache, S. (2021). Occupational health and safety measures in German outpatient care services during the covid-19 pandemic: A qualitative study. *International Journal of Environmental Research and Public Health*, *18*(6), 1–17. https://doi.org/10.3390/ijerph18062987

HG, T., TH, L., MA, B., JW, K., & Muniandy, P. (2023). Prevalence of Occupational Hand Eczema among Healthcare Workers and its Associated Risk Factors in a Tertiary Hospital in Sarawak During Covid-19 Pandemic. *Indian Journal of Dermatology*, *68*(1), 121.

https://doi.org/10.4103/ijd.ijd_803_22

HH, M. (1971). [Occupational dermatitis in medical personnel]. *Zeitschrift fur arztliche Fortbildung*, *65*(16), 825–830. https://pubmed.ncbi.nlm.nih.gov/4401721/

HH, M., Baatjies, R., Singh, T., & MF, J. (2021). Asthma Phenotypes and Host Risk Factors Associated With Various Asthma-Related Outcomes in Health Workers. *Frontiers in Allergy*, *2*, 747566. https://doi.org/10.3389/falgy.2021.747566

Hildre, T. T., Heiro, H., Sandven, I., & Hammarström, B. (2023). Ambient Environmental Ozone and Variation of Fractional Exhaled Nitric Oxide (FeNO) in Hairdressers and Healthcare Workers. *International Journal of Environmental Research and Public Health*, *20*(5). https://doi.org/10.3390/ijerph20054271

HK, V., RJ, B., SHL, L., Morken, T., OJ, M., & Irgens-Hansen, K. (2023). Changes in Infection Prevention Practices and Occurrence of Skin Symptoms among Healthcare Workers, Cleaners and Day-care Workers in Norway during the COVID-19 Pandemic. *Acta Dermato-Venereologica*, *103*, adv00840. https://doi.org/10.2340/actadv.v103.3420

Holtjer, J. C. S., Bloemsma, L. D., Beijers, R. J. H. C. G., Cornelissen, M. E. B., Hilvering, B., Houweling, L., Vermeulen, R. C. H., Downward, G. S., & der Zee, A.-H. M. (2023). Identifying risk factors for COPD and adult-onset asthma: an umbrella review. *European Respiratory Review*, *32*(168). https://doi.org/10.1183/16000617.0009-2023

Homyer, K. M., & Mehendale, F. V. (2023). Time to rethink medical disinfection from a planetary health perspective. *Journal of Global Health Reports*, *7*. https://doi.org/10.29392/001c.87862

Houben, E., K, D. P., & Rogiers, V. (2006). Skin condition associated with intensive use of alcoholic gels for hand disinfection: a combination of biophysical and sensorial data. *Contact Dermatitis*, *54*(5), 261–267. https://doi.org/10.1111/j.0105-1873.2006.00817.x

Huang, Y., Huo, S., Mo, J., & Huang, D. (2023). Highly Effective and Broad-Spectrum Antimicrobial Quaternary Ammonium Salts Containing Camphene Structure: Preparation, Surface-Active Properties, and Bioassay. *ACS Omega*. https://doi.org/10.1021/acsomega.3c03599

IA, K., MA, G., MI, R., GS, R., GM, A., & MIu, K. (1995). [Protection of medical personnel hands with Steriprest preparation]. In *Meditsina*

truda i promyshlennaia ekologiia (Issue 12, pp. 38–40). https://pubmed.ncbi.nlm.nih.gov/8673386/

Ilyas, S., Srivastava, R. R., & Kim, H. (2020). Disinfection technology and strategies for COVID-19 hospital and bio-medical waste management. *Science of the Total Environment*, *749*. https://doi.org/10.1016/j.scitotenv.2020.141652

Ishihara, M., Hata, Y., Hiruma, S., Takayama, T., Nakamura, S., Sato, Y., Ando, N., Fukuda, K., Murakami, K., & Yokoe, H. (2020). Safety of concentrated bioshell calcium oxidewater application for surface and skin disinfections against pathogenic microbes. *Molecules*, *25*(19). https://doi.org/10.3390/molecules25194502

Ituen, E., Akaranta, O., Singh, A., & Yuanhua, L. (2021). Overview of Spray Aerosols for Disinfection Against Airborne and Surface Spread of Covid-19. *Letters in Applied NanoBioScience*, *10*(3), 2494–2500. https://doi.org/10.33263/LIANBS103.24942500

JA, R., SR, S., Wernicki, P., RR, F., & Charlton, N. (2021). First Aid for Pool Chemical Exposure: A Narrative Review. *Cureus*, *13*(7), e16755. https://doi.org/10.7759/cureus.16755

Jacobsen, G., Rasmussen, K., Bregnhøj, A., Isaksson, M., Diepgen, T. L., & Carstensen, O. (2022). Causes of irritant contact dermatitis after occupational skin exposure: a systematic review. *International Archives of Occupational and Environmental Health*, *95*(1), 35–65. https://doi.org/10.1007/s00420-021-01781-0

Jann, J., Drevelle, O., Chen, X. G., Auclair-Gilbert, M., Soucy, G., Faucheux, N., & Fortier, L.-C. (2021). Rapid antibacterial activity of anodized aluminum-based materials impregnated with quaternary ammonium compounds for high-touch surfaces to limit transmission of pathogenic bacteria. *RSC Advances*, *11*(60), 38172–38188. https://doi.org/10.1039/d1ra07159a

JD, W., AY, L., MC, M., KE, F., AM, N., & RL, N. (2008). Occupational contact urticaria: Australian data. *The British Journal of Dermatology*, *159*(1), 125–131. https://doi.org/10.1111/j.1365-2133.2008.08583.x

JE, K., Lash, A., RM, B., Shore, M., & CE, B. (1990). Neuropsychologic “impairment” in a cohort of hospital workers chronically exposed to ethylene oxide. *Journal of Toxicology. Clinical Toxicology*, *28*(1), 21–28. https://doi.org/10.3109/15563659008993473

JM, L., Kanerva, M., Tarkka, E., Ollgren, J., & VJ, A. (2022). Low efficacy of three non-alcohol-based hand disinfectants utilizing silver polymer,

lactic acid and benzalkonium chloride on inactivation of bacteria on the fingertips of healthcare workers. *The Journal of Hospital Infection*, *125*, 55–59. https://doi.org/10.1016/j.jhin.2022.03.012

JM, M., & DN, W. (2016). Occupational Respiratory Allergic Diseases in Healthcare Workers. *Current Allergy and Asthma Reports*, *16*(11), 77. https://doi.org/10.1007/s11882-016-0657-y

Jonsdottir, H. R., Zysset, D., Lenz, N., Siegrist, D., Ruedin, Y., Ryter, S., Züst, R., Geissmann, Y., Ackermann-Gäumann, R., Engler, O. B., & Weber, B. (2023). Virucidal activity of three standard chemical disinfectants against Ebola virus suspended in tripartite soil and whole blood. *Scientific Reports*, *13*(1), 1–8. https://doi.org/10.1038/s41598-023-42376-8

JP, H., Allen, J., Brock, K., Falconer, J., MJ, H., GC, S., & Strohm, B. (1984). Normal sister chromatid exchange levels in hospital sterilization employees exposed to ethylene oxide. *Journal of Occupational Medicine. : Official Publication of the Industrial Medical Association*, *26*(1), 29–32. https://doi.org/10.1097/00043764-198401000-00007

JS, M., TJ, R., AS, H., Carayon, P., ML, Z., Schuetz, V., Reppen, M., Smith, W., Koffarnus, K., RL, B., Bowling, J., Jalali, K., & Safdar, N. (2020). Implementing daily chlorhexidine gluconate treatment for the prevention of healthcare-associated infections in non-intensive care settings: A multiple case analysis. *PloS One*, *15*(4), e0232062. https://doi.org/10.1371/journal.pone.0232062

JW, Y., CJ, H., & RC, S. (1983). Exposure to ethylene oxide at work increases sister chromatid exchanges in human peripheral lymphocytes. *Science (New York, N.Y.)*, *219*(4589), 1221–1223. https://doi.org/10.1126/science.6828851

KA, R., JD, S., Garavito, F., Anderson, B., & JM, I. (2021). Impact of a Whole-Room Atomizing Disinfection System on Healthcare Surface Contamination, Pathogen Transfer, and Labor Efficiency. *Critical Care Explorations*, *3*(2), e0340. https://doi.org/10.1097/CCE.0000000000000340

Kampf, G. (2008). What is left to justify the use of chlorhexidine in hand hygiene? *The Journal of Hospital Infection*, *70*, 27–34. https://doi.org/10.1016/S0195-6701(08)60008-0

Kampf, G., & Muscatiello, M. (2003). Dermal tolerance of Sterillium, a propanol-based hand rub. *The Journal of Hospital Infection*, *55*(4), 295–

298. https://doi.org/10.1016/j.jhin.2003.09.001

Kampf, G., Muscatiello, M., Häntschel, D., & Rudolf, M. (2002). Dermal tolerance and effect on skin hydration of a new ethanol-based hand gel. In *The Journal of hospital infection* (Vol. 52, Issue 4, pp. 297–301). https://doi.org/10.1053/jhin.2002.1311

Kampf, G., Reichel, M., Feil, Y., Eggerstedt, S., & PM, K. (2008). Influence of rub-in technique on required application time and hand coverage in hygienic hand disinfection. *BMC Infectious Diseases*, *8*, 149. https://doi.org/10.1186/1471-2334-8-149

Kampf, G., Todt, D., Pfaender, S., & Steinmann, E. (2020). Persistence of coronaviruses on inanimate surfaces and their inactivation with biocidal agents. *Journal of Hospital Infection*, *104*(3), 246–251. https://doi.org/10.1016/j.jhin.2020.01.022

Kathare, M., Julander, A., Erfani, B., & Schenk, L. (2022). An Overview of Cleaning Agents' Health Hazards and Occupational Injuries and Diseases Attributed to Them in Sweden. *Annals of Work Exposures and Health*, *66*(6), 741–753. https://doi.org/10.1093/annweh/wxac006

Keegel, T., & RL, N. (2018). Wet work and healthcare workers: use of hand disinfectants not associated with self-reported eczema. In *The British journal of dermatology* (Vol. 178, Issue 2, pp. 324–325). https://doi.org/10.1111/bjd.16166

Kieć-Swierczyńska, M. (1996). [Allergic reaction to merthiolate (a disinfectant) based on material from the Occupational Medicine Institute in Lodz]. In *Medycyna pracy* (Vol. 47, Issue 2, pp. 125–131). https://pubmed.ncbi.nlm.nih.gov/8656996/

Kieć-Swierczyńska, M., & Krecisz, B. (2000). Occupational skin diseases among the nurses in the region of Lódź. *International Journal of Occupational Medicine and Environmental Health*, *13*(3), 179–184. https://pubmed.ncbi.nlm.nih.gov/11109741/

Kiely, L. F., Moloney, E., O'Sullivan, G., Eustace, J. A., Gallagher, J., & Bourke, J. F. (2021). Irritant contact dermatitis in healthcare workers as a result of the COVID-19 pandemic: a cross-sectional study. *Clinical and Experimental Dermatology*, *46*(1), 142–144. https://doi.org/10.1111/ced.14397

Kirman, C. R., Li, A. A., Sheehan, P. J., Bus, J. S., Lewis, R. C., & Hays, S. M. (2021). Ethylene oxide review: characterization of total exposure via endogenous and exogenous pathways and their implications to risk assessment and risk management. *Journal of Toxicology and Environmental Health - Part B: Critical Reviews*, *24*(1), 1–29. https://doi.org/10.1080/10937404.2020.1852988

KM, C. (2015). A survey of occupational skin disease in UK health care workers. *Occupational Medicine (Oxford, England)*, *65*(1), 29–31. https://doi.org/10.1093/occmed/kqu170

KM, O., & Farooque, S. (2014). Chlorhexidine: an unrecognised cause of anaphylaxis. *Postgraduate Medical Journal*, *90*(1070), 709–714. https://doi.org/10.1136/postgradmedj-2013-132291

Kobos, L., Anderson, K., Kurth, L., Liang, X., Groth, C. P., England, L., Laney, A. S., & Virji, M. A. (2022). Characterization of Cleaning and Disinfection Product Use, Glove Use, and Skin Disorders by Healthcare Occupations in a Midwestern Healthcare Facility. *Buildings*, *12*(12). https://doi.org/10.3390/BUILDINGS12122216

Konno, A., Okubo, T., Enoeda, Y., Uno, T., Sato, T., Yokota, S.-I., Yano, R., & Yamaguchi, H. (2023). Human pathogenic bacteria on high-touch dry surfaces can be controlled by warming to human-skin temperature under moderate humidity. *PLoS ONE*, *18*(9). https://doi.org/10.1371/journal.pone.0291765

Kovach, C. R., Taneli, Y., Neiman, T., Dyer, E. M., Arzaga, A. J. A., & Kelber, S. T. (2017). Evaluation of an ultraviolet room disinfection protocol to decrease nursing home microbial burden, infection and hospitalization rates. *BMC Infectious Diseases*, *17*(1). https://doi.org/10.1186/s12879-017-2275-2

Krause, M., & Dolák, F. (2021). Antibacterial treatment of selected high-touch objects and surfaces within provision of nursing care in terms of prevention of healthcare-associated infections. *Healthcare (Switzerland)*, *9*(6). https://doi.org/10.3390/healthcare9060675

Kruszewska, E., Czupryna, P., Pancewicz, S., Martonik, D., Bukłaha, A., & Moniuszko-Malinowska, A. (2022). Is Peracetic Acid Fumigation Effective in Public Transportation? *International Journal of Environmental Research and Public Health*, *19*(5). https://doi.org/10.3390/ijerph19052526

KS, I., GB, J., & Agner, T. (2012). Exposures related to hand eczema: a study of healthcare workers. *Contact Dermatitis*, *66*(5), 247–253. https://doi.org/10.1111/j.1600-0536.2011.02027.x

KS, I., GB, J., LH, G., & Agner, T. (2016). Prevalence of delayed-type and immediate-type hypersensitivity in healthcare workers with hand

eczema. *Contact Dermatitis*, *75*(4), 223–229. https://doi.org/10.1111/cod.12587

KS, I., GB, J., TL, D., Gluud, C., J, L. H., Winkel, P., SF, T., & Agner, T. (2012). Skin care education and individual counselling versus treatment as usual in healthcare workers with hand eczema: randomised clinical trial. *BMJ (Clinical Research Ed.)*, *345*, e7822. https://doi.org/10.1136/bmj.e7822

Kundrapu, S., Sunkesula, V., LA, J., BM, S., & CJ, D. (2012). Daily disinfection of high-touch surfaces in isolation rooms to reduce contamination of healthcare workers' hands. *Infection Control and Hospital Epidemiology*, *33*(10), 1039–1042. https://doi.org/10.1086/667730

Kurth, L., Virji, M. A., Storey, E., Framberg, S., Kallio, C., Fink, J., & Laney, A. S. (2017). Current asthma and asthma-like symptoms among workers at a Veterans Administration Medical Center. *International Journal of Hygiene and Environmental Health*, *220*(8), 1325–1332. https://doi.org/https://doi.org/10.1016/j.ijheh.2017.09.001

Laborde-Castérot, H., Villa, A. F., Rosenberg, N., Dupont, P., Lee, H. M., & Garnier, R. (2012). Occupational rhinitis and asthma due to EDTA-containing detergents or disinfectants. *American Journal of Industrial Medicine*, *55*(8), 677–682. https://doi.org/10.1002/AJIM.22036

Laditka, J. N., Laditka, S. B., Arif, A. A., & Hoyle, J. N. (2020). Work-related asthma in the USA: Nationally representative estimates with extended follow-up. *Occupational and Environmental Medicine*, *77*(9), 617–622. https://doi.org/10.1136/oemed-2019-106121

Larese Filon, F., Pesce, M., Paulo, M. S., Loney, T., Modenese, A., John, S. M., Kezic, S., & Macan, J. (2021). Incidence of occupational contact dermatitis in healthcare workers: a systematic review. *Journal of the European Academy of Dermatology and Venereology*, *35*(6), 1285–1289. https://doi.org/10.1111/JDV.17096

Larner, J., Durrant, A., Hughes, P., Mahalingam, D., Rivers, S., Matar, H., Thomas, E., Barrett, M., Pinhal, A., Amer, N., Hall, C., Jackson, T., Catalani, V., & Chilcott, R. P. (2020). Efficacy of Different Hair and Skin Decontamination Strategies with Identification of Associated Hazards to First Responders. *Prehospital Emergency Care*, *24*(3), 355–368. https://doi.org/10.1080/10903127.2019.1636912

Lee, S., AC, P., MJ, S., & M, van T. (2021). Insufficient respiratory hazard identification in the safety data sheets for cleaning and disinfection

products used in healthcare organisations across England and Wales. *Occupational and Environmental Medicine*, *78*(4), 293–295. https://doi.org/10.1136/oemed-2020-106881

Lee, S. J., Nam, B., Harrison, R., & Hong, O. (2014). Acute symptoms associated with chemical exposures and safe work practices among hospital and campus cleaning workers: A pilot study. *American Journal of Industrial Medicine*, *57*(11), 1216–1226. https://doi.org/10.1002/AJIM.22376

Lei, H., Jones, R. M., & Li, Y. (2017). Exploring surface cleaning strategies in hospital to prevent contact transmission of methicillin-resistant Staphylococcus aureus. *BMC Infectious Diseases*, *17*(1). https://doi.org/10.1186/s12879-016-2120-z

Leinster, P., Baum, J. M., & Baxter, P. J. (1993). An assessment of exposure to glutaraldehyde in hospitals: Typical exposure levels and recommended control measures. *British Journal of Industrial Medicine*, *50*(2), 107–111. https://doi.org/10.1136/oem.50.2.107

Lemire, P., Chevallier, E., Lyon-Caen, S., Sévin, E., Boudier, A., Da Silva, E. P., De Thuin, C., Slama, R., Dumas, O., Siroux, V., Le Moual, N., Eyriey, E., Licinia, A., Vellement, A., Pin, I., Hoffmann, P., Hullo, E., Llerena, C., Morin, X., … Slama, R. (2022). Association between household cleaning product profiles evaluated by the Ménag'Score® index and asthma symptoms among women from the SEPAGES cohort. *International Archives of Occupational and Environmental Health*. https://doi.org/10.1007/s00420-022-01860-w

Li, R. W. H., Lipszyc, J. C., Prasad, S., & Tarlo, S. M. (2018). Work-related asthma from cleaning agents versus other agents. *Occupational Medicine*, *68*(9), 587–592. https://doi.org/10.1093/OCCMED/KQY137

Lin, N., Rosemberg, M.-A., Li, W., Meza-Wilson, E., Godwin, C., & Batterman, S. (2021). Occupational exposure and health risks of volatile organic compounds of hotel housekeepers: Field measurements of exposure and health risks. *Indoor Air*, *31*(1), 26–39. https://doi.org/10.1111/ina.12709

Liss, G. M., Tarlo, S. M., Doherty, J., Purdham, J., Greene, J., McCaskell, L., & Kerr, M. (2003). Physician diagnosed asthma, respiratory symptoms, and associations with workplace tasks among radiographers in Ontario, Canada. *Occupational and Environmental Medicine*, *60*(4), 254–261. https://doi.org/10.1136/oem.60.4.254

Lodola, L., Bernardini, G., Riva, A., & Marraccini, P. (2000). [Evaluation and quantification of biologic risk in the hospital setting with ATP determination on work surfaces]. In *Giornale italiano di medicina del lavoro ed ergonomia* (Vol. 22, Issue 1, pp. 7–13). https://pubmed.ncbi.nlm.nih.gov/10771752/

Lu, Y., Cai, R., Xie, Z., Gao, X., Huang, X., Xu, J., Li, Y., & Hu, G. (2022). Current Status and Factors Associated with Clean Operating Rooms: A Survey of Hospitals in China. *Journal of Healthcare Engineering*, *2022*. https://doi.org/10.1155/2022/8749785

Lu, Y., Li, S., Xu, W., & Wang, Y. (2023). Numerical Simulation Study of Indoor Disinfection Spray Distribution based on CFD-DPM Method. *Journal of Engineering Research*. https://doi.org/https://doi.org/10.1016/j.jer.2023.10.039

Luchini, K., SNB, S., Mauro, R., Sargsyan, A., Newman, A., Persaud, P., Hawkins, D., Wolff, D., Staudinger, J., & BA, C. (2021). Sterilization and sanitizing of 3D-printed personal protective equipment using polypropylene and a Single Wall design. *3D Printing in Medicine*, *7*(1), 16. https://doi.org/10.1186/s41205-021-00106-8

MA, da S., PB, G., & SH, M. (1997). [Perception of health professionals of the risk of chemical exposure]. In *Revista brasileira de enfermagem* (Vol. 50, Issue 4, pp. 591–598). https://doi.org/10.1590/s0034-71671997000400013

MA, G. J., A, M. H., JC, A. G., M, L. A., L, M. O., P, T. M., & X, E. O. (2013). Exposure of health workers in primary health care to glutaraldehyde. *Journal of Occupational Medicine and Toxicology (London, England)*, *8*(1), 31. https://doi.org/10.1186/1745-6673-8-31

Mac Hovcová, A., Fenclová, Z., & Pelclová, D. (2013). Occupational skin diseases in Czech healthcare workers from 1997 to 2009. *International Archives of Occupational and Environmental Health*, *86*(3), 289–294. https://doi.org/10.1007/S00420-012-0764-6/FIGURES/2

Maier, A., Ovesen, J. L., Allen, C. L., York, R. G., Gadagbui, B. K., Kirman, C. R., Poet, T., & Quiñones-Rivera, A. (2015). Safety assessment for ethanol-based topical antiseptic use by health care workers: Evaluation of developmental toxicity potential. *Regulatory Toxicology and Pharmacology*, *73*(1), 248–264. https://doi.org/10.1016/j.yrtph.2015.07.015

Małaszuk, J., Andrzejak, R., Przybylski, M., Jurga, M., Szymańska, A., & Leszczyszyn, J. (2000). [Preliminary assessment of occupational exposure to Glutaraldehyde in selected endoscopic workplaces]. In *Medycyna pracy* (Vol. 51, Issue 4, pp. 365–371).

https://pubmed.ncbi.nlm.nih.gov/11059410/

Mallakpour, S., Azadi, E., & Hussain, C. M. (2021). Protection, disinfection, and immunization for healthcare during the COVID-19 pandemic: Role of natural and synthetic macromolecules. *Science of the Total Environment*, *776*. https://doi.org/10.1016/j.scitotenv.2021.145989

Manyele, S. V, Ngonyani, H. A., & Eliakimu, E. (2008). The status of occupational safety among health service providers in hospitals in Tanzania. *Tanzania Journal of Health Research*, *10*(3), 159–165. https://doi.org/10.4314/thrb.v10i3.14356

Marena, C., Lodola, L., Zecca, M., Bulgheroni, A., Carretto, E., Maserati, R., & Zambianchi, L. (2002). Assessment of handwashing practices with chemical and microbiologic methods: preliminary results from a prospective crossover study. In *American journal of infection control* (Vol. 30, Issue 6, pp. 334–340). https://doi.org/10.1067/mic.2002.125809

Matulonga, B., Rava, M., Siroux, V., Bernard, A., Dumas, O., Pin, I., Zock, J.-P., Nadif, R., Leynaert, B., & Le Moual, N. (2016). Women using bleach for home cleaning are at increased risk of non-allergic asthma. *Respiratory Medicine*, *117*, 264–271. https://doi.org/10.1016/j.rmed.2016.06.019

McKinley, L., CC, G., Balkenende, E., Clore, G., SS, H., Bartel, R., Bradley, S., Judd, J., Lyons, G., Rock, C., Rubin, M., Shaughnessy, C., HS, R., Perencevich, E., & Safdar, N. (2023). Evaluation of daily environmental cleaning and disinfection practices in veterans affairs acute and long-term care facilities: A mixed methods study. *American Journal of Infection Control*, *51*(2), 205–213. https://doi.org/10.1016/j.ajic.2022.05.014

Mehtar, S., Bulabula, A. N. H., Nyandemoh, H., & Jambawai, S. (2016). Deliberate exposure of humans to chlorine-the aftermath of Ebola in West Africa. *Antimicrobial Resistance and Infection Control*, *5*(1). https://doi.org/10.1186/S13756-016-0144-1

Melgar, M., Ramirez, M., Chang, A., & Antillon, F. (2022). Impact of dry hydrogen peroxide on hospital-acquired infection at a pediatric oncology hospital. *WHO Special Issue: Personal Protective Equipment Research and Innovation in the Context of the World Health Organization COVID-19 R&D Blueprint Program*, *50*(8), 909–915. https://doi.org/https://doi.org/10.1016/j.ajic.2021.12.010

Mirabelli, M. C., Zock, J.-P., Plana, E., Antó, J. M., Benke, G., Blanc, P. D., Dahlman-Höglund, A., Jarvis, D. L., Kromhout, H., Lillienberg, L.,

Norbäck, D., Olivieri, M., Radon, K., Sunyer, J., Torén, K., Van Sprundel, M., Villani, S., & Kogevinas, M. (2007). Occupational risk factors for asthma among nurses and related healthcare professionals in an international study. *Occupational and Environmental Medicine*, *64*(7), 474–479. https://doi.org/10.1136/oem.2006.031203

Moccia, G., Motta, O., Pironti, C., Proto, A., Capunzo, M., & De Caro, F. (2020). An alternative approach for the decontamination of hospital settings. *Journal of Infection and Public Health*, *13*(12), 2038–2044. https://doi.org/10.1016/j.jiph.2020.09.020

Mohite, V. S., Darade, M. M., Sharma, R. K., & Pawar, S. H. (2022). Nanoparticle Engineered Photocatalytic Paints: A Roadmap to Self-Sterilizing against the Spread of Communicable Diseases. *Catalysts*, *12*(3). https://doi.org/10.3390/catal12030326

Mohtar, N., Gazzali, A. M., Parumasivam, T., Hanafiah, N. H. M., & Yee, N. S. (2021). Proof of concept: The effectiveness of disinfectant tunnel as potential measure against COVID-19. *Sains Malaysiana*, *50*(7), 2135–2140. https://doi.org/10.17576/jsm-2021-5007-26

Molin, S., Bauer, A., Schnuch, A., & Geier, J. (2015). Occupational contact allergy in nurses: results from the Information Network of Departments of Dermatology 2003-2012. *Contact Dermatitis*, *72*(3), 164–171. https://doi.org/10.1111/cod.12330

MS, S., TA, G., & SM, M. (2006). Efficacy of an alcohol-based healthcare hand rub containing synergistic combination of farnesol and benzethonium chloride. In *International journal of hygiene and environmental health* (Vol. 209, Issue 5, pp. 477–487). https://doi.org/10.1016/j.ijheh.2006.04.006

Mungan, D., Özmen, İ., Evyapan, F., Topçu, F., Akgün, M., Arbak, P., & Bülbül, Y. (2019). Work-related symptoms of patients with asthma: A multicenter study. *Turkish Thoracic Journal*, *20*(4), 241–247. https://doi.org/10.5152/TurkThoracJ.2018.18123

Munyendo, W. L. L., & Kiprop, A. K. (2016). Design, preparation and evaluation of germicidal Toddalia asiatica herbal antiseptic detergent. *Journal of Applied Pharmaceutical Science*, *6*(11), 100–104. https://doi.org/10.7324/JAPS.2016.601116

MV, T., Jarboe, G., & RQ, F. (2008). Infection control in the dental office. *Dental Clinics of North America*, *52*(3), 609–628, x. https://doi.org/10.1016/j.cden.2008.02.002

Mwanga, H. H., Baatjies, R., & Jeebhay, M. F. (2022). Characterization of Exposure to Cleaning Agents Among Health Workers in Two Southern

African Tertiary Hospitals. *Annals of Work Exposures and Health*, *66*(8), 998–1009. https://doi.org/10.1093/annweh/wxac034

Mwanga, H. H., Baatjies, R., & Jeebhay, M. F. (2023). Occupational risk factors and exposure-response relationships for airway disease among health workers exposed to cleaning agents in tertiary hospitals. *Occupational and Environmental Medicine*. https://doi.org/10.1136/OEMED-2022-108763

Nakamura, S., Ishihara, M., Sato, Y., Takayama, T., Hiruma, S., Ando, N., Fukuda, K., Murakami, K., & Yokoe, H. (2020). Concentrated bioshell calcium oxide (BiSCaO) water kills pathogenic microbes: Characterization and activity. *Molecules*, *25*(13). https://doi.org/10.3390/molecules25133001

Navarathna, T., Jinadatha, C., Corona, B. A., Coppin, J. D., Choi, H., Bennett, M. R., Ghamande, G. S., Williams, M. D., Keene, R. E., & Chatterjee, P. (2023). Efficacy of a filtered far-UVC handheld disinfection device in reducing the microbial bioburden of hospital surfaces. *American Journal of Infection Control*. https://doi.org/10.1016/j.ajic.2023.05.003

Nayebzadeh, A. (2007). The effect of work practices on personal exposure to glutaraldehyde among health care workers. *Industrial Health*, *45*(2), 289–295. https://doi.org/10.2486/INDHEALTH.45.289

Ndlela, N. H., & Naidoo, R. N. (2023). Job and exposure intensity among hospital cleaning staff adversely affects respiratory health. *American Journal of Industrial Medicine*, *66*(3), 252–264. https://doi.org/10.1002/AJIM.23456

Nemli, A., Gümüş, K., & Başer, M. (2021). Ergoophthalmological risks associated with dry eye in the operating room. *Journal of Perioperative Nursing*, *34*(2), e-22. https://doi.org/10.26550/2209-1092.1115

Nettis, E., Colanardi, M. C., Soccio, A. L., Ferrannini, A., & Tursi, A. (2002). Occupational irritant and allergic contact dermatitis among healthcare workers. *Contact Dermatitis*, *46*(2), 101–107. https://doi.org/10.1034/J.1600-0536.2002.460208.X

Neves, P. R. F., Oliveira, T. D., Magalhães, T. F., Dos Reis, P. R. S., Tofaneli, L. A., Santos, A. A. B., Souza Machad, B., Oliveira, F. O., Da Silva Andrade, L. P. C., Badaro, R., & Mascarenhas, L. A. B. (2021). Numerical and experimental analyses for the improvement of surface instant decontamination technology through biocidal agent dispersion: Potential of application during pandemic. *PLoS ONE*, *16*(5).

https://doi.org/10.1371/journal.pone.0251817

Nguyen, K., Bui, D., Hashemi, M., Hocking, D. M., Mendis, P., Strugnell, R. A., & Dharmage, S. C. (2021). The potential use of hypochlorous acid and a smart prefabricated sanitising chamber to reduce occupation-related COVID-19 exposure. *Risk Management and Healthcare Policy*, *14*, 247–252. https://doi.org/10.2147/RMHP.S284897

Nielsen, J., & Bach, E. (1999). Work-related eye symptoms and respiratory symptoms in female cleaners. *Occupational Medicine*, *49*(5), 291–297. https://doi.org/10.1093/occmed/49.5.291

Norbäck, D. (1988). Skin and respiratory symptoms from exposure to alkaline glutaraldehyde in medical services. *Scandinavian Journal of Work, Environment & Health*, *14*(6), 366–71. https://www.jstor.org/stable/40965592

Obed, P., Amritanand, A., Antipas, O., Rebekah, G., Kirupakaran, H., Alex, R., & Paul, P. (2021). Acute work-related hazardous eye exposures in a health care environment - An observational study from a tertiary care hospital in South India. *Indian Journal of Ophthalmology*, *69*(12), 3532–3537. https://doi.org/10.4103/ijo.IJO_912_21

Ofstead, C. L., Hopkins, K. M., Daniels, F. E., Smart, A. G., & Wetzler, H. P. (2022). Splash generation and droplet dispersal in a well-designed, centralized high-level disinfection unit. *American Journal of Infection Control*, *50*(11), 1200–1207. https://doi.org/https://doi.org/10.1016/j.ajic.2022.08.016

Oie, S., Obayashi, A., Yamasaki, H., Furukawa, H., Kenri, T., Takahashi, M., Kawamoto, K., & Makino, S.-I. (2011). Disinfection methods for spores of Bacillus atrophaeus, B. anthracis, Clostridium tetani, C. botulinum and C. difficile. *Biological and Pharmaceutical Bulletin*, *34*(8), 1325–1329. https://doi.org/10.1248/bpb.34.1325

Okazaki, K., Fujino, Y., & Morikawa, Y. (2022). Effects of ultra-pure soft water on the hands of nurses in a neonatal intensive care unit: A randomized crossover study. *Contact Dermatitis*, *87*(6), 521–527. https://doi.org/10.1111/cod.14177

Okeke, C. A. V, Khanna, R., & Ehrlich, A. (2023). Quaternary Ammonium Compounds and Contact Dermatitis: A Review and Considerations During the COVID-19 Pandemic. *Clinical, Cosmetic and Investigational Dermatology*, *16*, 1721–1728.

https://doi.org/10.2147/CCID.S410910

Omrane, A., Khedher, A., Harrathi, C., Maoua, M., Khalfallah, T., Bouzgarrou, L., Mrizak, N., MA, H., & HBH, A. (2022). Quality of Life of Healthcare Workers Suffering from Occupational Contact Dermatitis. *Recent Advances in Inflammation & Allergy Drug Discovery*, *15*(1), 44–51. https://doi.org/10.2174/1872213X14666210303155135

Ortí-Lucas, R. M., & Muñoz-Miguel, J. (2017). Effectiveness of surface coatings containing silver ions in bacterial decontamination in a recovery unit. *Antimicrobial Resistance and Infection Control*, *6*(1). https://doi.org/10.1186/s13756-017-0217-9

Otterspoor, S., & Farrell, J. (2019). An evaluation of buffered peracetic acid as an alternative to chlorine and hydrogen peroxide based disinfectants. *Infection, Disease & Health*, *24*(4), 240–243. https://doi.org/10.1016/J.IDH.2019.06.003

Oza, H. H., Lee, M. G., Boisson, S., Pega, F., Medlicott, K., & Clasen, T. (2022). Occupational health outcomes among sanitation workers: A systematic review and meta-analysis. *International Journal of Hygiene and Environmental Health*, *240*, 113907. https://doi.org/https://doi.org/10.1016/j.ijheh.2021.113907

PA, S., Boeniger, M., JT, W., SE, S., MA, P., DK, G., JP, W., Garza, A., Froelich, R., Strauss, G., & al., et. (1992). Biologic markers in hospital workers exposed to low levels of ethylene oxide. *Mutation Research*, *278*(4), 237–251. https://doi.org/10.1016/s0165-1218(10)80003-5

Pałczyński, C., Walusiak, J., Ruta, U., & Górski, P. (2001). Occupational asthma and rhinitis due to glutaraldehyde: changes in nasal lavage fluid after specific inhalatory challenge test. In *Allergy* (Vol. 56, Issue 12, pp. 1186–1191). https://doi.org/10.1034/j.1398-9995.2001.00236.x

Parry, M. F., Sestovic, M., Renz, C., Pangan, A., Grant, B., & Shah, A. K. (2022). Environmental cleaning and disinfection: Sustaining changed practice and improving quality in the community hospital. *Antimicrobial Stewardship and Healthcare Epidemiology*, *2*(1). https://doi.org/10.1017/ash.2022.257

Patel, J., Gimeno Ruiz de Porras, D., Mitchell, L. E., Carson, A., Whitehead, L. W., Han, I., Pompeii, L., Conway, S., Zock, J.-P., Henneberger, P. K., Patel, R., De Los Reyes, J., & Delclos, G. L. (2023). Cleaning Tasks and Products and Asthma Among Healthcare Professionals. *Journal of Occupational & Environmental Medicine*. https://doi.org/10.1097/JOM.0000000000002990

PE, H., BA, R., & LC, L. (1990). Ethylene oxide. An occupational health hazard for hospital workers. *AORN Journal*, *51*(2), 480-481,483,485-486. https://doi.org/10.1016/s0001-2092(07)66079-7

Pechter, E., Davis, L. K., Tumpowsky, C., Flattery, J., Harrison, R., Reinisch, F., Reilly, M. J., Rosenman, K. D., Schill, D. P., Valiante, D., & Filios, M. (2005). Work-related asthma among health care workers: Surveillance data from California, Massachusetts, Michigan, and New Jersey, 1993-1997. *American Journal of Industrial Medicine*, *47*(3), 265–275. https://doi.org/10.1002/ajim.20138

Pemberton, M. A., & Kimber, I. (2021). Classification of chemicals as respiratory allergens based on human data: Requirements and practical considerations. *Regulatory Toxicology and Pharmacology*, *123*. https://doi.org/10.1016/j.yrtph.2021.104925

Peters, A., Cave, C., Carry, J., Sauser, J., & Pittet, D. (2022). Tolerability and acceptability of three alcohol-based hand-rub gel formulations: a randomized crossover study. *The Journal of Hospital Infection*, *123*, 112–118. https://doi.org/10.1016/j.jhin.2022.01.019

Petti, S., Messano, G. A., Polimeni, A., & Dancer, S. J. (2013). Effect of cleaning and disinfection on naturally contaminated clinical contact surfaces. *Acta Stomatologica Naissi*, *29*(67), 1265–1272. https://doi.org/10.5937/asn1367265P

Peyneau, M., de Chaisemartin, L., Gigant, N., Chollet-Martin, S., & Kerdine-Römer, S. (2022). Quaternary ammonium compounds in hypersensitivity reactions. *Frontiers in Toxicology*, *4*. https://doi.org/10.3389/ftox.2022.973680

Pironti, C., Motta, O., & Proto, A. (2021). Development of a new vapour phase methodology for textiles disinfection. *Cleaner Engineering and Technology*, *4*. https://doi.org/10.1016/j.clet.2021.100170

Polecka, A., Awchimkow, A., Owsianko, N., Baran, A., Hermanowicz, J. M., & Flisiak, I. (2023). Hand Eczema in the Polish Female Population. *Journal of Clinical Medicine*, *12*(18). https://doi.org/10.3390/jcm12186102

Polivka, B. J., Huntington-Moskos, L., Folz, R., & Barnett, R. (2022). CE: Environments & Health: Chemicals in the Home That Can Exacerbate Asthma. *American Journal of Nursing*, *122*(5), 34–39. https://doi.org/10.1097/01.NAJ.0000829776.73698.e0

Pontes, F., Pontes, H., Adachi, P., Rodini, C., Almeida, D., & Jr, P. D. (2008). Gingival and bone necrosis caused by accidental sodium hypochlorite injection instead of anaesthetic solution. In *International endodontic journal* (Vol. 41, Issue 3, pp. 267–270).

https://doi.org/10.1111/j.1365-2591.2007.01340.x

Popin, E., MC, K.-K., Gonzalez, M., Brom, M., Flesch, F., & Pauli, G. (2008). [The Incidence of occupational asthma in Alsace from 2001 to 2002. Results of intensification of the ONAP project in Alsace (2001-2002). Regional specificities]. In *Revue des maladies respiratoires* (Vol. 25, Issue 7, pp. 806–813). https://doi.org/10.1016/s0761-8425(08)74345-6

Popp, W., Vahrenholz, C., Przygoda, H., Brauksiepe, A., Goch, S., Müller, G., Schell, C., & Norpoth, K. (1994). DNA-protein cross-links and sister chromatid exchange frequencies in lymphocytes and hydroxyethyl mercapturic acid in urine of ethylene oxide-exposed hospital workers. *International Archives of Occupational and Environmental Health*, *66*(5), 325–332. https://doi.org/10.1007/BF00378365

Prodi, A., Rui, F., AB, F., MT, C., & FL, F. (2016). Healthcare workers and skin sensitization: north-eastern Italian database. *Occupational Medicine (Oxford, England)*, *66*(1), 72–74. https://doi.org/10.1093/occmed/kqv139

Prodi, A., Rui, F., Fortina, A. B., Corradin, M. T., & Filon, F. L. (2016). Healthcare workers and skin sensitization: North-eastern Italian database. *Occupational Medicine*, *66*(1), 72–74. https://doi.org/10.1093/occmed/kqv139

Purwar, T., Dey, S., Al-Kayyali, O. Z. A., Zalar, A. F., Doosttalab, A., Castillo, L., & Castano, V. M. (2022). Electrostatic Spray Disinfection Using Nano-Engineered Solution on Frequently Touched Surfaces in Indoor and Outdoor Environments. *International Journal of Environmental Research and Public Health*, *19*(12). https://doi.org/10.3390/ijerph19127241

Quinn, M. M., Henneberger, P. K., Braun, B., Delclos, G. L., Fagan, K., Huang, V., Knaack, J. L. S., Kusek, L., Lee, S.-J., Le Moual, N., Maher, K. A. E., McCrone, S. H., Mitchell, A. H., Pechter, E., Rosenman, K., Sehulster, L., Stephens, A. C., Wilburn, S., & Zock, J.-P. (2015). Cleaning and disinfecting environmental surfaces in health care: Toward an integrated framework for infection and occupational illness prevention. *American Journal of Infection Control*, *43*(5), 424–434. https://doi.org/https://doi.org/10.1016/j.ajic.2015.01.029

Quinot, C., Amsellem-Dubourget, S., Temam, S., Sevin, E., Barreto, C., Tackin, A., Félicité, J., Lyon-Caen, S., Siroux, V., Girard, R., Descatha, A., N, L. M., & Dumas, O. (2018). Development of a bar code-based exposure assessment method to evaluate occupational exposure to disinfectants and cleaning products: a pilot study. In *Occupational and environmental medicine* (Vol. 75, Issue 9, pp. 668–674). https://doi.org/10.1136/oemed-2017-104793

Quinot, C., Dumas, O., Henneberger, P. K., Varraso, R., Wiley, A. S., Speizer, F. E., Goldberg, M., Zock, J. P., Camargo, C. A., & Le Moual, N. (2017). Development of a job-task-exposure matrix to assess occupational exposure to disinfectants among US nurses. *Occupational and Environmental Medicine*, *74*(2), 130–137. https://doi.org/10.1136/oemed-2016-103606

Rai, R., El-Zaemey, S., Dorji, N., & Fritschi, L. (2020a). Occupational exposures to hazardous chemicals and agents among healthcare workers in Bhutan. *American Journal of Industrial Medicine*, *63*(12), 1109–1115. https://doi.org/10.1002/ajim.23192

Rai, R., El-Zaemey, S., Dorji, N., & Fritschi, L. (2020b). Reliability and validity of an adapted questionnaire assessing occupational exposures to hazardous chemicals among health care workers in Bhutan. *International Journal of Occupational and Environmental Medicine*, *11*(3), 128–139. https://doi.org/10.34172/ijoem.2020.1878

Rangel, K., Cabral, F. O., Lechuga, G. C., Villas-Bôas, M. H. S., Midlej, V., & De-Simone, S. G. (2022). Effectiveness Evaluation of a UV-C-Photoinactivator against Selected ESKAPE-E Pathogens. *International Journal of Environmental Research and Public Health*, *19*(24). https://doi.org/10.3390/ijerph192416559

Rava, M., Ahmed, I., Kogevinas, M., Le Moual, N., Bouzigon, E., Curjuric, I., Dizier, M.-H., Dumas, O., Gonzalez, J. R., Imboden, M., Mehta, A. J., Tubert-Bitter, P., Zock, J.-P., Jarvis, D., Probst-Hensch, N. M., Demenais, F., & Nadif, R. (2017). Genes interacting with occupational exposures to low molecular weight agents and irritants on adult-onset asthma in three European studies. *Environmental Health Perspectives*, *125*(2), 207–214. https://doi.org/10.1289/EHP376

Razali, A., FI, A., & AM, K. A. (2022). Development and validation of Malaysian noise and chemical exposure questionnaire towards hearing among hospital workers. *The Medical Journal of Malaysia*, *77*(4), 420–426. https://pubmed.ncbi.nlm.nih.gov/35902930/

Restrepo, A. V, Valderrama, M. P., Correa, A. L., Mazo, L. M., González, N. E., & Jaimes, F. (2014). Implementation of the strategy “clean care is safer care” in a third level hospital in Medellín, Colombia. *Revista Chilena de Infectologia*, *31*(3), 280–286. https://doi.org/10.4067/S0716-10182014000300005

Ricke, I. J., Oglesby, A., Lyden, G. R., Barrett, E. S., Moe, S., & Nguyen, R. H. N. (2022). Knowledge, Attitudes, and Behaviors Regarding Chemical Exposure among a Population Sample of Reproductive-Aged Women. *International Journal of Environmental Research and*

Public Health, *19*(5). https://doi.org/10.3390/ijerph19053015

Rideout, K., Teschke, K., Dimich-Ward, H., & SM, K. (2005). Considering risks to healthcare workers from glutaraldehyde alternatives in high-level disinfection. *The Journal of Hospital Infection*, *59*(1), 4–11. https://doi.org/10.1016/j.jhin.2004.07.003

Rivers, J. K., Arlette, J. P., DeKoven, J., Guenther, L. C., Muhn, C., Richer, V., Rosen, N., Tremblay, J.-F., Wiseman, M. C., Zip, C., & Zloty, D. (2021). Skin care and hygiene among healthcare professionals during and after the SARS-CoV-2 pandemic. *SAGE Open Medicine*, *9*. https://doi.org/10.1177/20503121211062795

Roberts, M., Thygerson, S. M., Beard, J. D., Clark, C., & Montague, E. (2022). Occupational safety and health guidelines in relation to COVID-19 risk, death risk, and case-fatality proportion: An international, ecological study. *Health Science Reports*, *5*(2). https://doi.org/10.1002/hsr2.539

Robinson, G. L., Hitchcock, S., Kpadeh-Rogers, Z., Karikari, N., Johnson, J. K., Blanco, N., Morgan, D. J., Harris, A. D., & Leekha, S. (2019). Preventing Viral Contamination: Effects of Wipe and Spray-based Decontamination of Gloves and Gowns. *Clinical Infectious Diseases*, *69*, S228–S230. https://doi.org/10.1093/cid/ciz622

Rock, C., Hsu, Y. J., Curless, M. S., Carroll, K. C., Ross Howard, T., Carson, K. A., Cummings, S., Anderson, M., Milstone, A. M., & Maragakis, L. L. (2022). Ultraviolet-C Light Evaluation as Adjunct Disinfection to Remove Multidrug-Resistant Organisms. *Clinical Infectious Diseases*, *75*(1), 35–40. https://doi.org/10.1093/cid/ciab896

Rogers, C. A., Gaskin, S. E., Thredgold, L. D., & Pukala, T. L. (2023). An approach to quantify ortho-phthalaldehyde contamination on work surfaces. *Annals of Work Exposures and Health*, *67*(7), 886–894. https://doi.org/10.1093/annweh/wxad039

Rollins, S. M., Su, F.-C., Liang, X., Humann, M. J., Stefaniak, A. B., LeBouf, R. F., Stanton, M. L., Virji, M. A., & Henneberger, P. K. (2020). Workplace indoor environmental quality and asthma-related outcomes in healthcare workers. *American Journal of Industrial Medicine*, *63*(5), 417–428. https://doi.org/10.1002/ajim.23101

Rose, M. A., Garcez, T., Savic, S., & Garvey, L. H. (2019). Chlorhexidine allergy in the perioperative setting: a narrative review. *British Journal*

of Anaesthesia, *123*(1), e95–e103. https://doi.org/10.1016/j.bja.2019.01.033

Rustemeyer, T., Pilz, B., & PJ, F. (1994). [Contact allergies in medical occupations]. In *Der Hautarzt; Zeitschrift fur Dermatologie, Venerologie, und verwandte Gebiete* (Vol. 45, Issue 12, pp. 834–844). https://doi.org/10.1007/s001050050182

Rutala, W. A., & Weber, D. J. (2016). Monitoring and improving the effectiveness of surface cleaning and disinfection. *American Journal of Infection Control*, *44*(5), e69–e76. https://doi.org/10.1016/j.ajic.2015.10.039

Rutala, W. A., & Weber, D. J. (2019). Best practices for disinfection of noncritical environmental surfaces and equipment in health care facilities: A bundle approach. *American Journal of Infection Control*, *47*, A96–A105. https://doi.org/10.1016/j.ajic.2019.01.014

Rybka, A., Gavel, A., Kroupa, T., Meloun, J., Prazak, P., Draessler, J., Pavlis, O., Kubickova, P., Kratzerova, L., & Pejchal, J. (2021). Peracetic acid-based disinfectant is the most appropriate solution for a biological decontamination procedure of responders and healthcare workers in the field environment. *Journal of Applied Microbiology*, *131*(3), 1240–1248. https://doi.org/10.1111/jam.15041

Saito, R., Abbas Virji, M., Henneberger, P. K., Humann, M. J., Lebouf, R. F., Stanton, M. L., Liang, X., & Stefaniak, A. B. (2015). Characterization of cleaning and disinfecting tasks and product use among hospital occupations. *American Journal of Industrial Medicine*, *58*(1), 101–111. https://doi.org/10.1002/ajim.22393

Salomone, A., Bozzo, A., Di Corcia, D., Gerace, E., & Vincenti, M. (2018). Occupational exposure to alcohol-based hand sanitizers: The diagnostic role of alcohol biomarkers in hair. *Journal of Analytical Toxicology*, *42*(3), 157–162. https://doi.org/10.1093/jat/bkx094

Sanguinet, J., & Edmiston, C. (2021). Evaluation of dry hydrogen peroxide in reducing microbial bioburden in a healthcare facility. *American Journal of Infection Control*, *49*(8), 985–990. https://doi.org/10.1016/j.ajic.2021.03.004

Santovito, A., Cervella, P., & Delpero, M. (2014). Chromosomal damage in peripheral blood lymphocytes from nurses occupationally exposed to chemicals. *Human & Experimental Toxicology*, *33*(9), 897–903. https://doi.org/10.1177/0960327113512338

Sattar, S. A., Bradley, C., Kibbee, R., Wesgate, R., Wilkinson, M. A. C., Sharpe, T., & Maillard, J.-Y. (2015). Disinfectant wipes are appropriate to control microbial bioburden from surfaces: Use of a new ASTM standard test protocol to demonstrate efficacy. *Journal of Hospital*

Infection, *91*(4), 319–325. https://doi.org/10.1016/j.jhin.2015.08.026

Sauvé, J.-F., & Friesen, M. C. (2019). Using Decision Rules to Assess Occupational Exposure in Population-Based Studies. *Current Environmental Health Reports*, *6*(3), 148–159. https://doi.org/10.1007/s40572-019-00240-w

Schäferhenrich, A., Blümlein, K., Koch, W., Hahn, S., Schwarz, K., Poppek, U., Hebisch, R., Schlüter, U., Krug, M., & Göen, T. (2023). Inhalation and dermal exposure to biocidal products during foam and spray applications. *Annals of Work Exposures and Health*, *67*(7), 858–875. https://doi.org/10.1093/annweh/wxad037

Scheepers, P. T. J., Van Wel, L., Beckmann, G., & Anzion, R. B. M. (2017). Chemical characterization of the indoor air quality of a university hospital: Penetration of outdoor air pollutants. *International Journal of Environmental Research and Public Health*, *14*(5). https://doi.org/10.3390/ijerph14050497

Schnell, E., Karamooz, E., Harriff, M. J., Yates, J. E., Pfeiffer, C. D., & Smith, S. M. (2021). Construction and validation of an ultraviolet germicidal irradiation system using locally available components. *PLoS ONE*, *16*. https://doi.org/10.1371/journal.pone.0255123

Schnuch, A., Uter, W., Geier, J., Frosch, P. J., & Rustemeyer, T. (1998). Contact allergies in healthcare workers. Results from the IVDK. *Acta Dermato-Venereologica*, *78*(5), 358–363. https://doi.org/10.1080/000155598443060

Schöbel, H., Diem, G., Kiechl, J., Chistè, D., Bertacchi, G., Mayr, A., Wilflingseder, D., Lass-Flörl, C., & Posch, W. (2023). Antimicrobial efficacy and inactivation kinetics of a novel LED-based UV-irradiation technology. *Journal of Hospital Infection*, *135*, 11–17. https://doi.org/10.1016/j.jhin.2022.12.023

Schulte, P. A., Pandalai, S., Wulsin, V., & Chun, H. K. (2012). Interaction of occupational and personal risk factors in workforce health and safety. *American Journal of Public Health*, *102*(3), 434–448. https://doi.org/10.2105/AJPH.2011.300249

Sedeh, F. B., Michaelsdóttir, T. E., Christiensen, K. B., Mortensen, O. S., Jemec, G. B. E., & Ibler, K. S. (2023). Prevalence and risk factors for hand eczema among professional hospital cleaners in Denmark: A cross-sectional questionnaire-based study. *Contact Dermatitis*, *89*(4), 241–249. https://doi.org/10.1111/cod.14379

Seifi, T., & Reza Kamali, A. (2021). Antiviral performance of graphene-based materials with emphasis on COVID-19: A review. *Medicine in Drug Discovery*, *11*. https://doi.org/10.1016/j.medidd.2021.100099

Sellaoui, L., Badawi, M., Monari, A., Tatarchuk, T., Jemli, S., Luiz Dotto, G., Bonilla-Petriciolet, A., & Chen, Z. (2021). Make it clean, make it safe: A review on virus elimination via adsorption. *Chemical Engineering Journal*, *412*. https://doi.org/10.1016/j.cej.2021.128682

Shepherd, E., Leitch, A., & Curran, E. (2020). A quality improvement project to standardise decontamination procedures in a single NHS board in Scotland. *Journal of Infection Prevention*, *21*(6), 241–246. https://doi.org/10.1177/1757177420947477

Sher, M., & Mulder, R. (2020). Comparison of Aerosolized Hydrogen Peroxide Fogging with a Conventional Disinfection Product for a Dental Surgery. *Journal of Contemporary Dental Practice*, *21*(12), 1307–1311. https://doi.org/10.5005/jp-journals-10024-2983

Shi, H., Liang, K., Ali, R., Xu, S., & Ding, S. (2022). Injection Injury Caused by Disinfectant During COVID-19: A Case Report. *Frontiers in Public Health*, *10*. https://doi.org/10.3389/fpubh.2022.851175

Si, Y., Zhang, Z., Wu, W., Fu, Q., Huang, K., Nitin, N., Ding, B., & Sun, G. (2018). Daylight-driven rechargeable antibacterial and antiviral nanofibrous membranes for bioprotective applications. *Science Advances*, *4*(3). https://doi.org/10.1126/sciadv.aar5931

Siani, H., & Maillard, J.-Y. (2015). Best practice in healthcare environment decontamination. *European Journal of Clinical Microbiology and Infectious Diseases*, *34*(1), 1–11. https://doi.org/10.1007/s10096-014-2205-9

Siani, H., Wesgate, R., & Maillard, J.-Y. (2018). Impact of antimicrobial wipes compared with hypochlorite solution on environmental surface contamination in a health care setting: A double-crossover study. *American Journal of Infection Control*, *46*(10), 1180–1187. https://doi.org/10.1016/j.ajic.2018.03.020

Simmons, S. E., Carrion, R., Alfson, K. J., Staples, H. M., Jinadatha, C., Jarvis, W. R., Sampathkumar, P., Chemaly, R. F., Khawaja, F., Povroznik, M., Jackson, S., Kaye, K. S., Rodriguez, R. M., & Stibich, M. A. (2021). Deactivation of SARS-CoV-2 with pulsed-xenon ultraviolet light: Implications for environmental COVID-19 control. *Infection Control and Hospital Epidemiology*, *42*(2), 127–130. https://doi.org/10.1017/ice.2020.399

Simmons, S., Wier, G., Pedraza, A., & Stibich, M. (2021). Impact of a pulsed xenon disinfection system on hospital onset Clostridioides difficile infections in 48 hospitals over a 5-year period. *BMC Infectious Diseases*, *21*(1). https://doi.org/10.1186/s12879-021-06789-y

Singgih, S. I. R., Lantinga, H., Nater, J. P., Woest, T. E., & Kruyt-Gaspersz, J. A. (1986). Occupational hand dermatoses in hospital cleaning personnel. *Contact Dermatitis*, *14*(1), 14–19. https://doi.org/10.1111/j.1600-0536.1986.tb01146.x

Sit, G., Letellier, N., Iwatsubo, Y., Goldberg, M., Leynaert, B., Nadif, R., Ribet, C., Roche, N., Roquelaure, Y., Varraso, R., Zins, M., Descatha, A., Moual, N. L., & Dumas, O. (2021). Occupational exposures to organic solvents and asthma symptoms in the CONSTANCES cohort. *International Journal of Environmental Research and Public Health*, *18*(17). https://doi.org/10.3390/ijerph18179258

Sit, G., Varraso, R., Fezeu, L. K., Galan, P., Orsi, F., Pacheco Da Silva, E., Touvier, M., Hercberg, S., Paris, C., Le Moual, N., & Dumas, O. (2022). Occupational Exposures to Irritants and Sensitizers, Asthma and Asthma Control in the Nutrinet-Santé Cohort. *Journal of Allergy and Clinical Immunology: In Practice*, *10*(12), 3220-3227.e7. https://doi.org/10.1016/j.jaip.2022.08.047

SJ, S., McNamee, R., Turner, S., Carder, M., & RM, A. (2013). Assessing the impact of national level interventions on workplace respiratory disease in the UK: part 1--changes in workplace exposure legislation and market forces. *Occupational and Environmental Medicine*, *70*(7), 476–482. https://doi.org/10.1136/oemed-2012-101123

SJ, S., McNamee, R., Turner, S., Carder, M., & RM, A. (2015). The impact of national-level interventions to improve hygiene on the incidence of irritant contact dermatitis in healthcare workers: changes in incidence from 1996 to 2012 and interrupted times series analysis. *The British Journal of Dermatology*, *173*(1), 165–171. https://doi.org/10.1111/bjd.13719

Society of Gastroenterology Nurses and Associates. (1996). The safe and effective handling of glutaraldehyde solutions. In *Gastroenterology nursing : the official journal of the Society of Gastroenterology Nurses and Associates* (Vol. 19, Issue 5, p. suppl 1-7). https://pubmed.ncbi.nlm.nih.gov/9025390/

Sonday, Z., Baatjies, R., HH, M., & MF, J. (2023). Prevalence of work-related skin symptoms and associated factors among tertiary hospital workers exposed to cleaning agents in Southern Africa. *Contact Dermatitis*, *89*(3), 178–189. https://doi.org/10.1111/cod.14374

Song, X., Vossebein, L., & Zille, A. (2019). Efficacy of disinfectant-impregnated wipes used for surface disinfection in hospitals: A review. *Antimicrobial Resistance and Infection Control*, *8*(1). https://doi.org/10.1186/s13756-019-0595-2

Spencer, M., Vignari, M., Bryce, E., Johnson, H. B., Fauerbach, L., & Graham, D. (2017). A model for choosing an automated ultraviolet-C disinfection system and building a case for the C-suite: Two case reports. *American Journal of Infection Control*, *45*(3), 288–292. https://doi.org/https://doi.org/10.1016/j.ajic.2016.11.016

Starke, K. R., Friedrich, S., Schubert, M., Kämpf, D., Girbig, M., Pretzsch, A., Nienhaus, A., & Seidler, A. (2021). Are healthcare workers at an increased risk of obstructive respiratory diseases due to cleaning and disinfection agents? A systematic review and meta-analysis. *International Journal of Environmental Research and Public Health*, *18*(10), 5159. https://doi.org/10.3390/IJERPH18105159/S1

Steege, A. L., Boiano, J. M., & Sweeney, M. H. (2014). NIOSH Health and Safety Practices Survey of Healthcare Workers: Training and awareness of employer safety procedures. *American Journal of Industrial Medicine*, *57*(6), 640–652. https://doi.org/10.1002/ajim.22305

Stjärne Aspelund, A., Sjöström, K., Olsson Liljequist, B., Mörgelin, M., Melander, E., & Påhlman, L. I. (2016). Acetic acid as a decontamination method for sink drains in a nosocomial outbreak of metallo-β-lactamase-producing Pseudomonas aeruginosa. *Journal of Hospital Infection*, *94*(1), 13–20. https://doi.org/https://doi.org/10.1016/j.jhin.2016.05.009

Stoeva, I., Dencheva, M., Mircheva, K., & Chonin, A. (2020). Respiratory Symptoms of Exposure to Substances in the Workplace among Bulgarian Dental Students: A Self-report Questionnaire Survey. *Folia Medica*, *62*(1), 141–146. https://doi.org/10.3897/folmed.62.e48268

Su, F.-C., Friesen, M. C., Stefaniak, A. B., Henneberger, P. K., LeBouf, R. F., Stanton, M. L., Liang, X., Humann, M., & Abbas Virji, M. (2018). Exposures to volatile organic compounds among healthcare workers: Modeling the effects of cleaning tasks and product use. *Annals of Work Exposures and Health*, *62*(7), 852–870. https://doi.org/10.1093/annweh/wxy055

Su, F. C., Friesen, M. C., Humann, M., Stefaniak, A. B., Stanton, M. L., Liang, X., LeBouf, R. F., Henneberger, P. K., & Virji, M. A. (2019). Clustering asthma symptoms and cleaning and disinfecting activities and evaluating their associations among healthcare workers. *International Journal of Hygiene and Environmental Health*, *222*(5), 873–883. https://doi.org/10.1016/J.IJHEH.2019.04.001

Takigawa, T., & Endo, Y. (2006). Effects of glutaraldehyde exposure on human health. *Journal of Occupational Health*, *48*(2), 75–87. https://doi.org/10.1539/joh.48.75

Tang, H., Wang, H., Hamblin, M. R., Jiang, L., Zhou, Y., Xu, Y., & Wen, X. (2023). Contact dermatitis caused by prevention measures during the COVID-19 pandemic: a narrative review. *Frontiers in Public Health*, *11*. https://doi.org/10.3389/fpubh.2023.1189190

Tasar, R., Wiegand, C., & Elsner, P. (2021). How irritant are n-propanol and isopropanol? – A systematic review. *Contact Dermatitis*, *84*(1), 1–14. https://doi.org/10.1111/cod.13722

Teska, P., Gauthier, J., Lamb, J., & Hug, A. (2022). Powered air-purifying respirator (PAPR) disinfection and risk of surface damage from hydrogen peroxide and quaternary ammonium chloride-based disinfectants. *Journal of Occupational and Environmental Hygiene*, *19*(8), 449–454. https://doi.org/10.1080/15459624.2022.2088771

The GRADE Working Group. (2013). *GRADE handbook*. Handbook for Grading the Quality of Evidence and the Strength of Recommendations Using the GRADE Approach. https://gdt.gradepro.org/app/handbook/handbook.html

TL, G. (1987). Occupational health for hospital workers. *American Family Physician*, *35*(2), 137–142. https://pubmed.ncbi.nlm.nih.gov/2949570/

Tofanelli, M., Capriotti, V., Saraniti, C., Marcuzzo, A. V, Boscolo-Rizzo, P., & Tirelli, G. (2020). Disposable chlorine dioxide wipes for high-level disinfection in the ENT department: A systematic review. *American Journal of Otolaryngology - Head and Neck Medicine and Surgery*, *41*(3). https://doi.org/10.1016/j.amjoto.2020.102415

Tomb, R. M., White, T. A., Coia, J. E., Anderson, J. G., MacGregor, S. J., & Maclean, M. (2018). Review of the Comparative Susceptibility of Microbial Species to Photoinactivation Using 380–480 nm Violet-Blue Light. *Photochemistry and Photobiology*, *94*(3), 445–458. https://doi.org/10.1111/php.12883

Tyski, S., Bocian, E., & Laudy, A. E. (2022). Application of normative documents for determination of biocidal activity of disinfectants and antiseptics dedicated to the medical area: a narrative review. *Journal of Hospital Infection*, *125*, 75–91. https://doi.org/https://doi.org/10.1016/j.jhin.2022.03.016

Tyski, S., Grzybowska, W., & Bocian, E. (2021). Application of en 16615 (4-field test) for the evaluation of the antimicrobial activity of the selected commercial and self-made disinfectant wipes. *International Journal of Environmental Research and Public Health*, *18*(11). https://doi.org/10.3390/ijerph18115932

Vaidya, M., Gangakhedkar, G., Shetty, A., & Waghalkar, P. (2020). A rare occurrence of accidental exposure to UV radiation among operating theatre personnel. *Indian Journal of Anaesthesia*, *64*(3), 230–232. https://doi.org/10.4103/ija.IJA_656_19

van den Berg, R. B., de Poot, S., Swart, E. L., & Crul, M. (2021). Assessment of occupational exposure to nebulized isopropyl alcohol as disinfectant during aseptic compounding of parenteral cytotoxic drugs in cleanrooms. *Journal of Occupational and Environmental Hygiene*, *18*(8), 361–368. https://doi.org/10.1080/15459624.2021.1933505

VC, D., MM, C., Adisesh, A., & BN, S. (2003). Occupational allergic contact dermatitis in hospital workers caused by methyldibromo glutaronitrile in a work soap. In *Contact dermatitis* (Vol. 48, Issue 2, pp. 118–119). https://doi.org/10.1034/j.1600-0536.2003.480212_5.x

Versoza, M., Heo, J., Ko, S., Kim, M., & Park, D. (2020). Solid oxygen-purifying (SOP) filters: A self-disinfecting filters to inactivate aerosolized viruses. *International Journal of Environmental Research and Public Health*, *17*(21), 1–11. https://doi.org/10.3390/ijerph17217858

Viechtbauer, W. (2010). Conducting Meta-Analyses in R with the metafor Package. *Journal of Statistical Software*, *36*(3 SE-Articles), 1–48. https://doi.org/10.18637/jss.v036.i03

Vincent, M. J., Parker, A., & Maier, A. (2017). Cleaning and asthma: A systematic review and approach for effective safety assessment. *Regulatory Toxicology and Pharmacology*, *90*, 231–243. https://doi.org/10.1016/j.yrtph.2017.09.013

Vizcaya, D., Mirabelli, M. C., Orriols, R., Antó, J. M., Barreiro, E., Burgos, F., Arjona, L., Gomez, F., & Zock, J.-P. (2013). Functional and biological characteristics of asthma in cleaning workers. *Respiratory Medicine*, *107*(5), 673–683. https://doi.org/10.1016/j.rmed.2013.01.011

Walton, A. L., & Rogers, B. (2017). Workplace hazards faced by nursing assistants in the United States: A focused literature review. *International Journal of Environmental Research and Public Health*, *14*(5). https://doi.org/10.3390/ijerph14050544

Watanabe, R., Shimoda, T., Yano, R., Hayashi, Y., Nakamura, S., Matsuo, J., & Yamaguchi, H. (2014). Visualization of hospital cleanliness in

three Japanese hospitals with a tendency toward long-term care. *BMC Research Notes*, *7*, 121. https://doi.org/10.1186/1756-0500-7-121

WB, P., DE, C., DA, S., EA, N., Kasmer, J., & Noble, J. (1985). Occupational hazards to hospital personnel. *Annals of Internal Medicine*, *102*(5), 658–680. https://doi.org/10.7326/0003-4819-102-5-658

Weber, D. J., Rutala, W. A., Anderson, D. J., Chen, L. F., Sickbert-Bennett, E. E., & Boyce, J. M. (2016). Effectiveness of ultraviolet devices and hydrogen peroxide systems for terminal room decontamination: Focus on clinical trials. *American Journal of Infection Control*, *44*(5), e77–e84. https://doi.org/10.1016/j.ajic.2015.11.015

Weber, D. J., Rutala, W. A., Sickbert-Bennett, E. E., Kanamori, H., & Anderson, D. (2019). Continuous room decontamination technologies. *American Journal of Infection Control*, *47*, A72–A78. https://doi.org/10.1016/j.ajic.2019.03.016

Whitworth, K. W., Berumen-Flucker, B., Delclos, G. L., Fragoso, S., Mata, C., & Gimeno Ruiz de Porras, D. (2020). Job hazards and respiratory symptoms in Hispanic female domestic cleaners. *Archives of Environmental and Occupational Health*, *75*(2), 70–74. https://doi.org/10.1080/19338244.2019.1606774

Wilson, A. M., Mussio, I., Chilton, S., Gerald, L. B., Jones, R. M., Drews, F. A., LaKind, J. S., & Beamer, P. I. (2022). A Novel Application of Risk–Risk Tradeoffs in Occupational Health: Nurses' Occupational Asthma and Infection Risk Perceptions Related to Cleaning and Disinfection during COVID-19. *International Journal of Environmental Research and Public Health*, *19*(23). https://doi.org/10.3390/ijerph192316092

Wilson, A. M., O. Ogunseye, O., Fingesi, T., McClelland, D. J., Gerald, L. B., Harber, P., Beamer, P. I., & Jones, R. M. (2023a). Exposure frequency, intensity, and duration: What we know about work-related asthma risks for healthcare workers from cleaning and disinfection. *Journal of Occupational and Environmental Hygiene*, *20*(8), 350–363. https://doi.org/10.1080/15459624.2023.2221712

Wilson, A. M., O. Ogunseye, O., Fingesi, T., McClelland, D. J., Gerald, L. B., Harber, P., Beamer, P. I., & Jones, R. M. (2023b). Exposure frequency, intensity, and duration: What we know about work-related asthma risks for healthcare workers from cleaning and disinfection. *Journal of Occupational and Environmental Hygiene*, *20*(8), 350–363. https://doi.org/10.1080/15459624.2023.2221712

Wiszniewska, M., & Walusiak-Skorupa, J. (2014). Occupational allergy: respiratory hazards in healthcare workers. *Current Opinion in Allergy and Clinical Immunology*, *14*(2), 113–118. https://doi.org/10.1097/ACI.0000000000000039

WJ, E., RM, B., Lash, A., & CE, B. (1990). Neurotoxicological evaluation of hospital sterilizer workers exposed to ethylene oxide. *Journal of Toxicology. Clinical Toxicology*, *28*(1), 1–20. https://doi.org/10.3109/15563659008993472

Wright, J. R., Ly, T. T., Cromwell, K. B., Brislawn, C. J., Chen See, J. R., Anderson, S. L. C., Pellegrino, J., Peachey, L., Walls, C. Y., Lloyd, C. M., Jones, O. Y., Lawrence, M. W., Bess, J. A., Wall, A. C., Shope, A. J., & Lamendella, R. (2023). Assessment of a novel continuous cleaning device using metatranscriptomics in diverse hospital environments. *Frontiers in Medical Technology*, *5*. https://doi.org/10.3389/fmedt.2023.1015507

Xie, W., Dumas, O., Varraso, R., Boggs, K. M., Camargo, C. A., & Stokes, A. C. (2021). Association of Occupational Exposure to Inhaled Agents in Operating Rooms with Incidence of Chronic Obstructive Pulmonary Disease among US Female Nurses. *JAMA Network Open*. https://doi.org/10.1001/jamanetworkopen.2021.25749

Yang, D., Lin, L., Guo, P., Zhang, W., He, X., Huang, Q., Lai, L., & Long, W. (2017). Flashlight contamination and effectiveness of two disinfectants in a stomatology hospital. *International Journal of Nursing Sciences*, *4*(2), 169–172. https://doi.org/https://doi.org/10.1016/j.ijnss.2017.03.008

Yanke, E., Moriarty, H., Carayon, P., & Safdar, N. (2021). "The invisible staff": a qualitative analysis of environmental service workers' perceptions of the VA clostridium difficile prevention bundle using a human factors engineering approach. *Journal of Patient Safety*, *17*(8), E806–E814. https://doi.org/10.1097/PTS.0000000000000500

Ye, G., Lin, H., Chen, S., Wang, S., Zeng, Z., Wang, W., Zhang, S., Rebmann, T., Li, Y., Pan, Z., Yang, Z., Wang, Y., Wang, F., Qian, Z., & Wang, X. (2020). Environmental contamination of SARS-CoV-2 in healthcare premises. *The Journal of Infection*, *81*(2), e1–e5. https://doi.org/10.1016/j.jinf.2020.04.034

Youssef, D., Abou-Abass, L., & Hassan, H. (2023). Unveiling the unknown: first comprehensive assessment of the knowledge, attitudes and practices of hospital cleaning services staff regarding COVID-19 in Lebanon during the pandemic. *Archives of Public Health*, *81*(1).

https://doi.org/10.1186/s13690-023-01149-5

Yüksel, Y. T., Nørreslet, L. B., Flachs, E. M., Ebbehøj, N. E., & Agner, T. (2022). Hand eczema, wet work exposure, and quality of life in health care workers in Denmark during the COVID-19 pandemic. *JAAD International*, *7*, 86–94. https://doi.org/10.1016/j.jdin.2022.02.009

Yüksel, Y. T., Sonne, M., Nørreslet, L. B., Gundersen, G., Fazli, M. M., & Agner, T. (2022). Skin barrier response to active chlorine hand disinfectant—An experimental study comparing skin barrier response to active chlorine hand disinfectant and alcohol-based hand rub on healthy skin and eczematous skin. *Skin Research and Technology*, *28*(1), 89–97. https://doi.org/10.1111/srt.13096

Zahrallayali, A., Al-Doboke, A., Alosaimy, R., Alabbasi, R., Alharbi, S., Fageeh, S., Altayyar, S., & Azher, R. (2021). The prevalence and clinical features of skin irritation caused by infection prevention measures during covid-19 in the mecca region, saudi arabia. *Clinical, Cosmetic and Investigational Dermatology*, *14*, 889–899. https://doi.org/10.2147/CCID.S309681

Zhang, X.-B., Wei, Y.-L., Zhao, G., He, M., Sun, J., & Zeng, W. (2023). Coronavirus disease 2019: Repeated immersion of chlorine-containing disinfectants has adverse effects on goggles. *Frontiers in Public Health*, *11*. https://doi.org/10.3389/fpubh.2023.1016938
